# Supplementary material for: Molecular basis for azetidine-2-carboxylic acid biosynthesis
Source: Nat Commun. 2025 Feb 4;16:1348. doi: 10.1038/s41467-025-56610-6 (PMC11794875; doi:10.1038/s41467-025-56610-6)
Supplement: Supplementary file 1 — Supplementary Information [file 41467_2025_56610_MOESM1_ESM.pdf]

Supplementary Information

Molecular basis for azetidine-2-carboxylic acid biosynthesis

Tim J. Klaubert,[1] Jonas Gellner,[1,2,*] Charles Bernard,[3,10,*] Juliana Effert,[4,*] Carine Lombard,[3] Ville R. I. Kaila,[2] Helge B. Bode,[4,5,6,7,8,9] Yanyan Li,[3,#] Michael Groll[1,#]

[1] Center for Protein Assemblies, Department Bioscience, School of Natural Sciences, Technical University Munich, Garching, Germany.

[2] Department of Biochemistry and Biophysics, Stockholm University, Stockholm, Sweden.

[3] Laboratory Molecules of Communication and Adaptation of Microorganisms (MCAM), UMR 7245 CNRS-MNHN (Muséum National d'Histoire Naturelle), Paris, France.

[4] Max Planck Institute for Terrestrial Microbiology, Department of Natural Products in Organismic Interactions, Marburg, Germany.

[5] Institute of Molecular Biosciences, Goethe University Frankfurt, Frankfurt am Main, Germany.

[6] Myria Biosciences AG, Basel, Switzerland.

[7] Department of Chemistry, Philipps-University Marburg, Marburg, Germany.

[8] LOEWE Centre for Translational Biodiversity Genomics (LOEWE TBG) & Senckenberg Gesellschaft für Naturforschung, Frankfurt am Main, Germany.

[9] Center for Synthetic Microbiology (SYNMIKRO), Phillips University Marburg, Marburg, Germany.

[10] Current address: Institut Pasteur, CNRS UMR3525, Microbial Evolutionary Genomics, Paris, France.

* Equal contribution

[#] Correspondence should be addressed to:

yanyan.li@mnhn.fr

michael.groll@tum.de

Supplementary Table S1. Plasmids used in this work

Plasmids	Genotype[a]	Reference
pETDuet_SUMO_Ser_AzeJ[b]	ori ColE1, Amp^R, $P_{T7,}$ *azeJ*	this study
pETDuet_SUMO_VioH	ori ColE1, Amp^R, $P_{T7,}$ *vioH*	this study
pCK_0431 pACYC_ara	ori p15A, cm^R, *araC*, P_{BAD} and tacI, I-SceI, I-CeuI	this study
pCK_0433 pCOLA_ara	ori ColA, kanR, araC, PBAD, tacI, I-SceI, I-CeuI, minus BsaI	this study
pACYC_pxaA	ori p15A, cm^R, *araC-P_{BAD}, pxaA*	this study
pACYC_pxaAB	ori p15A, cm^R, *araC-P_{BAD}, pxaAB*	this study
pCOLA_azeJ	ori ColA, kan^R, *araC-P_{BAD}, azeJ*	this study
pCOLA_vioH	ori ColA, kan^R, *araC-P_{BAD}, vioH*	this study

a: Amp^R, ampicillin resistance; cm^R, chloramphenicol resistance; kan^R, kanamycin resistance
b: This construct served as the basis for mutagenesis, generating a series of expression plasmids for AzeJ variants.

Supplementary Table S2. Primer sequences for *azeJ* mutagenesis with modified positions highlighted in red.

Mutant	Forward primer (5' → 3')	Reverse primer (3' → 5')
Y18A	A TCC GCG TTC GCG GAG CTG GAG TTT G	TC GCG ATG TGT GCC AAT G
Y18F	A TCC GCG TTC TTT GAG CTG GAG T	TC GCG ATG TGT GCC AAT G
M28N	A CGT ACG ATC AAC ACC CTG GCG AAC	GG GCC AAA CTC CAG CTC A
F134Y	C CTG TTG CCG TAT AAT CTC CTG GG	TA CAG TGC GCG ACT TCC G
F134H	C CTG TTG CCG CAT AAT CTC CTG G	TA CAG TGC GCG ACT TC
F134L	C CTG TTG CCG CTG AAT CTC CTG G	TA CAG TGC GCG ACT TCC G
L136N	G CCG TTC AAT AAC CTG GGA AAC TTT C	AACAGGTACAGTGCGCGA
R172A	T ACT CGC GTA GCG CAG TCG TAC TAT C	GC TTC GGC ACT ATC C
R172K	T ACT CGC GTA AAA CAG TCG TAC TAT C	GC TTC GGC ACT ATC C
R172Q	T ACT CGC GTA CAG CAG TCG TAC TAT C	GC TTC GGC ACT ATC C
L136D	G CCG TTC AAT GAT CTG GGA AAC TTT CGG	AA CAG GTA CAG TGC GCG A
Y175A	A CGT CAG TCG GCG TAT CGT CGC TG	AC GCG AGT AGC TTC
Y175F	A CGT CAG TCG TTT TAT CGT CGC TG	AC GCG AGT AGC TTC

Supplementary Table S3. Primers used for cloning.

Plasmids	Oligonucleotides	Sequence (5' →3'; overlapping ends)	Template
pACYC_pxaA	JE_G_for3	GTTTTTTTGGGCTAACAGGAGGAATTCCATGAAAACTTCACAATTAGTACCTCTTACC	*X. stockiae* DSM17904
	JE_G_rev5	GCAGCAGCCTAGGTTAATTAATCAATGGACGGGACATATTTTCTCC	
	ck1011	TTAATTAACCTAGGCTGCTGCCAC	pCK_0431
	ck1008	CATGGAATTCCTCCTGTTAGCCCAA	pACYC_ara
pACYC_pxaAB	JE_G_for3	GTTTTTTTGGGCTAACAGGAGGAATTCCATGAAAACTTCACAATTAGTACCTCTTACC	*X. stockiae* DSM17904
	JE_G_rev6	GCAGCAGCCTAGGTTAATTAATTATAGGGTCTGGAGAGTAAGATCTC	
	ck1011	TTAATTAACCTAGGCTGCTGCCAC	pCK_0431
	ck1008	CATGGAATTCCTCCTGTTAGCCCAA	pACYC_ara
pCOLA_azeJ	azeJ_for	TTGGGCTAACAGGAGGAATTCCATGTCCCAGAACATGGACCTGAC	pETDuet_SUMO_Ser_AzeJ
	azeJ_rev	GGTGGCAGCAGCCTAGGTTAATTAATTACGCGGCTGAGCTACCAAAG	
	ck1011	TTAATTAACCTAGGCTGCTGCCAC	pCK_0433
	ck1008	CATGGAATTCCTCCTGTTAGCCCAA	pCOLA_ara
pCOLA_vioH	vioH_for	TTGGGCTAACAGGAGGAATTCCATGTCCACCCTGGACTTCTACG	pETDuet_SUMO_VioH
	vioH_rev	GGTGGCAGCAGCCTAGGTTAATTAATTACGGACGCCACGGTTCG	
	ck1011	TTAATTAACCTAGGCTGCTGCCAC	pCK_0433
	ck1008	CATGGAATTCCTCCTGTTAGCCCAA	pCOLA_ara
pCK_0431	ck0922	TAACTATAACGGTCCTAAGGTAGCGAACCTCAGGCATTTGAGAAGCACAC	pCK_0401
	ck0953	AATTACCCTGTTATCCCTACCCAATACGCAAACCGCCTC	
	ck0951	GTAGGGATAACAGGGTAATTATGACAACTTGACGGCTACATCATTCAC	pCK_0401[1]
	ck0922-rev	CGCTACCTTAGGACCGTTATAGTTAGCAAAAAACCCCTCAAGACCC	
pCK_0433	ck0952	CTAGGGATAACAGGGTAATTATGACAACTTGACGGCTACATCATTCAC	pCK_0406[2]
	ck0922rev	AATTACCCTGTTATCCCTAGTCTAGAGTGATGGTGTCGGGAATC	
	ck0960	CGGGTCTTGAGGGGTTTTTTGCTAACTATAACGGTCCTAAGGTAGCGAAG	pCK_0406
	ck0955	AATTACCCTGTTATCCCTAGTCTAGAGTGATGGTGTCGGGAATC	

Supplementary Table S4. Crystallographic data collection and refinement statistics.

	AzeJ:SAH	AzeJ:MTA:AZE	AzeJ:MTA:AZE	VioH:SAH
**Crystal parameters**				
Space group	$P2_12_12$	$P2_12_12$	$P4_222$	$P2_12_12$
Cell constants	a= 141.5 Å	a= 142.6 Å	a=b= 122.2 Å	a= 60.0 Å
	b= 84.2 Å	b= 82.2 Å	c= 87.1 Å	b= 77.6 Å
	c= 94.3 Å	c= 94.7 Å		c= 84.4 Å
Subunits / AU[a]	4	4	2	2
**Data collection**				
Beam line	X06SA, SLS	X06SA, SLS	X06SA, SLS	X06SA, SLS
Wavelength (Å)	1.0	1.0	1.0	1.0
Resolution range (Å)[b]	30-3.0 (3.1-3.0)	30-1.95 (2.05-1.95)	30-1.95 (2.05-1.95)	30-2.1 (2.2-2.1)
No. observations	119,534	357,752	268,774	77,220
No. unique reflections[c]	21,945	80,170	48,492	21,394
Redundancy	5.4	4.5	5.5	3.6
Completeness (%)[b]	94.3 (97.1)	98.1 (98.7)	99.8 (100)	93.5 (92.7)
R_{merge} (%)[b, d]	9.3 (66.3)	5.8 (68.8)	6.3 (69.7)	9.9 (54.0)
I/σ (I)[b]	14.6 (2.7)	16.3 (2.6)	17.1 (2.4)	8.2 (2.8)
**Refinement (REFMAC5)**				
Resolution range (Å)	30-3.0	30-1.95	30-1.95	30-2.1
No. refl. working set	20,801	76,143	346,038	20,315
No. refl. test set	1,095	4,008	2,423	1,069
No. non hydrogen atoms	7,226	7,754	3,822	3,836
No. of ligand atoms	104	108	47	52
Solvent molecules	14	302	253	62
R_{work}/R_{free} (%)[e]	19.3 / 24.2	16.4 / 20.9	14.9 / 19.7	19.7 / 24.9
r.m.s.d. bond (Å) / angle (°)[f]	0.001 / 1.1	0.004 / 1.2	0.004 / 1.2	0.003 / 1.2
Average B-factor ($Å^2$)	71.0	37.7	35.5	24.5
Ramachandran Plot (%)[g]	97.3 / 2.7 / 0	98.4 / 1.6 / 0	98.4 / 1.6 / 0	97.2 / 2.8 / 0
PDB accession code	8RYD	8RYE	8RYF	8RYG

[a] Asymmetric unit

[b] The values in parentheses for resolution range, completeness, R_{merge} and I/σ (I) correspond to the highest resolution shell

[c] Data reduction was carried out with XDS and from a single crystal. Friedel pairs were treated as identical reflections

[d] $R_{merge}(I) = \Sigma_{hkl}\Sigma_j \mid I(hkl)_j - \langle I(hkl)\rangle \mid / \Sigma_{hkl} \Sigma_j I(hkl)_j$, where $I(hkl)_j$ is the jth measurement of the intensity of reflection hkl and $\langle I(hkl)\rangle$ is the average intensity

[e] $R = \Sigma_{hkl} \mid |F_{obs}| - |F_{calc}| \mid / \Sigma_{hkl} |F_{obs}|$, where R_{free} is calculated without a sigma cut off for a randomly chosen 5% of reflections, which were not used for structure refinement, and R_{work} is calculated for the remaining reflections

[f] Deviations from ideal bond lengths / angles

[g] Number of residues in favored region / allowed region / outlier region

Supplementary Table S5. Overview of DFT cluster models. All energies are reported in kcal mol^{-1}. Free energy values highlighted in bold correspond to those reported in Fig. 3. Abbreviations introduced here are used throughout the Supplement to identify DFT models.

System	HCY amine	Mutation	Number of Atoms	Total Charge	Abbreviation	TS[a]		Product[a]	
						ΔE_{el}	ΔG	ΔE_{el}	ΔG
**AzeJ** (ε=4)	-NH_2	WT	252	-1	*AzeJ_WT*	16.4[b] (20.7)	**16.6** (20.9)	-2.3[c] (-4.1)	**-1.2** (-2.9)
		F134L	251	-1	*AzeJ_F134L*	17.8	**17.2**	1.4	2.2
		L136D	245	-2	*AzeJ_L136D*	19.3	**18.8**	2.7	3.9
		Y175F	251	-1	*AzeJ_Y175F*	18.9	**18.7**	2.8	3.5
	-NH_3^+	WT	253	0	*AzeJ_pWT*	–	–	36.7 (35.7)	35.6 (34.6)
**Isolated Ligand** (ε=4)	-NH_2	–	50	0	*Lig_Hyd*	19.3 (24.1)	21.5 (26.3)	-7.9 (-9.2)	-6.1 (-7.4)
	-NH_3^+[d]		51	+1	*pLig_Hyd*	30.6 (35.5)	29.3 (34.2)	10.9 (9.7)	10.3 (9.2)
**Isolated Ligand** (ε=80)	-NH_2	–	50	0	*Lig_Wat*	20.8 (25.3)	**18.4** (22.9)	-2.8 (-3.9)	-5.3 (-6.5)
	-NH_3^+[e]		51	+1	*pLig_Wat*	29.7 (32.7)	26.8 (29.8)	14.3 (13.3)	12.7 (11.7)
**VioH** (ε=4)	-NH_2	WT	304	-1	*VioH_WT*	26.2 (31.1)	23.3 (28.3)	-5.4 (-7.0)	-4.4 (-6.0)

[a] Energies were calculated based on optimised geometries and are given relative to the reactant state. Electronic energies (E_{el}) were calculated with the B3LYP-D3 (and ωB97X-D in brackets) functionals, using the def2-TZVP basis set and COSMO solvation model with dielectric constants given in column 1. Free energies (G) include vibrational and entropic corrections, calculated at the B3LYP-D3-RI/def2-SVP level using the COSMO solvation model (Methods).

[b] B3LYP-D3/def2-QZVP: ΔE_{el} = 16.7 kcal mol^{-1}

[c] B3LYP-D3/def2-QZVP: ΔE_{el} = -2.2 kcal mol^{-1}

[d] Geometry optimisation of the reactant state led to spontaneous proton transfer from the HCY amine to the carboxylate. Thus, the -COOH model was considered as reactant state.

[e] Energies of the second step in ring formation (S_N2) are given relative to the reactant state (-NH_3^+). The first step, proton transfer to the SAM-carboxylate, has a free energy barrier of ΔG = 1.8 kcal mol^{-1} (both B3LYP-D3 and ωB97X-D with def2-TZVP basis set); the resulting intermediate (-COOH) has a free energy of ΔG = -0.4 kcal mol^{-1} (B3LYP-D3/ def2-TZVP) or ΔG = -0.2 kcal mol^{-1} (ωB97X-D/ def2-TZVP).

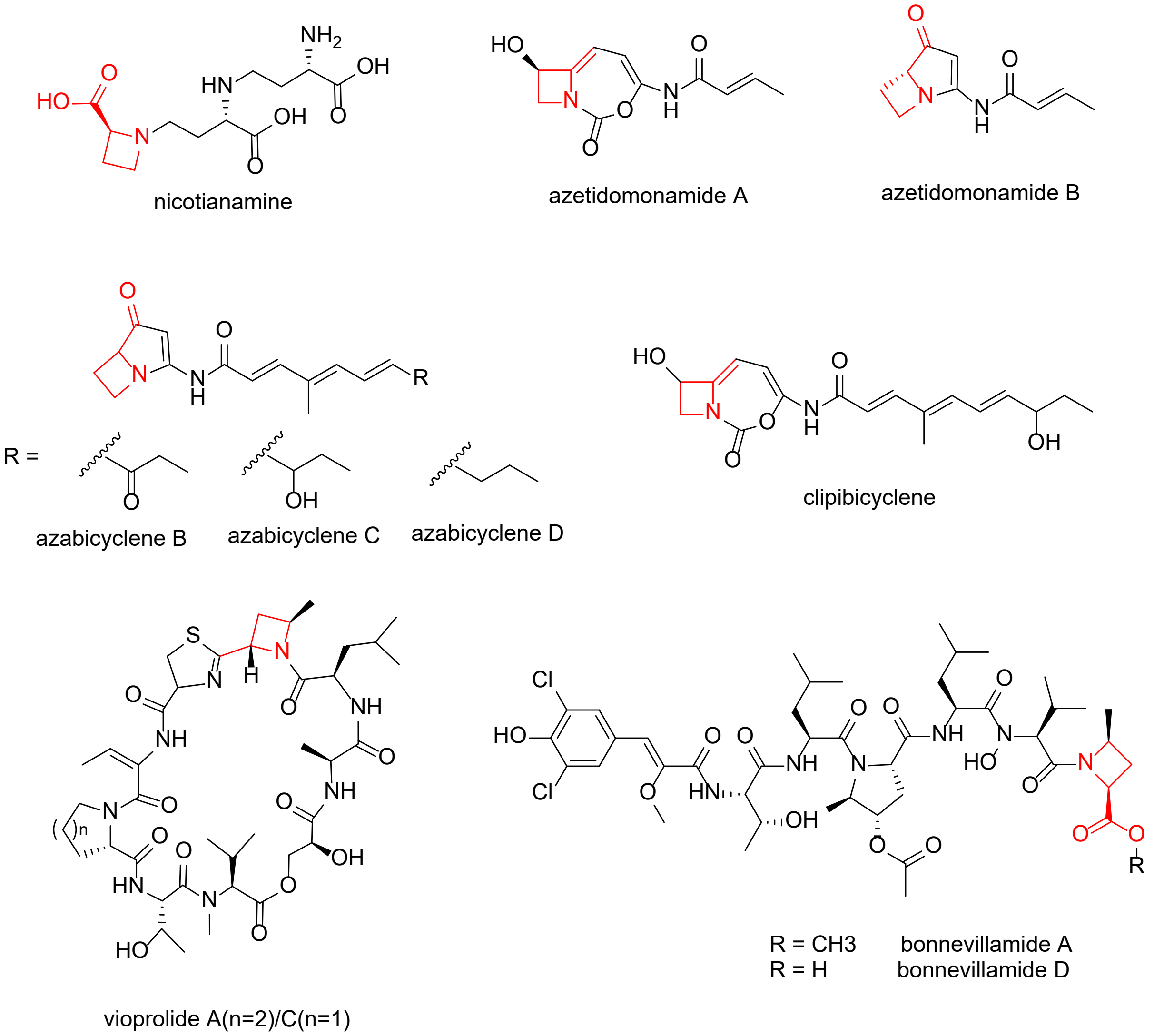

Fig. S1. Natural products containing the azetidine-2-carboxylic acid-derived motif (highlighted in red).

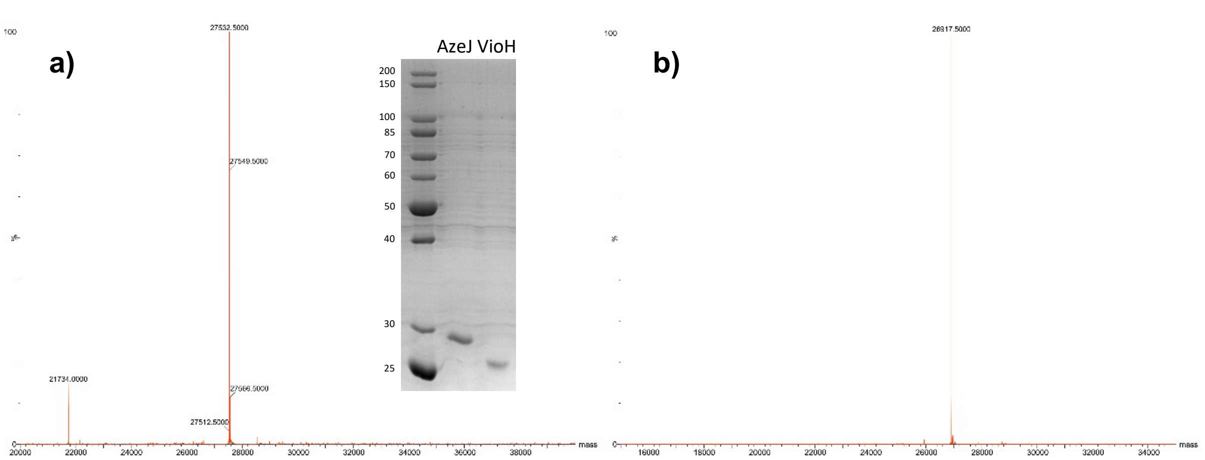

Fig. S2. ESI-MS analysis of **a)** AzeJ (27.533 kDa) and **b)** VioH (26.917 kDa). Both enzymes were expressed without the start methionine.

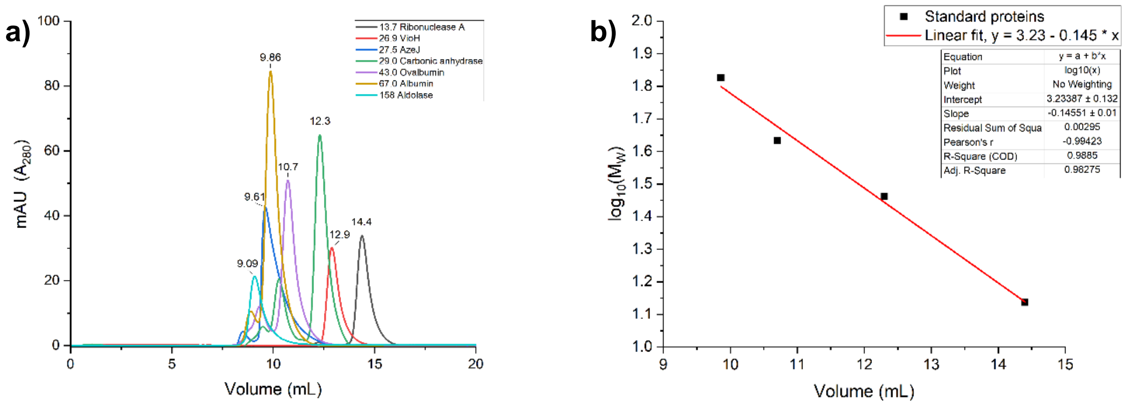

Fig. S3. AzeJ forms a homodimer and VioH is monomeric a) Superdex 75 Increase 10/300 GL (Cytiva) elution profiles of AzeJ (27.5 kDa, 6.5 mg/mL, blue) and VioH (26.9 kDa, 5 mg/mL, red) compared with marker proteins:ribonuclease A (13.7 kDa, 2 mg/mL, black), carbonic anhydrase (29.0 kDa, 3 mg/mL, green), ovalbumin (43.0 kDa, 6 mg/mL, purple), albumin (67.0 kDa, 7 mg/mL, orange), and aldolase (158 kDa, 5 mg/mL, turquoise. Protein absorbance was measured at 280 nm. **b)** Calibration curve illustrating the correlation between elution volume and molecular mass (M_W, log_{10}). The red line represents the linear regression fit derived from marker proteins, illustrating AzeJ as a homodimer and VioH as a monomer.

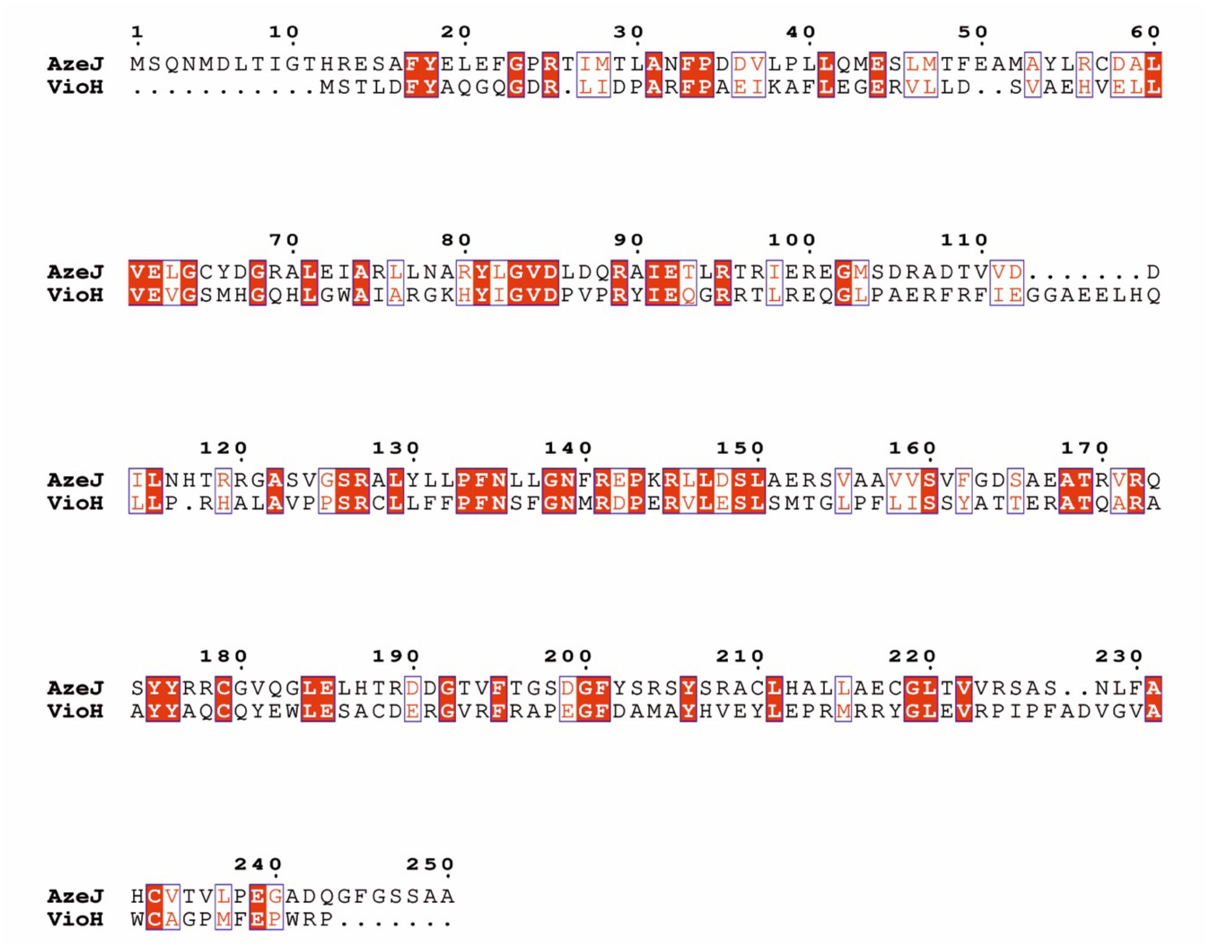

Fig. S4. Sequence alignment of AzeJ and VioH. Identical residues are highlighted in red boxes and similar ones are shown in red capitals.

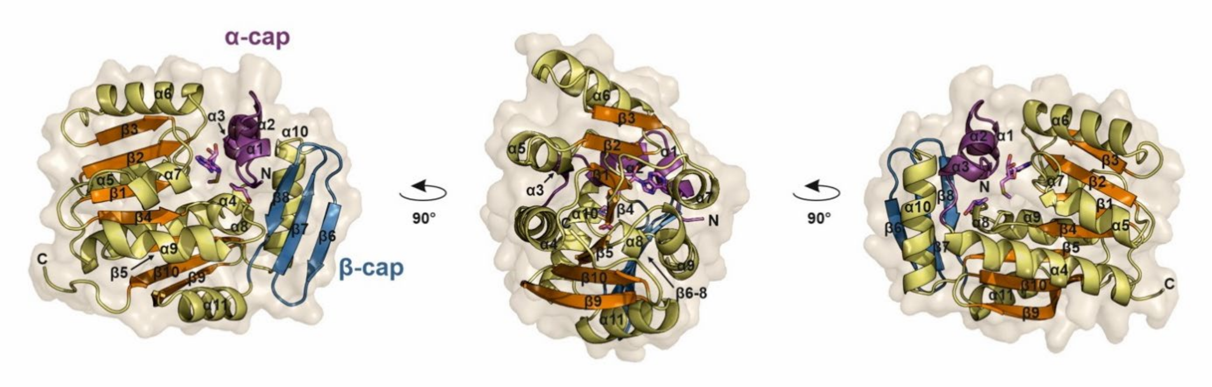

Fig. S5. Secondary structure elements of AzeJ in complex with MTA and AZE. The Rossman fold, serving as the nucleotide-binding domain, features a seven-stranded beta sheet (β3↑, β2↑, β1↑, β4↑, β5↑, β10↓, β9↑, orange) surrounded by helices α4-6 and α8-9, as well as α11. The active site, flanked by helices α1-3 (purple) and strands β6-8 (blue), switches to a closed state upon ligand binding.

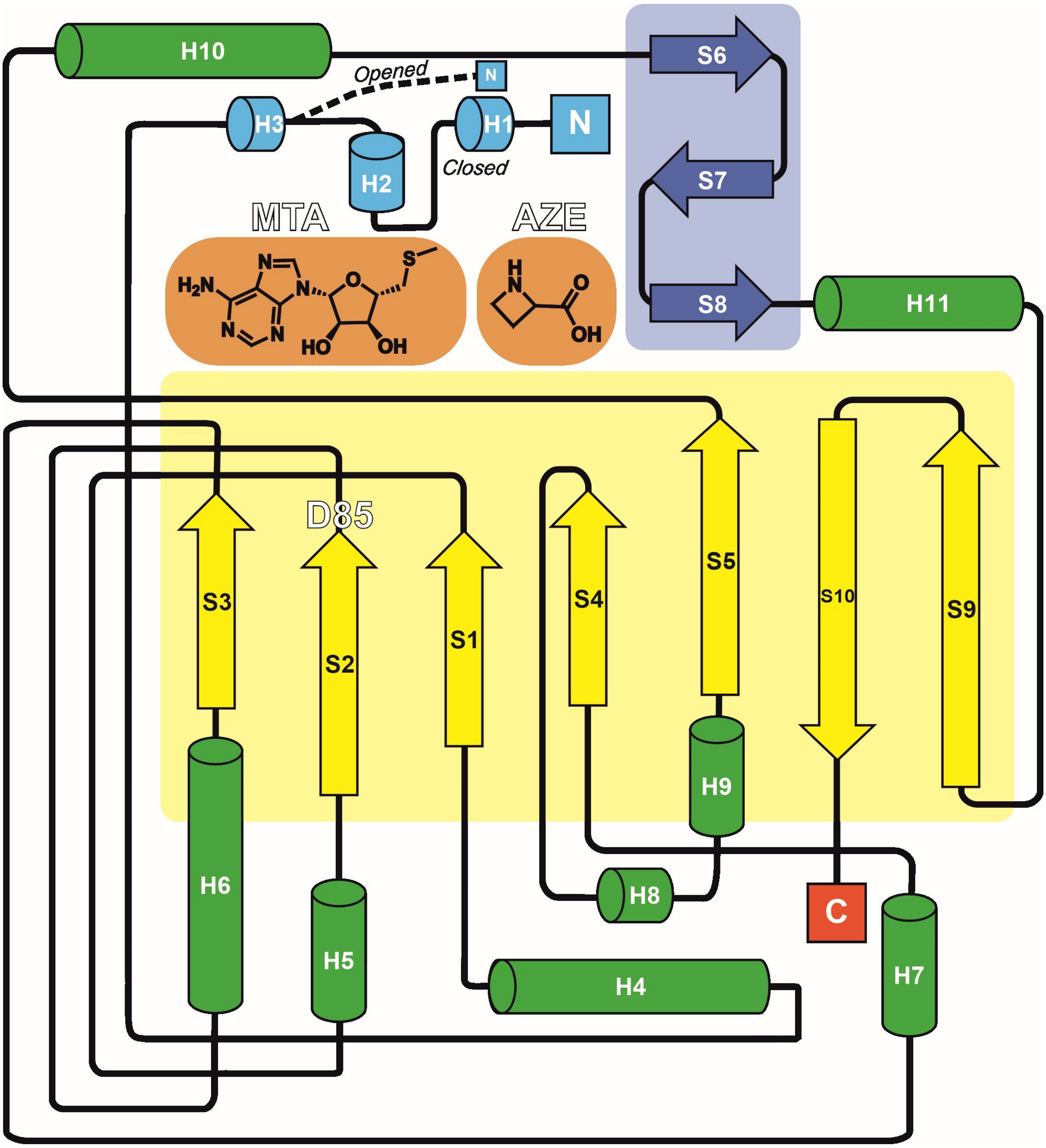

Fig. S6. Topology of the AzeJ:MTA:AZE complex (PDB ID: 8RYE, chain A). Beta sheets are shown by arrows (S1-S10) and helices are represented by barrels (H1-H11). The characteristic Rossmann fold (yellow) is flanked by helices H4-H9 and H11 (green). Asp85, positioned at the end of S2, coordinates the ribose group. The active site containing MTA and AZE is enclosed by the N-terminal helices H1 and H3 (light blue) as well as the beta-sheets S6-S8 (dark blue). H1 and H2 might regulate the conformation of AzeJ between the open state (dashed line) and the closed state.

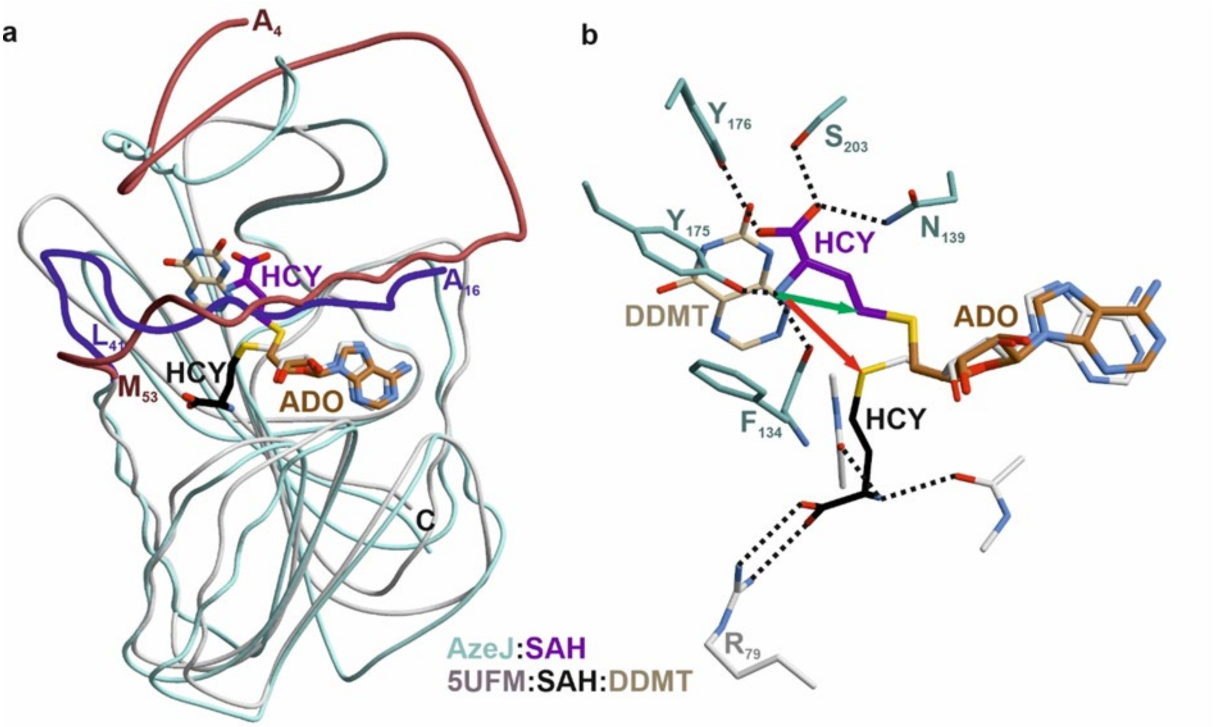

Fig. S7. Structural comparison of AzeJ (PDB ID: 8RYD) and 1,6-Didesmethyltoxoflavin N-Methyltransferase (PDB ID: 5UFM). The 5UFM complex, bound to *S*-adenosyl-*L*-homocysteine (SAH; homocysteine (HCY), C-atoms in black; adenosine (ADO), C-atoms in white) and its substrate 1,6-didemethyltoxoflavin (DDMT, C-atoms in tan) [3], is the best hit in the structural homology search of AzeJ (HCY, C-atoms in purple; ADO, C-atoms in brown). Calculations were performed using the DALI server[4] and resulted in a Z-score of 19.2 (sequence identity = 19%). **a)** Structural superposition of AzeJ and 5UFM in coil representation (rmsd = 1.4 Å, 124 C^{α}-atoms; AzeJ in cyan, 5UFM in white; SAH and DDMT shown as sticks). The N-terminus of AzeJ and 5UFM vary significantly (AzeJ, Ala16-Leu41, in blue; 5UFM, Ala4-Met53, in brown). **b)** Active site comparison: Close-up view of AzeJ and 5UFM. The adenosine residue aligns perfectly, whereas the HCY side chains occupy distinct positions. In AzeJ, HCY is oriented similar as the substrate DDMT in 5UFM. Side chain residues coordinating the amine and carboxyl groups of SAH are illustrated (AzeJ, C-atoms in cyan; 5UFM, C-atoms in white), and H-bonds are shown in black dots. Note that AZE-ring formation in AzeJ and methyl transfer in 5UFM take place in the same region. The NH_2 attack on the C^{γ} in AzeJ is highlighted by a green arrow; the methyl transfer in 5UFM is shown by a red arrow.

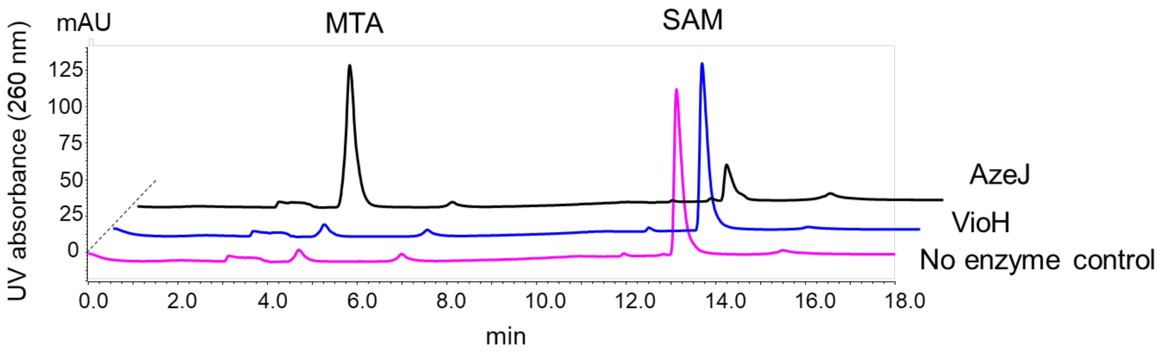

Fig. S8. *In vitro* activity profiles of AzeJ and VioH. The HPLC chromatograms were recorded with UV detection at 260 nm. Assays were performed in 50 mM Tris-HCl (pH 7.2) and supplemented with 1 mM SAM, 2 mM TCEP and 3 µM enzyme. The incubation time was 1.5 h at 30 °C.

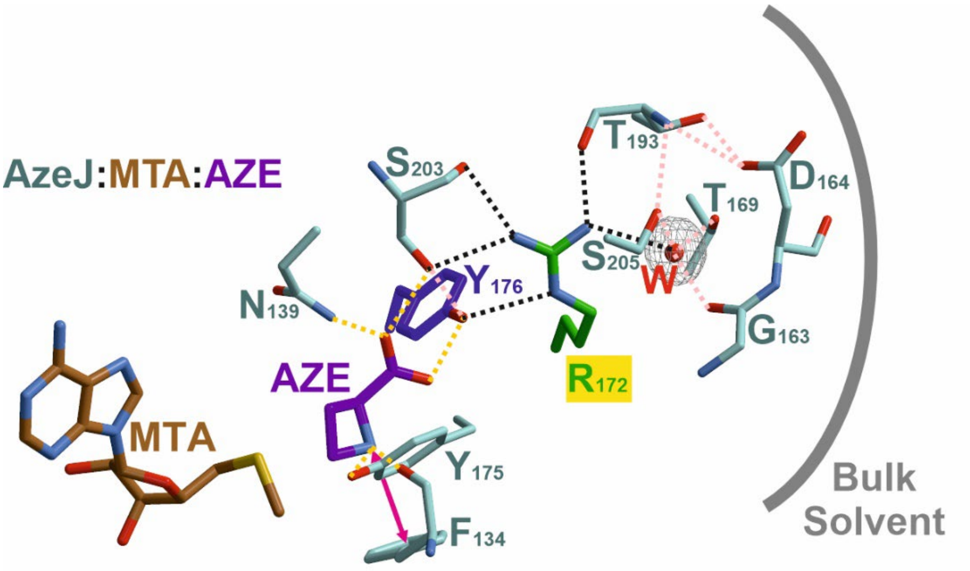

Fig. S9. Hydrogen bonding network in the AzeJ:MTA:AZE complex supporting SAM deprotonation. The structure of AzeJ:MTA:AZE (PDB ID 8RYE) reveals an intricate hydrogen bonding network involved in catalysis. The carboxylate of AZE (purple) is stabilized by three hydrogen bonds, while the AZE-NH_2^+ group forms two characteristic H-bonds (shown in gold). The positively charged nitrogen of AZE is additionally stabilized by cation-π interactions with Phe134 (double arrow in magenta). Upon binding, the proton of SAM-NH_3^+ would be transferred to Arg172 (green) via Tyr176 (blue). The guanidine side chain of Arg172 forms five distinct H-bonds (highlighted in black), notably with a water molecule (W, red sphere), as defined by the $2F_o$-F_c electron density map (grey mesh, contoured at 1σ). This suggests that Arg172 could provide a gating residue central mediating the proton transfer to the bulk solvent. H-bonds ≤ 3 Å are illustrated, with H-bonds not further discussed shown in pink.

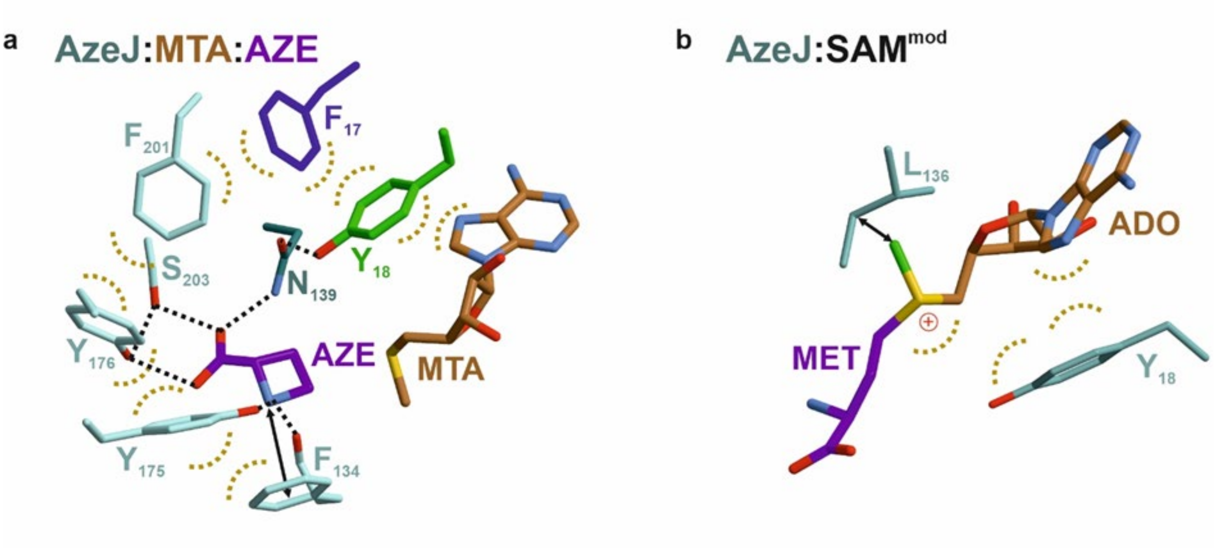

Fig. S10. **Tyr18 in AzeJ: A potential gating residue involved in a precise amino acid interplay at the active centre. a)** The catalytic chamber consists of a network of aromatic amino acids interacting through robust π-π interactions (dashed arcs in yellow). Here, the π-π stacking between Tyr18 (C-atoms in green) and the upstream Phe17 (C-atoms in blue) is conspicuous, as both residues are unstructured in the open conformation of the AzeJ:MTA complex (PDB ID: 8RYF, chain B). In addition, Tyr18 forms π-π interactions with the adenine residue of MTA (C-atoms in brown) and is hydrogen-bonded to the carboxylate group of AZE (C-atoms in purple) via Asn139 (C-atoms in dark cyan). Except for Ser203 and Asn139, all amino acids (C-atoms in cyan) forming H-bonds with AZE are coordinated via π-π interactions and form a rigid framework. **b)** In the AzeJ:SAH structure (PDB ID: 8RYD, chain A), the sulfur atom has been extended by a methyl group (green), modelling the positively charged substrate SAM^{mod} (composed of adenosine (ADO) and methionine (MET)). While the methyl group is in close van der Waals distance to Leu136 (black arrow), Tyr18 forms strong π-π interactions with the adenine residue of ADO and cation-π interactions with the sulfonium group of SAM^{mod}.

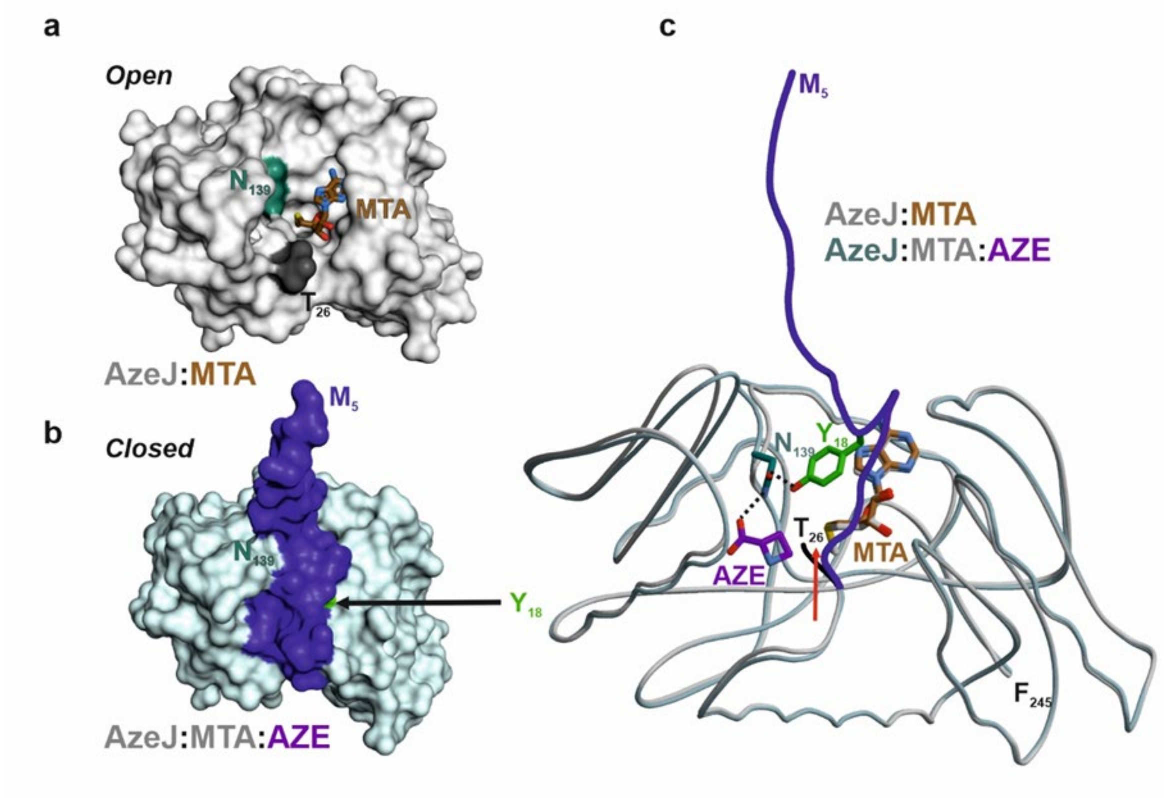

Fig. S11. Conformational dynamics of AzeJ between open and closed states during catalysis. The co-crystallisation of AzeJ with its substrate SAM resulted in two datasets with distinct crystal lattices. **a)** In space group $P4_222$ (PDB ID: 8RYF), chain B shows an accessible active site. Protein residues are depicted as a surface model (white). Asn139 is highlighted in dark cyan and MTA is represented in sticks (C-atoms in brown). The N-terminus starts with Thr26 (C-atoms in black), and the first 25 amino acids of the subunit are disordered (open state). **b)** In space group $P2_12_12$ (PDB ID: 8RYE), chain A is complexed with MTA and AZE. The N-terminus is well-defined in the electron density map and sequesters the active site from bulk solvents (Met5 - Thr26, blue; Thr26 - Phe245, cyan). Asn139 and Tyr18 (C-atoms in green) are buried in the structure and barely visible (closed state) **c)** The structural overlay of the open and closed subunits depicts perfect alignment (rmsd = 0.5 Å, 223 C^{α}-atoms). In the closed conformation, Tyr18 is coordinated to the carboxylate group of AZE via Asn139 (H-bonds in black dots). The red arrow marks Thr26 between the open and closed conformations.

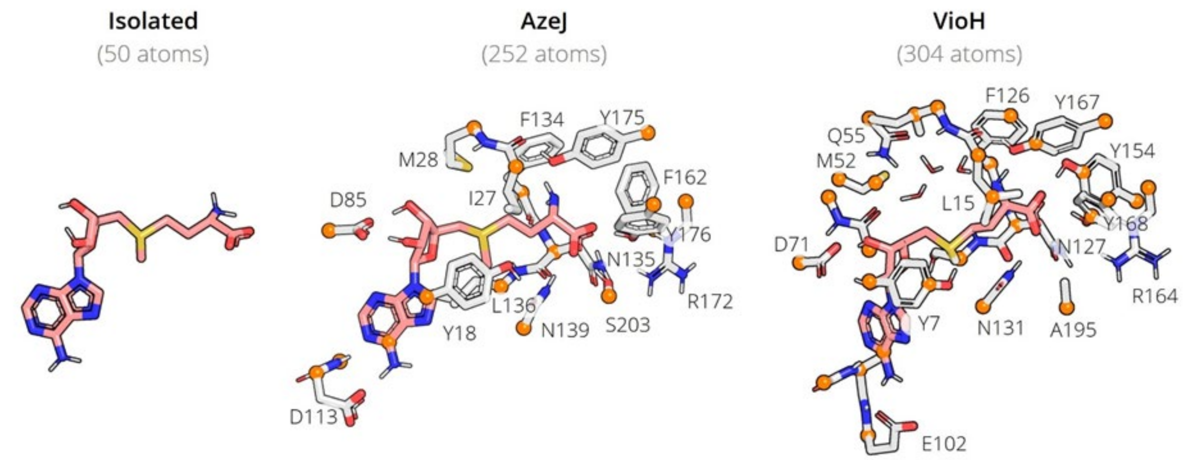

Fig. S12. DFT cluster models of the active site. Reactant states of isolated ligand (*Lig_Wat*), AzeJ (*AzeJ_WT*) and VioH (*VioH_WT*) models after geometry optimisation are shown. SAM is highlighted in pink. Only polar hydrogens are shown. Atoms fixed during geometry and reaction pathway optimisations are marked by orange spheres. Coordinates for reactant and product states of all systems, including the respective fixed atoms, are given in Supplementary Data 1.

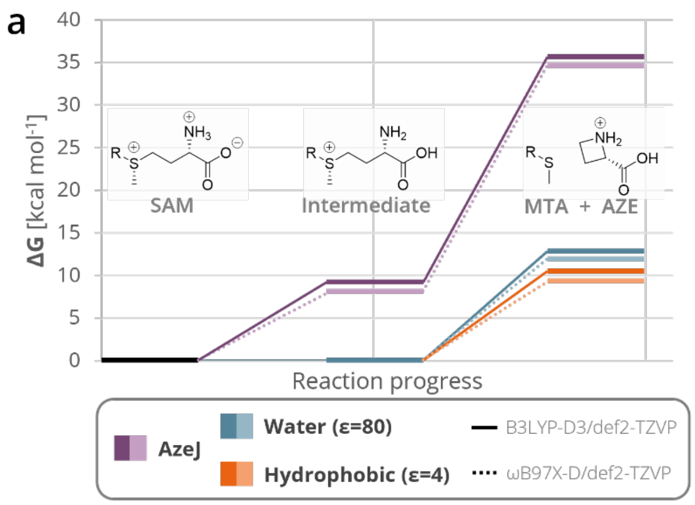

b

Models<br>ΔG [kcal mol-1]	ε=80<br>B3LYP-D3	ε=80<br>ωB97X-D	ε=4<br>B3LYP-D3	ε=4<br>ωB97X-D	AzeJ<br>B3LYP-D3	AzeJ<br>ωB97X-D
**SAM**	0.0	0.0	-	-	0.0	0.0
TS1 (pT)	1.8	1.8	-	-	-	-
**Intermediate**	0.2	0.0	0.0	0.0	8.8	8.0
TS2 (S_N2)	26.8	29.8	29.3	34.2	-	-
**MTA+AZE**	12.7	11.7	10.3	9.2	35.6	34.6

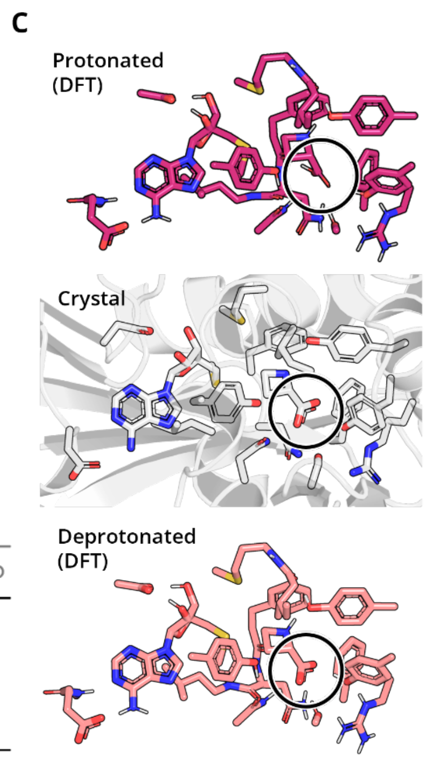

Fig. S13. DFT calculations exclude the formation of AZE-COOH. a) Free energies of reactant (SAM), intermediate (-COOH) after proton transfer (pT), and products (MTA + AZE; see insets) in AzeJ (*AzeJ_pWT*), water (*pLig_Wat*), or hydrophobic environment (*pLig_Hyd*), respectively. DFT calculations were performed at the B3LYP-D3/def2-TZVP and ωB97X-D/def2-TZVP level of theory with vibrational and entropic corrections at the B3LYP-D3-RI/def2-SVP level. In the hydrophobic environment, spontaneous pT occurred during geometry optimisation and HCY-COOH thus served as reactant state. **b)** Numerical values corresponding to a). **c**) Optimised product structures, modelled with protonated (*AzeJ_pWT*) or deprotonated carboxylate (*AzeJ_WT*), in comparison with the crystal structure (PDB ID: 8RYE).

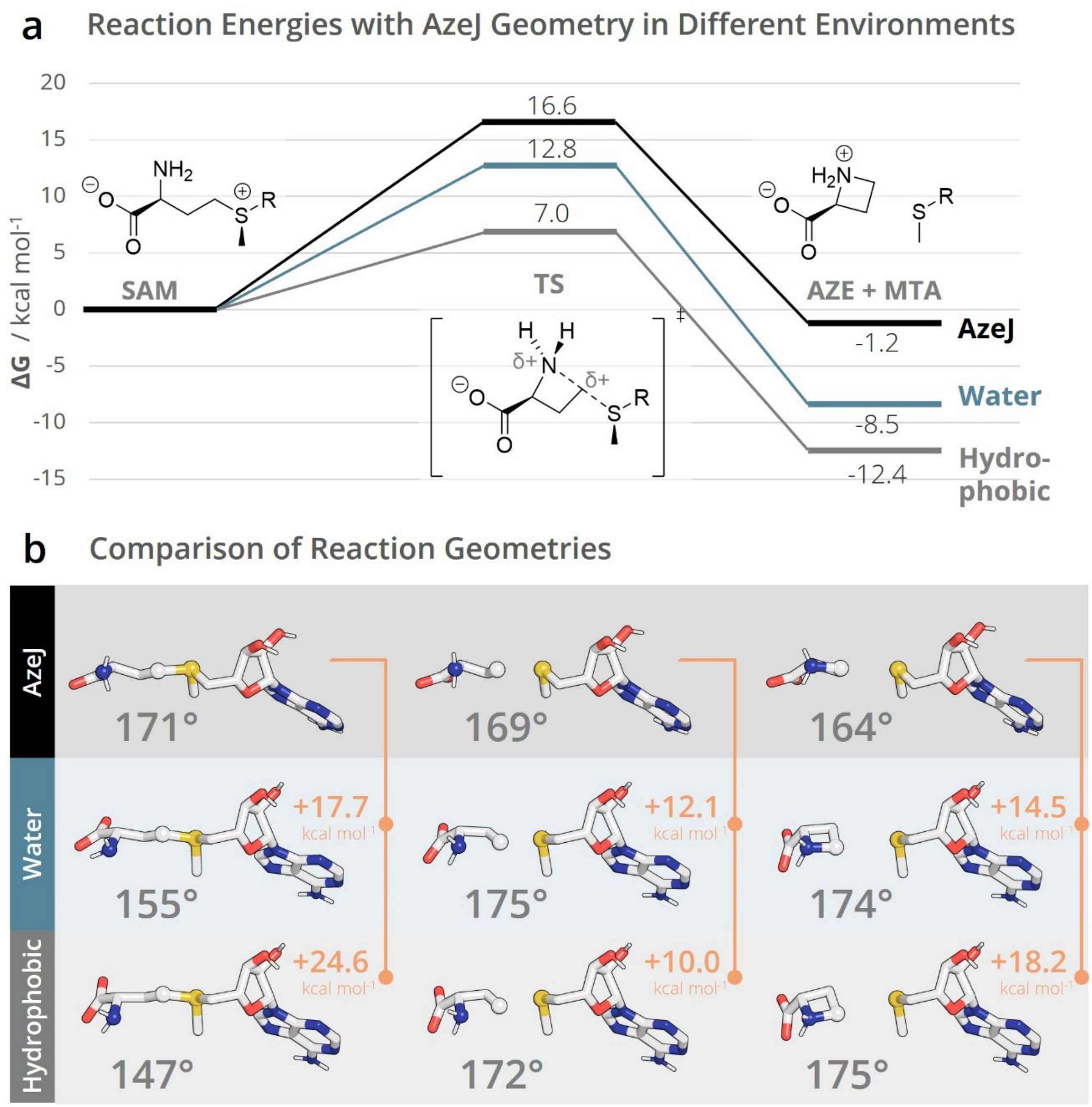

Fig. S14. Analyses of strain effects in AzeJ. a) Free energies of AzeJ-optimised geometries (b, top row) were calculated in AzeJ (*AzeJ_WT*), water (ε=80), or hydrophobic environment (ε=4) at the B3LYP-D3/def2-TZVP level of theory with vibrational and entropic corrections at the B3LYP-D3-RI/def2-SVP level. Energies are given relative to the reactant state. **b)** Geometries of SAM, the transition state (TS), and AZE + MTA optimised in different environments (*AzeJ_WT*, *Lig_Wat*, *Lig_Hyd*). Spheres highlight the reacting N-, C^{γ}-, and S-atoms, with corresponding angles shown below. Orange values represent strain energies of AzeJ in both media, respectively.

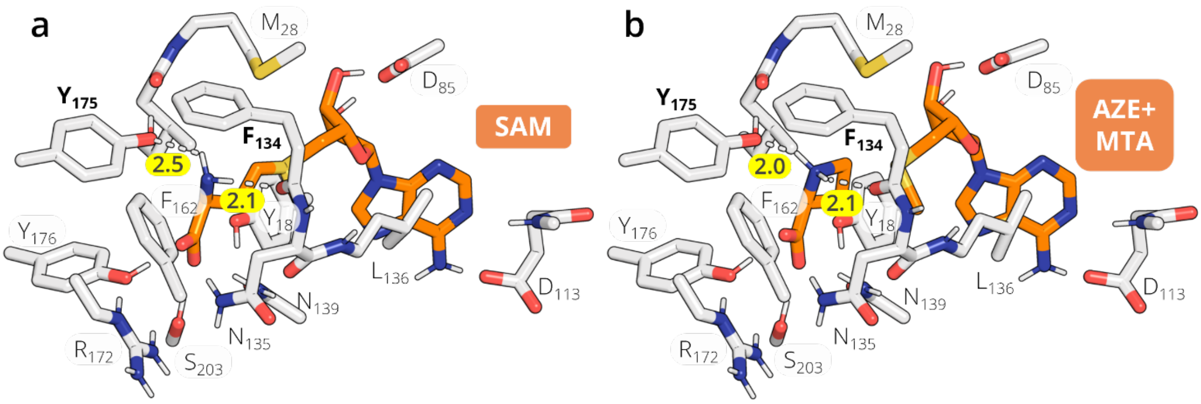

Fig. S15. Hydrogen-bonding to HCY-amine in DFT-optimised models. Geometry-optimised *AzeJ_WT* active-site models of reactant SAM (a) and product AZE+MTA (b). For clarity, only polar hydrogens are shown (distances in Å).

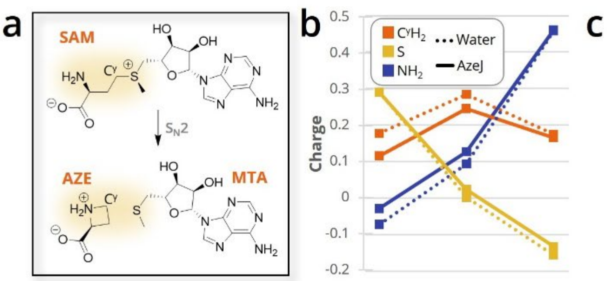

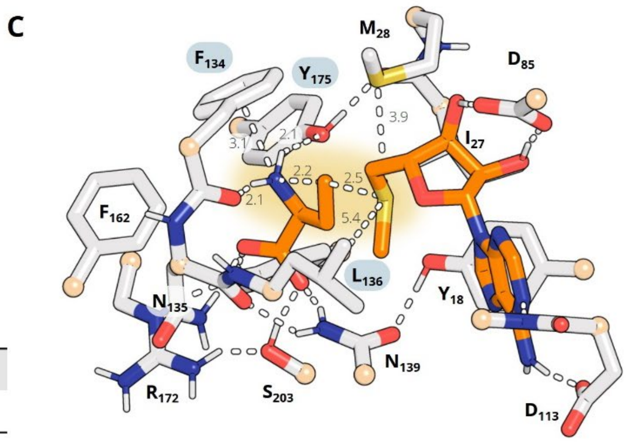

d

ΔG [kcal mol⁻¹]	WT	L136D	Y175F	F134L
TS	16.6	18.8	18.7	17.2
AZE + MTA	-1.2	3.9	3.5	2.2

Fig. S16. Charge transfer and *in silico* mutants. a) Charge transfer from SAM to AZE. **b)** Charge distribution on selected groups involved in the S_N2-reaction (SAM, transition state (TS), AZE + MTA) in AzeJ and water (*AzeJ_WT* ligand geometry evaluated in water (ε=80)). **c)** Alternative view of the AzeJ wild-type (WT) transition state shown in Fig. 3c. Residues highlighted in blue were subjected to *in silico* mutagenesis. Only polar hydrogens are shown for clarity, beige spheres indicate fixed atoms. Distances are given in ångströms. **d)** Free energies of transition states (TS) and products (AZE + MTA) for AzeJ (WT and mutants) were derived from DFT calculations at the B3LYP-D3/def2-TZVP/ε=4 level, with vibrational and entropic corrections at the B3LYP-D3-RI/def2-SVP/ε=4 level, and are given relative to the reactant state.

a **c**

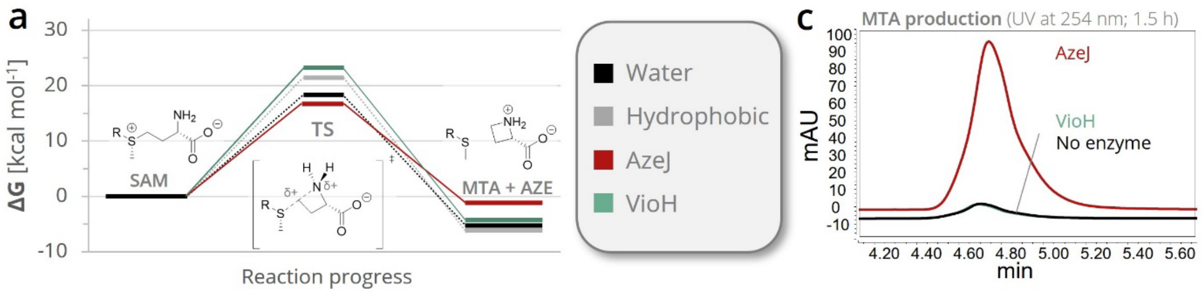

b

**Models**	**ε=80**		**ε=4**		**AzeJ**			**VioH**	
ΔG [kcal mol^{-1}]	B3LYP-D3	ωB97X-D	B3LYP-D3	ωB97X-D	B3LYP-D3	B3LYP-D3*	ωB97X-D	B3LYP-D3	ωB97X-D
TS	**18.4**	22.9	**21.5**	26.3	**16.6**	16.9	20.9	**23.3**	28.3
MTA+AZE	-5.3	-6.5	-6.1	-7.4	-1.2	-1.1	-2.9	-4.4	-6.0

*def2-QZVP

Fig. S17. Selective catalysis: AzeJ promotes AZE formation in contrast to VioH. a) DFT calculations were performed to model the conversion of SAM to AZE+MTA, either in the enzyme environment (*AzeJ_WT* or *VioH_WT*, solid lines) or in solvent (water (ε=80, *Lig_Wat*) or hydrophobic medium (ε=4, *Lig_Hyd*), dashed lines). Electronic energies were calculated at the B3LYP-D3/def2-TZVP level of theory, with vibrational and entropic corrections at the B3LYP-D3-RI/def2-SVP level. Reactant (SAM), products (MTA + AZE) and transition state (TS) are shown as insets. **b)** Numeric values for free energies shown in a) with additional benchmarking at the ωB97X-D/def2-TZVP and B3LYP-D3/def2-QZVP level. Strikingly, the calculated barrier in VioH (23.3 kcal mol^{-1}) exceeds that of the uncatalyzed reaction to form AZE in water (18.4 kcal mol^{-1}). **c)** Activity tests confirm that MTA is only formed via AzeJ.

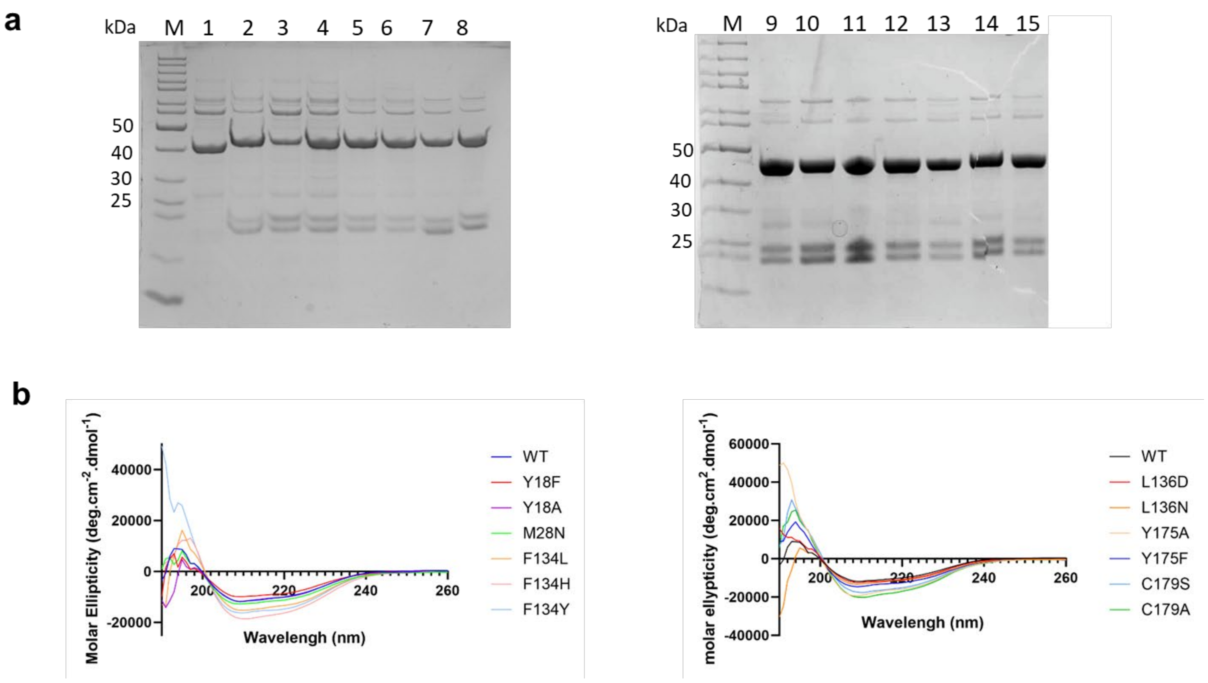

Fig. S18. Proteins used for activity assays. a) SDS-PAGE of proteins from small-scale purification. Lane 1-15: VioH, Y18A, L136D, F134L, AzeJ, L136N, Y18F, M28N, F134H, F134Y, Y175F, Y175A, C179A, C179S, AzeJ. **b)** Circular dichroism spectra showing secondary structures of AzeJ and variants. The spectra were recorded with 5 µM enzyme in 50 mM Tris-HCl (pH 8.0) by a Jasco J-810 spectropolarimeter. Source Data are provided in the Source Data file.

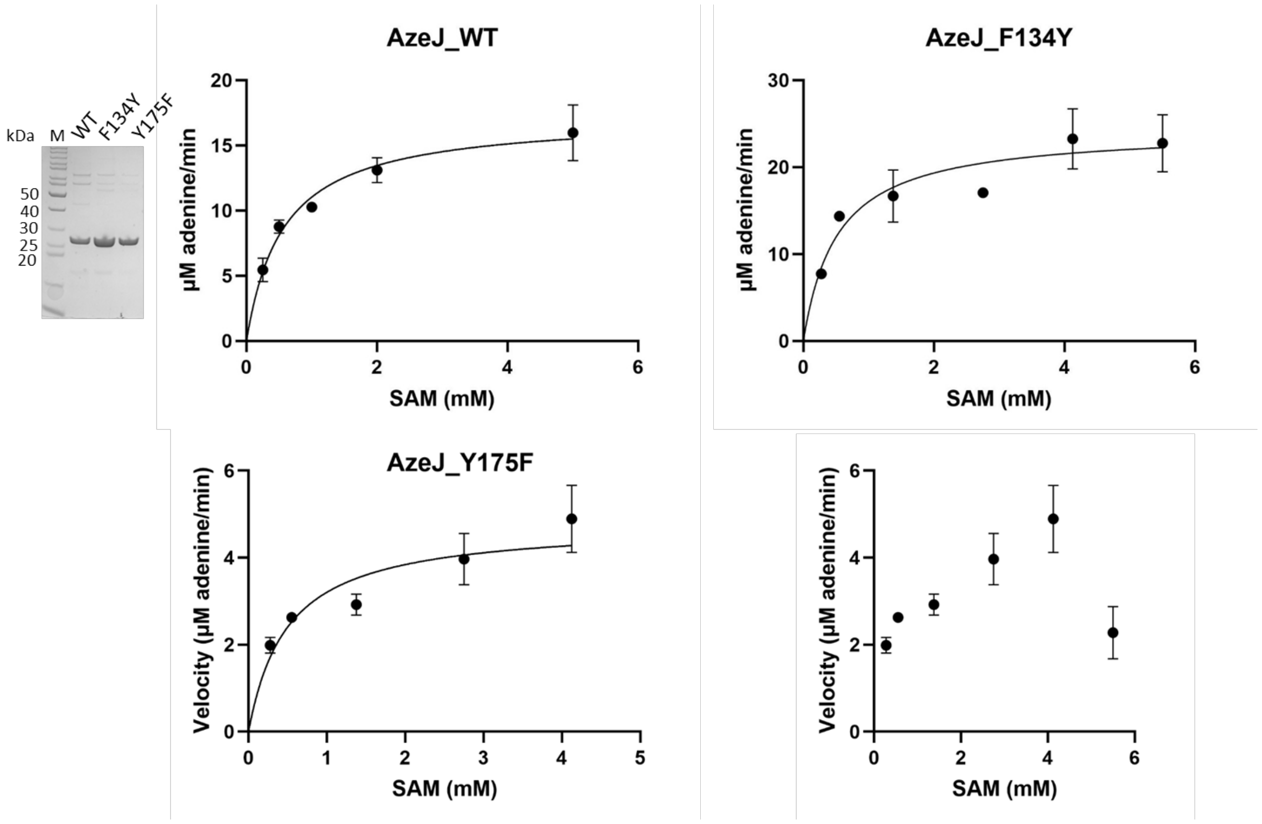

Fig. S19. Michaelis-Menten curves of AzeJ wild-type, F134Y and Y175F variants. The plotted data illustrate the velocity of adenine production (µM/min) in relation to the substrate concentration of SAM (mM). The graphs were generated with GraphPad using the Michaelis-Menten model of the non-linear regression analysis function. Each data point was acquired in triplicates; the error bars represent the standard deviation. Curve fitting R^2 values were 0.92 for WT, 0.80 for F134Y and 0.75 for Y175F. To note, Y175F displayed substrate inhibition at higher concentration of SAM, hence curve fitting was performed by omitting the last data point at 5.25 mM SAM. The raw data is shown beside the Y175F kinetic curve. All kinetics measurements were repeated at least twice. Source Data are provided in the Source Data file.

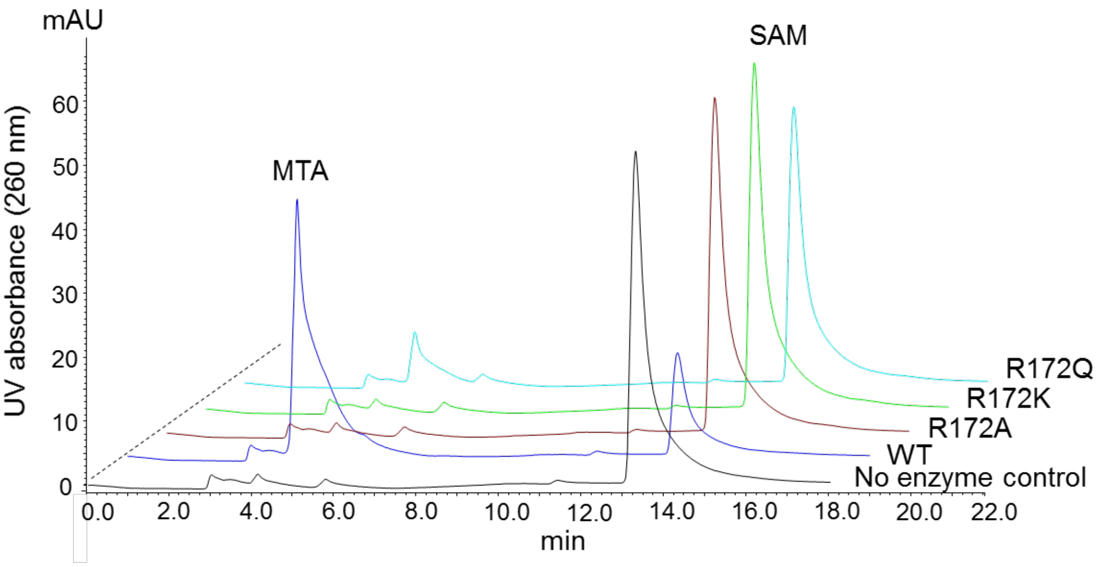

Fig. S20. Activity of AzeJ Arg172 mutants. Reactions in vitro were performed as described in the method section. HPLC traces by UV detection at 260 nm are shown.

```
WP_116614598.1      ENLNGNSP---ISDAPNNDFYAAFEDAVKIMEPGSFSPEVELFLADEKICLTKLFH--VE     81
MDF3054353.1        SILQKAGFVEKYTNKTSYEFYKS-QGKEILV-QENYPLLIKEYLQYESQQVSKIITA-HQ     124
CCG86095.1*         -YMTQDMDIGRSMNQDTPVFYQQDSGAKSIMTLANFPDDMLPLLQMENIATYEAMG--HF     60
VTQ03862.1          -EVSQNMDLTIGT-HRESAFYELEFGPRTIMTLANFPDDVLPLLQMESLMTFEAMA--YL     75
AzeJ                --MSQNMDLTIGT-HRESAFYELEFGPRTIMTLANFPDDVLPLLQMESLMTFEAMA--YL     55
GFJ87888.1          -PDRGGSPVAVSTDQRVASFFD--TAWRRIIA--TQPASVRVHLAAERRLT----ESVLP     152
MBX9695614.1*       --VKR----ANLRLHEIGQFYGERKGWRMFDSH-PLTPELKNYLSAEAEFLGTVMQD--G     70
MCC6746182.1        --VPTGEVVWTCFDKEVAQFYGEEGGARMFDDV-RNTPEVENYLNEEAVALDRLFRAL-P     70
VioH                --------------MSTLDFYAQGQGDRLIDPA-RFPAEIKAFLEGERVLL----DSVAE     41
MBP2169418.1        -------MVNNKNAVTVETFYCYESGWRMFDVS-RLTSAVNAYLCAEKAIIANLLQQPEA     52
WAT00603.1          -----MTNDENRCTDRVETFYGQHNGWRMFDGR-MLTPEVAAYLVEEKRLVAEYLEEAGE     54
KAB7894704.1        -----MLVCDEIAEDNVAKFYGQNNGWRMFDGR-ILTAEVAAFLVEESRCIANYLENSAN     54
EIC83926.1          -----MLTYDVAAKDRVEMFYGQKNGWRMFDGR-MLTPEVTAYLNEEKRQIAGYLENSAS     54
                                       *:        :         :   *  *

WP_116614598.1      QYDCIIEVGCHSGRN-AGWLSSLCHRYIGIDINESAIRSAKNAHASNARIE--FVCG---     135
MDF3054353.1        SYTALLEVACADARN-MDIALKNNLKYYGIDFIRENIDKAEKRIKALNQISTAFVECASI     183
CCG86095.1*         RCNTLVELGCYDGRA-LEIARLLNARYQGIDLNPRAIKVLRERVKQEE---------IED     110
VTQ03862.1          RCDALVELGCYDGRA-LEIARLLNARYLGVDLDQRAIETLRTRIEREG---------MSD     125
AzeJ                RCDALVELGCYDGRA-LEIARLLNARYLGVDLDQRAIETLRTRIEREG---------MSD     105
GFJ87888.1          AYRAMVEVGCADGSLLLPVARRCALDYLGLDLAAGAVAATRAAGADA-------------     199
MBX9695614.1*       SYKTLIEVGCGYGRY-LDWALSRGYHYVGMDIVQWLVDLGQVRTARAKKKFPHLSCGVIR     129
MCC6746182.1        DRNHLIEVGCGHGRY-RPWAAERDLNYDGLDLVAALVDAGRKLHVPTR---PDLRSKLHI     126
VioH                HVELLVEVGSMHGQH-LGWAIARGKHYIGVDPVPRYIEQGRRTLREQG-LPAERFRFI-E     98
MBP2169418.1        QIDEFIEVGCGYGRY-LPLISAQNINYCGIDLVSWLVALGKIRLPLYQ-AHSAGQARIEV     110
WAT00603.1          ALEQFIEVGCGYGRY-LHVALKHGVSYRGIELVGWLAALGKARVTACP-LPPNCSAQIEH     112
KAB7894704.1        TLNQFVEIGCGYGRY-LHVALKHKVSYRGVELVQWLAELGKARINACP-LPKNCSAQIEH     112
EIC83926.1          GLSQFVEIGCGYGRY-LHVALKYRVNYRGVELVRWLAELGKARIAACQ-LPENCSAQIEH     112
                        ::*:..  .             * *::         .

WP_116614598.1      -PVEHVIS--SIL--DENLCKRRIVVLFPFNLFGNFVNVDELISSFSLTGADLAMSNFNT     190
MDF3054353.1        FDLSEKK--------DFIPETEKVLTFFPFNALGNMGSIISVLKIVLNLNYDIVFSIFKT     235
CCG86095.1*         RVDTIVGDVLTHMSRGQLSVGSQTLYLLPFNFLGNFRDPLPLLKKLAKLDVISVICVFSN     170
VTQ03862.1          RADTVVDDILNHTRRG-ASVGSRALYLLPFNLLGNFREPKRLLDSLAERSVAAVVSVFGD     184
AzeJ                RADTVVDDILNHTRRG-ASVGSRALYLLPFNLLGNFREPKRLLDSLAERSVAAVVSVFGD     164
GFJ87888.1          ----VRADVLELT--GLALPAGPLLVAFPFNVFGNLPEPERALGAVAASGADALVLTYDT     253
MBX9695614.1*       HPAEEIDRAVREL--SLDHPEQKALVFFPFNCMGNVAQFDTVVDSLGRSNLDLVVSTFKT     187
MCC6746182.1        ESVEELGRLFEHE--GLQGHGNRVITLFPFNCFGNLARPSMVLRALAHTQASALVSTYST     184
VioH                GGAEELHQLLPRH--ALAVPPSRCLLFFPFNSFGNMRDPERVLESLSMTGLPFLISSYAT     156
MBP2169418.1        CSVDKVRDLLIAS--DQDKTKKFC-IFFPFNCFGNLPYPRQVLAHLSGLNVTMIISGFLP     167
WAT00603.1          LSAEHLHCVLPDS--DIDIAGKSC-LFFPFNCFGNLRDPLRVLAELKGRNTEVIISGFAS     169
KAB7894704.1        LSAEHLHCLFPNP--TQSIAAKSC-VFFPFNCFGNLSSPLRVLGELKGRNSEVIISGFSC     169
EIC83926.1          LSADHLHCVLPYS--EHDISSKSC-VFFPFNCFGNLSDPLRVLSELKGRNTEVIISGFSS     169
                                               :*** :**.      :  .        .  :

WP_116614598.1      RSATTLGRYNYYSNCFPRSQIRVYDAEQGVLFKAGNHF-RSIAYDANYLRKLIRDRSNYH     249
MDF3054353.1        TDEANRTRFEYYQNCGY-SNLKCYDDGNGIIFTSSEGL-KSIAYFEHYLKTLFETLNCRV     293
CCG86095.1*         SPEAIRVRHDYYLRCGV-QALELHSHEDGTLFTGADGF-YSRSYTRSSFHSLLTRCGLSV     228
VTQ03862.1          SAEATRVRQSYYRRCGG-AGPGVAHAGRRHRVHRQRRLLLALVFTR------LPPRPARR     237
AzeJ                SAEATRVRQSYYRRCGV-QGLELHTRDDGTVFTGSDGF-YSRSYSRACLHALLAECGLTV     222
GFJ87888.1          SAGAAAVRSEYYRACGL-AGELVAD-GTGVHFTA-APF-TSSVYHRAVLTGWLAGHGYRV     309
MBX9695614.1*       DATSTKFRKEYYGKCGY-EQLNSRILKQGLLIISEEGF-HAMAYHQDVLVNSFKKRGFDL     245
MCC6746182.1        DPETTNLRITYYEQCHY-SGLVAQDLPKGVVIRSREGL-RAYAYREEYLTRLFAAVNYRL     242
VioH                TERATQARAAYYAQCQY-EWLESACDERGVRFRAPEGF-DAMAYHVEYLEPRMRRYGLEV     214
MBP2169418.1        DARTSDLRQDYYAKCGC-QHLQVSPGEQGTLITSADGL-NSLAYHPESLQQLLAEFGFSL     225
WAT00603.1          NEQATLARMAYYQQCGC-TDLRCRVTERGVEISSREFL-QSLAYDAENLEILLNEFGFRL     227
KAB7894704.1        SDKATQARIAYYARCGC-TGLSSRLRDTGVEISSDESL-QSLAYQPEKLEMLLNEFGFRL     227
EIC83926.1          SEQTTSARMAYYAKCGC-TDLSSQHTERGVQISSSEFL-ESLAYQSGALELLLNEFGFRL     227
                       :   *  **  *                .     :  :  :        :
```

Fig. S21. Proposed sequence signatures of AZE synthases. Multiple sequence alignment of AzeJ, its 10 closest homologs, and VioH by Clustal Omega.[5] For clarity, the central portion of the alignment is shown. Sequence signature blocks are highlighted in yellow. Homologous protein sequences are WP_116614598.1 (*Paraburkholderia unamae*), MDF3054353.1 (γ-Proteobacteria from soil metagenome), CCG86095.1 (*Erwinia piriflorinigrans*), VTQ03862.1 (*Pseudomonas aeruginosa*), GFJ87888.1 (*Phytohabitans rumicis*), MBX9695614.1 (Cyanobacteria bacterium from water metagenome), MCC6746182.1 (Δ-Proteobacteria from water metagenome), MBP2169418.1 (*Erwinia toletana*), WAT00603.1 (*Rouxiella chamberiensis*), KAB7894704.1 (*Rouxiella* sp.), EIC83926.1 (*Serratia* sp.). The sequences of CCG86095.1 and MBX9695614.1 were manually inspected and re-annotated to include additional residues at the N-terminus.

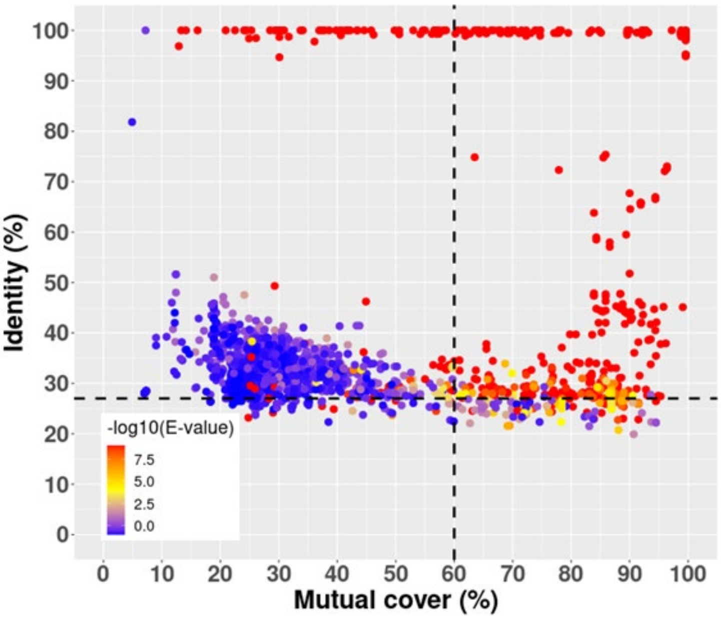

Fig. S22. Distribution of sequence identity and mutual coverage with AzeJ/VioH of non-redundant hits. On the scatterplot, each dot corresponds to the alignment of a hit in the NCBI non-redundant protein sequence database with either VioH or AzeJ. The y axis gives the percentage of amino acid sequence identity of the target sequence with the query, whereas the x axis gives the minimum between the percentage of the query length and of the target length being covered by the alignment. The color of a dot corresponds to the -log10 of the E-value of the alignment, with red values corresponding to the most significant hits. The dashed lines represent the identity and mutual coverage cutoffs used to filter the hits. The cutoffs were defined as such to maximise the amount of reliable hits and to minimise the amount of spurious hits among the pool of retained sequences.

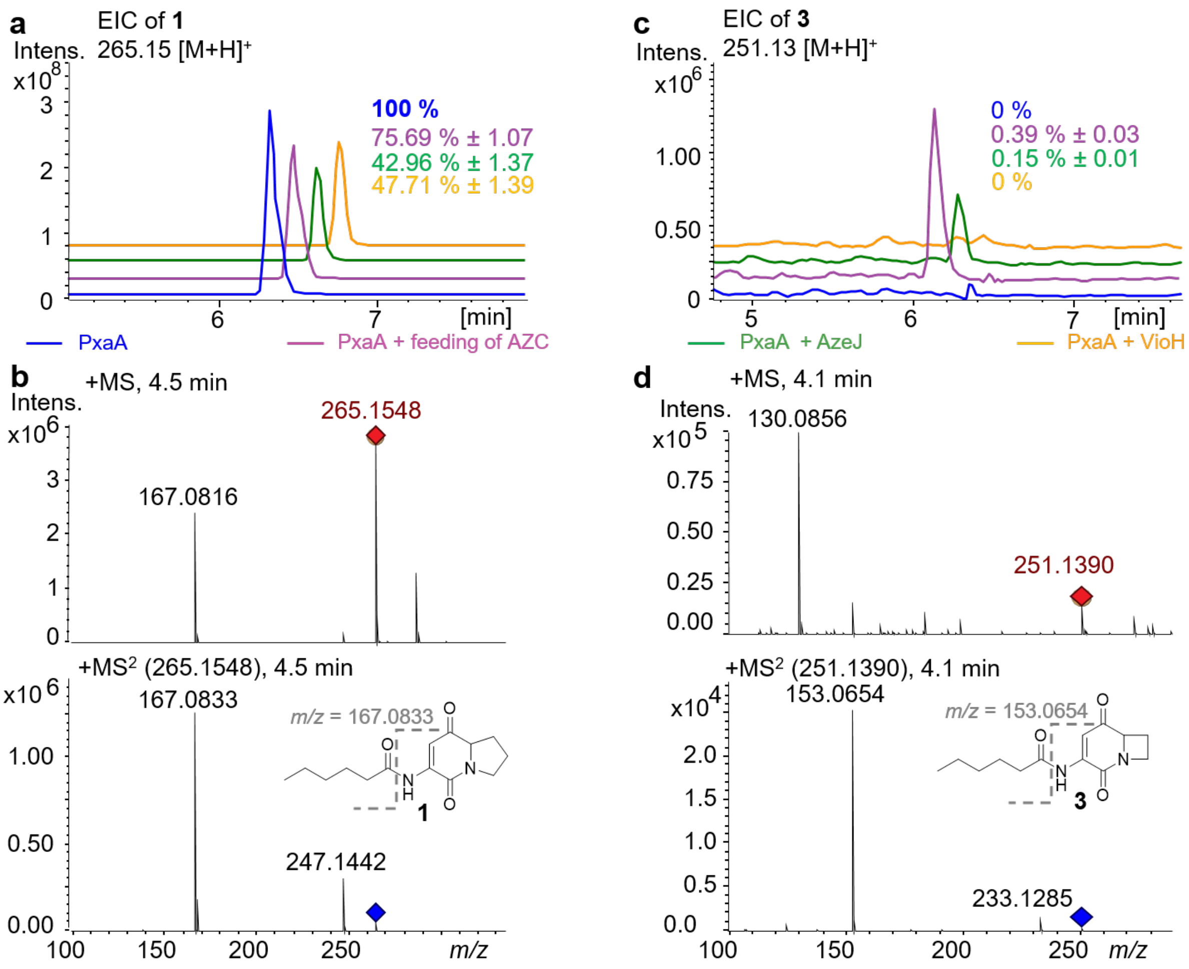

Fig. S23. Production of AZE-incorporated pyrrolizixenamides. Extracted ion chromatograms (EICs) of indicated compounds under various conditions: Expression of *pxaA* in *E. coli* (blue), feeding AZE to a *pxaA*-expressing *E. coli* strain (pink), co-expression of *pxaA* and *azeJ* (green), as well as co-expression of *pxaA* and *vioH* (yellow) in *E. coli*. **a)** EIC of *m/z* 265.15 $[M+H]^+$ used for relative quantification and **b)** LC-HRMS/MS analysis of **1** (*m/z* calc'd for $C_{14}H_{20}N_2O_3$ $[M+H]^+$ = 265.1546; obs'd. *m/z* = 265.1548; Δ ppm = 0.3). Inset: proposed structure of **1** indicating the key MS^2 fragment (*m/z* calc'd for $C_8H_{10}N_2O_2$ $[M+H]^+$ = 167.0815). **c)** EIC of *m/z* 251.13 $[M+H]^+$ used for relative quantification and **d)** LC-HRMS/MS analysis of **3** (*m/z* calc'd for $C_{13}H_{18}N_2O_3$ $[M+H]^+$ = 251.1390; obs'd. *m/z* = 251.1390; Δ ppm = 0). Inset: proposed structure of **3** indicating the key MS^2 fragment (*m/z* calc'd for $C_7H_8N_2O_2$ $[M+H]^+$ = 153.0658). Relative quantification of peptides was performed by setting the production of **1** by the wild-type PxaA to 100 % (see panel a). Relative quantities of **1** and **3** are given with standard deviations.

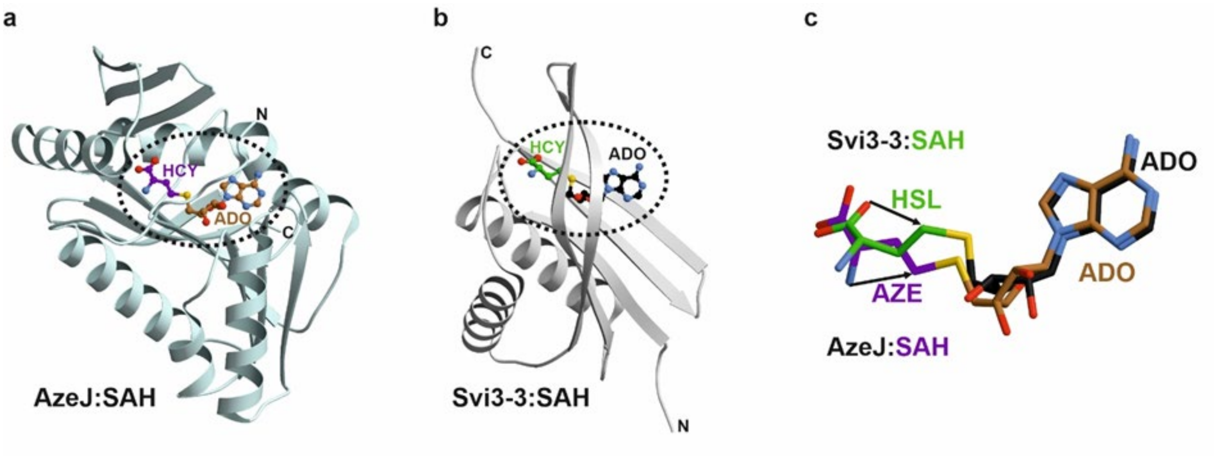

Fig. S24. Methionine residue arrangement in SAM determines cyclisation reaction. While Svi3-3 was shown to catalyse the cleavage of SAM into MTA and *L*-homoserine lactone (HSL),[6] AzeJ forms MTA and AZE. **a), b)** Coiled representations of AzeJ (PDB ID: 8RYD, C-atoms in cyan) and Svi3-3 (PDB ID: 6ZMG, C-atoms in white) in complex with SAH. Due to distinct protein folding, the structures were aligned via the adenine residue of SAH. **c)** Molecular insights into the catalytic centres of each lyase illustrate characteristic arrangements of the homocysteine residue (HCY). In 6ZMG, the carboxylate group of HCY (C-atoms in green) is positioned for nucleophilic attack on the C^{γ}-atom (distance 3.4 Å) to form MTA and HSL. In contrast, the free amine group of the HCY residue in AzeJ (C-atoms in purple) is perfectly aligned to form MTA and AZE (distance 2.9 Å). These distinctive conformations prevent side reactions and allow the enzymes to selectively cleave SAM to either HSL or AZE (indicated by the black arrow).

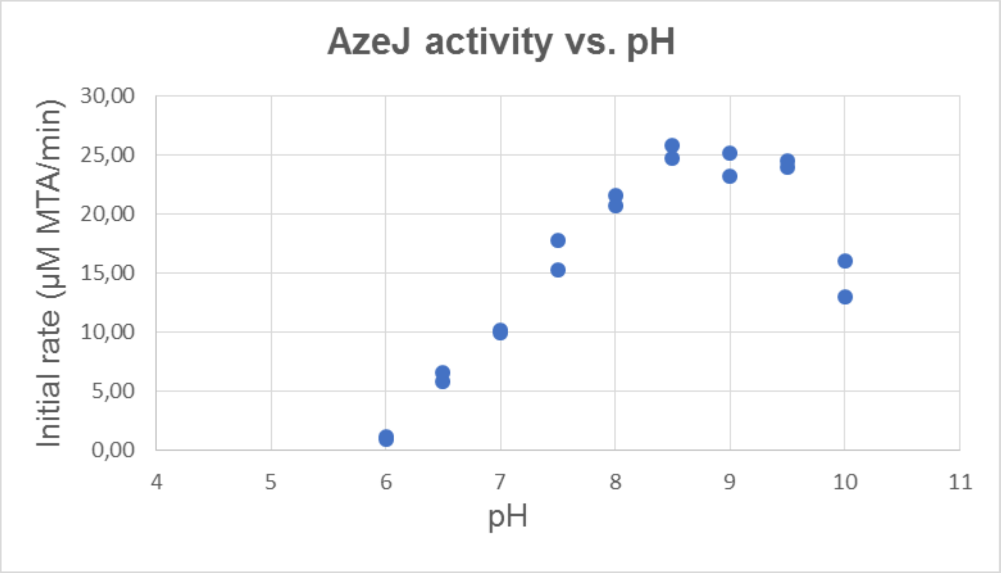

Fig. S25. AzeJ activity vs. pH. Activity assays were performed with 1 µM AzeJ and 1 mM SAM at 30 °C for 9 mins. Produced MTA was analysed and quantified by HPLC. Each measurement was acquired in duplicates and all data points were plotted. Source Data are provided in the Source Data file.

REFERENCES

1. Kegler, C. & Bode, H. B. Artificial splitting of a non-ribosomal peptide synthetase by inserting natural docking domains. *Angew. Chem. Int. Ed.* **59**, 13463–13467 (2020).
2. Bozhüyük, K. A. J. *et al.* Evolution inspired engineering of megasynthetases. *Science* **383**, eadg4320 (2024).
3. Fenwick, M. K., Almabruk, K. H., Ealick, S. E., Begley, T. P. & Philmus, B. Biochemical characterization and structural basis of reactivity and regioselectivity differences between *Burkholderia thailandensis* and *Burkholderia glumae* 1,6-didesmethyltoxoflavin *N* -methyltransferase. *Biochemistry* **56**, 3934–3944 (2017).
4. Holm, L. & Rosenström, P. Dali server: conservation mapping in 3D. *Nucleic Acids Res.* **38**, W545–W549 (2010).
5. Sievers, F. *et al.* Fast, scalable generation of high-quality protein multiple sequence alignments using Clustal Omega. *Mol. Syst. Biol.* **7**, 539 (2011).
6. Guo, X. *et al.* Structure and mechanism of a phage-encoded SAM lyase revises catalytic function of enzyme family. *eLife* **10**, e61818 (2021).
