# Supplementary material for: Molecular basis for azetidine-2-carboxylic acid biosynthesis
Source: Nat Commun. 2025 Feb 4;16:1348. doi: 10.1038/s41467-025-56610-6 (PMC11794875; doi:10.1038/s41467-025-56610-6)
Supplement: Supplementary file 2 — Description of Additional Supplementary Files [file 41467_2025_56610_MOESM2_ESM.pdf]

Description of Additional Supplementary Files

Supplementary Data 1. Coordinates of the DFT optimised QM cluster models (abbreviations as introduced in Table S5). Atoms fixed to the experimentally determined positions are marked with an f.

Supplementary Data 2. AzeJ homologues used in phylogenetic analysis.
