# Supplementary material for: Molecular basis for azetidine-2-carboxylic acid biosynthesis
Source: Nat Commun. 2025 Feb 4;16:1348. doi: 10.1038/s41467-025-56610-6 (PMC11794875; doi:10.1038/s41467-025-56610-6)
Supplement: Supplementary file 3 — Supplementary Data 1 [file 41467_2025_56610_MOESM3_ESM.docx]

**Supplementary Data 1.** Coordinates of the DFT optimised QM cluster models (abbreviations as introduced in Table S5). Atoms fixed to the experimentally determined positions are marked with an f.

| ***AzeJ_WT*** Reactant | ***AzeJ_WT*** Product | Fixed (f) |
| --- | --- | --- |

| C | 48.8200000 | 9.6360000 | 11.4410000 | C | 48.8200000 | 9.6360000 | 11.4410000 | f |
| --- | --- | --- | --- | --- | --- | --- | --- | --- |
| C | 47.3360496 | 9.6016843 | 11.7360636 | C | 47.3301949 | 9.6163835 | 11.7053218 |  |
| C | 46.7554014 | 10.5048845 | 12.6435675 | C | 46.7379957 | 10.5354212 | 12.5903999 |  |
| C | 46.4866193 | 8.6574092 | 11.1366019 | C | 46.4901977 | 8.6596817 | 11.1136996 |  |
| C | 45.3876757 | 10.4746585 | 12.9330429 | C | 45.3695466 | 10.5033691 | 12.8717992 |  |
| C | 45.1187233 | 8.6024334 | 11.4249701 | C | 45.1195893 | 8.6073773 | 11.3902612 |  |
| C | 44.5539193 | 9.5214273 | 12.3258920 | C | 44.5451397 | 9.5364326 | 12.2739288 |  |
| O | 43.2284155 | 9.5201111 | 12.6429141 | O | 43.2207854 | 9.5254074 | 12.5886226 |  |
| C | 42.1220000 | 15.0210000 | 14.3510000 | C | 42.1220000 | 15.0210000 | 14.3510000 | f |
| C | 41.9709336 | 15.0510267 | 15.8826581 | C | 41.9709031 | 15.0003261 | 15.8820688 |  |
| O | 40.9540937 | 14.6134887 | 16.4414908 | O | 40.9775628 | 14.4925252 | 16.4315896 |  |
| C | 42.8279328 | 13.7169648 | 13.8864776 | C | 42.8253571 | 13.7378190 | 13.8329023 |  |
| C | 42.8801303 | 13.6216178 | 12.3506873 | C | 42.9307339 | 13.7372042 | 12.2965937 |  |
| C | 42.1678791 | 12.4636259 | 14.4780407 | C | 42.1284002 | 12.4620809 | 14.3227877 |  |
| C | 43.6807658 | 14.7244598 | 11.6562612 | C | 43.7514408 | 14.8804321 | 11.6973677 |  |
| N | 43.0271092 | 15.5079895 | 16.6002645 | N | 42.9978828 | 15.5080785 | 16.6030797 |  |
| C | 43.1990000 | 15.3970000 | 18.0630000 | C | 43.1990000 | 15.3970000 | 18.0630000 | f |
| C | 44.5291271 | 14.7136941 | 18.3969882 | C | 44.5309177 | 14.7075473 | 18.3715334 |  |
| C | 44.6727122 | 13.3098692 | 17.8050868 | C | 44.6426711 | 13.2898985 | 17.8074992 |  |
| S | 43.4294695 | 12.1152845 | 18.4325149 | S | 43.4662578 | 12.0883833 | 18.5442703 |  |
| C | 44.2440193 | 11.6220576 | 19.9942018 | C | 44.4140664 | 11.6246355 | 20.0378799 |  |
| C | 51.6880000 | 9.6280000 | 19.1070000 | C | 51.6880000 | 9.6280000 | 19.1070000 | f |
| C | 50.3466089 | 9.8574035 | 18.3812947 | C | 50.3928325 | 10.0912714 | 18.4344830 |  |
| O | 49.2790884 | 9.5737903 | 19.0026762 | O | 49.3120373 | 9.8654552 | 19.0556292 |  |
| O | 50.4026566 | 10.2925994 | 17.2035731 | O | 50.4876948 | 10.6215176 | 17.2998100 |  |
| C | 39.1800000 | 7.5290000 | 20.5670000 | C | 39.1800000 | 7.5290000 | 20.5670000 | f |
| C | 39.1638756 | 6.8706443 | 19.1739981 | C | 39.0065732 | 6.9396490 | 19.1525830 |  |
| O | 39.7256951 | 7.3819722 | 18.2084920 | O | 39.2915802 | 7.5813060 | 18.1414083 |  |
| C | 39.9329409 | 8.8573167 | 20.6139605 | C | 39.8896263 | 8.8828118 | 20.5661175 |  |
| C | 39.2755505 | 10.0637600 | 19.9648719 | C | 39.1742356 | 10.0495412 | 19.9065323 |  |
| C | 37.8904043 | 10.1774502 | 19.7784083 | C | 37.7980556 | 10.0515337 | 19.6290727 |  |
| C | 40.0834844 | 11.1446685 | 19.5685510 | C | 39.9166693 | 11.1971188 | 19.5712623 |  |
| C | 37.3327228 | 11.3358616 | 19.2229605 | C | 37.1829006 | 11.1674978 | 19.0464220 |  |
| C | 39.5356760 | 12.2997611 | 19.0061899 | C | 39.3097283 | 12.3125248 | 18.9876577 |  |
| C | 38.1487681 | 12.4014110 | 18.8372314 | C | 37.9323123 | 12.3045581 | 18.7297521 |  |
| N | 38.5270357 | 5.6613457 | 19.0682303 | N | 38.5357146 | 5.6601541 | 19.0653712 |  |
| C | 38.2620000 | 4.9980000 | 17.7770000 | C | 38.2620000 | 4.9980000 | 17.7770000 | f |
| C | 39.5695328 | 4.8944505 | 16.9235760 | C | 39.5613720 | 4.9179320 | 16.9061059 |  |
| O | 39.5670289 | 5.1816104 | 15.7289078 | O | 39.5209441 | 5.1379735 | 15.6981149 |  |
| C | 37.1887348 | 5.7417870 | 16.9704325 | C | 37.1409482 | 5.7091372 | 17.0049191 |  |
| C | 36.8435852 | 5.0097005 | 15.6754242 | C | 36.7924779 | 5.0157496 | 15.6912277 |  |
| N | 36.6789241 | 5.8008192 | 14.5927513 | N | 36.6003515 | 5.8531156 | 14.6410111 |  |
| O | 36.6822232 | 3.7910661 | 15.6621443 | O | 36.6351653 | 3.8013477 | 15.6286272 |  |
| N | 40.6623407 | 4.4269756 | 17.5655353 | N | 40.6807358 | 4.5391729 | 17.5615071 |  |
| C | 41.9510000 | 4.2010000 | 16.9160000 | C | 41.9510000 | 4.2010000 | 16.9160000 | f |
| C | 43.1286192 | 4.6212480 | 17.8015808 | C | 43.1679850 | 4.7062043 | 17.6945189 |  |
| C | 44.4862112 | 4.0158167 | 17.3920272 | C | 44.4790592 | 3.9647703 | 17.3566157 |  |
| C | 44.9261054 | 4.3832707 | 15.9673737 | C | 44.7769747 | 3.8845359 | 15.8518113 |  |
| C | 45.5797151 | 4.4117038 | 18.3938443 | C | 45.6631428 | 4.5888361 | 18.1083903 |  |
| C | 32.5930000 | 5.8730000 | 16.5430000 | C | 32.5930000 | 5.8730000 | 16.5430000 | f |
| C | 33.0410506 | 7.2945957 | 16.8108366 | C | 32.9414594 | 7.3136139 | 16.8446105 |  |
| C | 32.2394935 | 8.1955504 | 17.5308809 | C | 32.0626334 | 8.1478787 | 17.5550545 |  |
| C | 34.2943745 | 7.7419173 | 16.3561268 | C | 34.1719881 | 7.8501086 | 16.4260531 |  |
| C | 32.6825907 | 9.4991199 | 17.7932361 | C | 32.4041932 | 9.4756246 | 17.8429942 |  |
| C | 34.7553975 | 9.0342195 | 16.6303030 | C | 34.5235891 | 9.1718519 | 16.7180577 |  |
| C | 33.9413008 | 9.9201638 | 17.3479315 | C | 33.6362555 | 9.9918530 | 17.4261729 |  |
| C | 31.6810000 | 9.4790000 | 14.1040000 | C | 31.6810000 | 9.4790000 | 14.1040000 | f |
| C | 31.7208823 | 8.0208886 | 13.6616143 | C | 31.7007276 | 8.0239052 | 13.6550689 |  |
| N | 33.0513352 | 7.6585439 | 13.1779481 | N | 33.0252378 | 7.6392911 | 13.1676124 |  |
| C | 33.3523211 | 6.5059157 | 12.5836589 | C | 33.2910163 | 6.4774446 | 12.5702016 |  |
| N | 32.4220945 | 5.5345547 | 12.4890702 | N | 32.3367385 | 5.5295720 | 12.4948278 |  |
| N | 34.5624361 | 6.3018413 | 12.0526488 | N | 34.4856973 | 6.2416634 | 12.0222848 |  |
| C | 33.8900000 | 14.8690000 | 14.5720000 | C | 33.8900000 | 14.8690000 | 14.5720000 | f |
| C | 35.2708172 | 14.3362123 | 14.9000112 | C | 35.2651396 | 14.3316465 | 14.9140334 |  |
| C | 36.3511950 | 15.1969082 | 15.1440140 | C | 36.3554879 | 15.1828414 | 15.1536735 |  |
| C | 35.5234116 | 12.9513956 | 14.9698901 | C | 35.5035350 | 12.9467331 | 14.9844858 |  |
| C | 37.6325292 | 14.7099114 | 15.4340429 | C | 37.6335843 | 14.6862274 | 15.4427337 |  |
| C | 36.7894932 | 12.4453615 | 15.2655691 | C | 36.7677538 | 12.4334492 | 15.2734510 |  |
| C | 37.8650063 | 13.3258890 | 15.4879549 | C | 37.8494355 | 13.3018281 | 15.4960309 |  |
| O | 39.0870379 | 12.8030710 | 15.7435696 | O | 39.0686378 | 12.7591664 | 15.7626744 |  |
| C | 35.5190000 | 13.7510000 | 9.6800000 | C | 35.5190000 | 13.7510000 | 9.6800000 | f |
| C | 35.5025269 | 12.5002056 | 10.5245774 | C | 35.5084723 | 12.5159522 | 10.5556352 |  |
| C | 36.6488826 | 12.0617009 | 11.2079386 | C | 36.6246574 | 12.1498288 | 11.3263155 |  |
| C | 34.3291578 | 11.7467213 | 10.6807606 | C | 34.3685978 | 11.7012535 | 10.6525785 |  |
| C | 36.6299356 | 10.9389234 | 12.0386296 | C | 36.6039661 | 11.0418866 | 12.1789735 |  |
| C | 34.2913099 | 10.6237687 | 11.5082178 | C | 34.3301738 | 10.5938290 | 11.5027920 |  |
| C | 35.4352773 | 10.2245116 | 12.2159063 | C | 35.4409838 | 10.2682010 | 12.2950393 |  |
| O | 35.3317062 | 9.1538125 | 13.0444076 | O | 35.3317023 | 9.2037348 | 13.1436052 |  |
| C | 37.8850000 | 7.0690000 | 10.5480000 | C | 37.8850000 | 7.0690000 | 10.5480000 | f |
| O | 37.2543701 | 7.0151469 | 11.8119111 | O | 37.1319044 | 6.9398816 | 11.7432134 |  |
| C | 42.4750000 | 4.8880000 | 12.1800000 | C | 42.4750000 | 4.8880000 | 12.1800000 | f |
| C | 41.7735494 | 6.2223878 | 12.3225767 | C | 41.7724928 | 6.2224199 | 12.3318732 |  |
| N | 40.6801995 | 6.2837219 | 13.0913162 | N | 40.7139569 | 6.2867719 | 13.1535478 |  |
| O | 42.2220763 | 7.2188091 | 11.7267297 | O | 42.1742902 | 7.2082770 | 11.6919626 |  |
| C | 54.3690000 | 3.4430000 | 14.4320000 | C | 54.3690000 | 3.4430000 | 14.4320000 | f |
| C | 53.9017827 | 2.5104775 | 15.5834750 | C | 53.9073402 | 2.5247263 | 15.5979153 |  |
| O | 54.7132420 | 1.7840621 | 16.1630082 | O | 54.7265359 | 1.8306396 | 16.2064663 |  |
| C | 54.6580806 | 2.6179281 | 13.1667463 | C | 54.6141902 | 2.6060560 | 13.1643275 |  |
| C | 53.3432048 | 2.1263778 | 12.5132378 | C | 53.2798405 | 2.1029039 | 12.5571986 |  |
| O | 52.5599508 | 3.0647969 | 12.1602140 | O | 52.4778085 | 3.0322739 | 12.2275968 |  |
| O | 53.1512716 | 0.9051908 | 12.3931855 | O | 53.0966655 | 0.8784784 | 12.4481733 |  |
| N | 52.5742097 | 2.5268829 | 15.8732165 | N | 52.5748500 | 2.5141159 | 15.8633618 |  |
| C | 51.9070000 | 1.7130000 | 16.8920000 | C | 51.9070000 | 1.7130000 | 16.8920000 | f |
| N | 38.7746314 | 9.8021902 | 16.6100339 | N | 39.3816810 | 10.0379034 | 16.5431265 |  |
| C | 39.3775570 | 9.6152876 | 15.3188627 | C | 39.4682640 | 9.7181015 | 15.0712514 |  |
| C | 38.4531693 | 8.8696919 | 14.3415663 | C | 38.3597511 | 8.8973477 | 14.4550816 |  |
| O | 37.2482572 | 8.6653073 | 14.6654210 | O | 37.2220508 | 8.8943823 | 15.0021121 |  |
| C | 40.7790119 | 8.9392674 | 15.2763062 | C | 40.8607878 | 9.1027599 | 15.2674704 |  |
| C | 41.6805784 | 9.4139539 | 16.4135698 | C | 40.8780779 | 9.7528877 | 16.6599226 |  |
| S | 43.4684662 | 9.1540958 | 16.0070579 | S | 43.9385221 | 9.0634742 | 16.0054895 |  |
| C | 43.5069457 | 7.5499055 | 15.1672734 | C | 44.0368020 | 7.4498468 | 15.1714685 |  |
| C | 44.1863760 | 8.7134481 | 17.6469965 | C | 44.6164375 | 8.6975794 | 17.6676209 |  |
| C | 45.7062991 | 8.7719240 | 17.6803966 | C | 46.1159573 | 8.9287660 | 17.7669206 |  |
| O | 46.2386740 | 7.7160366 | 16.8780941 | O | 46.7709063 | 7.8868954 | 17.0349569 |  |
| C | 46.3747676 | 10.1058444 | 17.2062405 | C | 46.6045201 | 10.2903406 | 17.1882839 |  |
| O | 47.0734569 | 10.7610090 | 18.2189360 | O | 47.2461624 | 11.1068747 | 18.1245707 |  |
| C | 47.2351254 | 9.6205948 | 16.0011975 | C | 47.4714321 | 9.8459560 | 15.9712087 |  |
| O | 48.3138601 | 10.3953628 | 15.6398384 | O | 48.4855161 | 10.7043225 | 15.5755099 |  |
| C | 47.5175500 | 8.1564205 | 16.3924309 | C | 47.9094191 | 8.4255221 | 16.3754378 |  |
| O | 38.9400047 | 8.5221995 | 13.2305773 | O | 38.6692749 | 8.3264494 | 13.3864969 |  |
| N | 50.9402620 | 4.6322449 | 14.9940434 | N | 50.9991503 | 4.6312875 | 14.9412993 |  |
| C | 50.7931425 | 5.3309089 | 16.1282735 | C | 51.0043736 | 5.4237289 | 16.0218288 |  |
| N | 49.8646718 | 6.2368690 | 16.4249260 | N | 50.1827559 | 6.4296983 | 16.3081914 |  |
| C | 49.0134919 | 6.4125179 | 15.4053472 | C | 49.2647038 | 6.5972683 | 15.3451887 |  |
| C | 49.0348486 | 5.7522799 | 14.1702080 | C | 49.1332242 | 5.8440528 | 14.1694598 |  |
| C | 50.0810000 | 4.7970000 | 13.9630000 | C | 50.0810000 | 4.7970000 | 13.9630000 | f |
| N | 50.2129057 | 4.0913492 | 12.8402692 | N | 50.0829559 | 3.9959680 | 12.8919506 |  |
| N | 48.0059751 | 6.2030531 | 13.3621965 | N | 48.0692663 | 6.3061971 | 13.4162104 |  |
| C | 47.3856550 | 7.1087943 | 14.0783692 | C | 47.5727693 | 7.3011078 | 14.1129817 |  |
| N | 47.9436541 | 7.2873604 | 15.3352837 | N | 48.2588701 | 7.5411985 | 15.2917607 |  |
| H | 39.0641963 | 10.6737027 | 17.0410327 | H | 49.4379734 | 4.2381455 | 12.1481992 |  |
| H | 38.9290813 | 9.0242445 | 17.2463147 | H | 50.9605188 | 3.4670704 | 12.6436448 |  |
| H | 49.5776268 | 4.3168060 | 12.0831688 | H | 49.1906791 | 10.7382350 | 16.2873990 |  |
| H | 51.1030199 | 3.5689983 | 12.6142815 | H | 48.0691438 | 10.6331152 | 18.4812582 |  |
| H | 49.0607585 | 10.3541445 | 16.3204352 | H | 51.2714027 | 2.3544158 | 17.5254559 |  |
| H | 47.9244387 | 10.2688936 | 18.4725478 | H | 51.2724708 | 0.9304568 | 16.4398351 |  |
| H | 51.2973786 | 2.3494339 | 17.5552957 | H | 52.6714011 | 1.2273533 | 17.5161616 |  |
| H | 51.2475258 | 0.9563740 | 16.4314931 | H | 49.1776686 | 8.6510847 | 11.1039127 |  |
| H | 52.6722508 | 1.1946168 | 17.4880004 | H | 49.0886561 | 10.3772912 | 10.6672038 |  |
| H | 49.1697297 | 8.6719997 | 11.0416179 | H | 49.3690541 | 9.9019181 | 12.3592494 |  |
| H | 49.0687172 | 10.4195604 | 10.7035058 | H | 47.3713772 | 11.2512140 | 13.1225473 |  |
| H | 49.3901237 | 9.8514171 | 12.3593195 | H | 46.9211573 | 7.9072487 | 10.4466215 |  |
| H | 47.3924733 | 11.2140823 | 13.1790492 | H | 44.9334449 | 11.1945616 | 13.5949878 |  |
| H | 46.9079135 | 7.9182799 | 10.4490623 | H | 44.4847886 | 7.8372310 | 10.9457004 |  |
| H | 44.9562334 | 11.1767689 | 13.6498086 | H | 42.7731137 | 8.7349239 | 12.1998429 |  |
| H | 44.4785817 | 7.8431698 | 10.9706474 | H | 42.6792501 | 15.9175338 | 14.0401949 |  |
| H | 42.7664446 | 8.7596540 | 12.2083472 | H | 41.1072608 | 15.0821297 | 13.9267654 |  |
| H | 42.6800825 | 15.9047271 | 14.0057576 | H | 43.8576935 | 13.7410257 | 14.2342267 |  |
| H | 41.1063413 | 15.0638890 | 13.9284755 | H | 41.9092329 | 13.7398611 | 11.8716319 |  |
| H | 43.8733163 | 13.7602935 | 14.2509713 | H | 43.3810930 | 12.7757192 | 11.9959557 |  |
| H | 41.8442202 | 13.6025619 | 11.9631923 | H | 42.5854870 | 11.5681296 | 13.8749470 |  |
| H | 43.3164999 | 12.6416758 | 12.0912806 | H | 42.1837182 | 12.3612156 | 15.4168222 |  |
| H | 42.6387071 | 11.5510315 | 14.0830631 | H | 41.0611062 | 12.4747306 | 14.0469624 |  |
| H | 42.2371643 | 12.4447980 | 15.5766042 | H | 43.8647864 | 14.7537774 | 10.6088092 |  |
| H | 41.0967836 | 12.4257829 | 14.2242106 | H | 43.2854185 | 15.8658545 | 11.8637254 |  |
| H | 43.7467081 | 14.5381531 | 10.5722980 | H | 44.7640128 | 14.9096569 | 12.1353792 |  |
| H | 43.2273991 | 15.7208056 | 11.7892321 | H | 43.7650586 | 15.9170717 | 16.0775570 |  |
| H | 44.7114586 | 14.7697320 | 12.0485873 | H | 42.3568428 | 14.8139432 | 18.4611723 |  |
| H | 43.8155166 | 15.8795132 | 16.0784899 | H | 43.1680458 | 16.4023433 | 18.5139839 |  |
| H | 42.3498349 | 14.8129776 | 18.4429992 | H | 44.6757792 | 14.6961376 | 19.4649239 |  |
| H | 43.1579365 | 16.3993753 | 18.5204269 | H | 45.3610194 | 15.3116984 | 17.9598043 |  |
| H | 44.6409125 | 14.6798629 | 19.4936907 | H | 45.6568492 | 12.8822821 | 17.9412594 |  |
| H | 45.3666440 | 15.3320850 | 18.0239337 | H | 44.4300170 | 13.2849618 | 16.7268256 |  |
| H | 45.6719134 | 12.8863845 | 17.9944313 | H | 43.8751722 | 10.7987040 | 20.5258483 |  |
| H | 44.5286326 | 13.3343361 | 16.7136916 | H | 45.4213676 | 11.2934854 | 19.7396931 |  |
| H | 43.6222277 | 10.8426565 | 20.4601667 | H | 44.4859180 | 12.4656920 | 20.7444744 |  |
| H | 45.2485762 | 11.2258293 | 19.7770197 | H | 52.5243670 | 10.2899427 | 18.8377930 |  |
| H | 44.3131598 | 12.4731010 | 20.6878388 | H | 51.5695730 | 9.5699337 | 20.1986709 |  |
| H | 52.4143679 | 10.3996363 | 18.8125405 | H | 51.9218366 | 8.6181109 | 18.7278470 |  |
| H | 51.5504671 | 9.6143501 | 20.1978058 | H | 39.7565189 | 6.8083323 | 21.1705830 |  |
| H | 52.0882276 | 8.6496551 | 18.7906890 | H | 38.1861741 | 7.5876487 | 21.0430755 |  |
| H | 39.6551064 | 6.8126260 | 21.2595673 | H | 40.8781351 | 8.7659671 | 20.0921249 |  |
| H | 38.1408672 | 7.6309563 | 20.9217478 | H | 40.0947775 | 9.1544163 | 21.6166244 |  |
| H | 40.9286825 | 8.7121355 | 20.1646090 | H | 37.1867477 | 9.1745152 | 19.8523202 |  |
| H | 40.1145721 | 9.0968147 | 21.6770476 | H | 40.9948605 | 11.2173602 | 19.7504162 |  |
| H | 37.2255792 | 9.3531725 | 20.0462759 | H | 36.1124861 | 11.1376676 | 18.8283842 |  |
| H | 41.1673886 | 11.0754915 | 19.6862408 | H | 39.9200090 | 13.1720672 | 18.6973659 |  |
| H | 36.2527697 | 11.3878162 | 19.0642452 | H | 37.4533265 | 13.1662167 | 18.2606260 |  |
| H | 40.1938510 | 13.1008266 | 18.6588897 | H | 38.2550442 | 5.1865307 | 19.9172848 |  |
| H | 37.7139666 | 13.2880143 | 18.3708081 | H | 37.9587807 | 3.9626535 | 17.9904224 |  |
| H | 38.0131721 | 5.3225306 | 19.8748851 | H | 37.4189202 | 6.7563836 | 16.8356864 |  |
| H | 37.9239999 | 3.9725374 | 17.9846581 | H | 36.2342180 | 5.6986833 | 17.6320094 |  |
| H | 37.5165223 | 6.7718116 | 16.7729213 | H | 36.5097355 | 5.4639713 | 13.7080678 |  |
| H | 36.2705908 | 5.7925738 | 17.5782861 | H | 36.8835697 | 6.8286274 | 14.7035056 |  |
| H | 36.5747724 | 5.3743869 | 13.6787389 | H | 40.6038540 | 4.3670105 | 18.5587192 |  |
| H | 36.9363603 | 6.7911512 | 14.6115786 | H | 41.9194097 | 4.6216412 | 15.9015260 |  |
| H | 40.5800665 | 4.2113926 | 18.5538159 | H | 42.0118777 | 3.1020289 | 16.8053116 |  |
| H | 41.9304942 | 4.7505799 | 15.9649416 | H | 42.9788444 | 4.5843863 | 18.7768217 |  |
| H | 42.0517710 | 3.1300569 | 16.6570504 | H | 43.2844680 | 5.7901198 | 17.5286334 |  |
| H | 42.9171837 | 4.3036025 | 18.8385864 | H | 44.3514406 | 2.9261654 | 17.7202761 |  |
| H | 43.1903917 | 5.7237896 | 17.8377967 | H | 45.7040071 | 3.3156384 | 15.6762568 |  |
| H | 44.3610736 | 2.9167021 | 17.4346064 | H | 43.9723112 | 3.3852352 | 15.2884545 |  |
| H | 45.8115102 | 3.7975281 | 15.6732106 | H | 44.9247339 | 4.8848049 | 15.4182048 |  |
| H | 44.1432121 | 4.1938744 | 15.2145993 | H | 46.5744075 | 3.9830358 | 17.9759876 |  |
| H | 45.2186458 | 5.4422095 | 15.9180969 | H | 45.8915393 | 5.6020332 | 17.7362268 |  |
| H | 46.5321056 | 3.9120321 | 18.1538011 | H | 45.4620028 | 4.6593616 | 19.1910673 |  |
| H | 45.7674110 | 5.4980855 | 18.3530459 | H | 32.8097891 | 5.6195248 | 15.4920202 |  |
| H | 45.3050926 | 4.1406440 | 19.4271111 | H | 33.1941324 | 5.1824724 | 17.1597072 |  |
| H | 32.7841378 | 5.5825814 | 15.4965335 | H | 31.5316114 | 5.6585827 | 16.7399838 |  |
| H | 33.1511724 | 5.1573599 | 17.1715470 | H | 31.0959982 | 7.7552695 | 17.8857794 |  |
| H | 31.5208765 | 5.7341334 | 16.7515499 | H | 34.8597747 | 7.2253596 | 15.8533073 |  |
| H | 31.2555549 | 7.8759913 | 17.8887406 | H | 31.7009665 | 10.1089908 | 18.3911813 |  |
| H | 34.9185441 | 7.0676290 | 15.7683989 | H | 35.4904965 | 9.5492274 | 16.3826599 |  |
| H | 32.0379222 | 10.1876812 | 18.3475090 | H | 33.9007213 | 11.0312090 | 17.6398378 |  |
| H | 35.7499718 | 9.3314314 | 16.2865799 | H | 30.6819062 | 9.7335765 | 14.4866526 |  |
| H | 34.2878692 | 10.9378475 | 17.5476400 | H | 32.4078549 | 9.6478860 | 14.9119987 |  |
| H | 30.6814146 | 9.7192073 | 14.4951184 | H | 31.9159548 | 10.1602460 | 13.2722369 |  |
| H | 32.4130888 | 9.6612146 | 14.9053731 | H | 30.9617783 | 7.8654531 | 12.8493213 |  |
| H | 31.8977932 | 10.1605816 | 13.2681323 | H | 31.4085620 | 7.3786995 | 14.5024955 |  |
| H | 30.9829779 | 7.8476650 | 12.8574420 | H | 33.8170878 | 8.2914375 | 13.2784494 |  |
| H | 31.4390233 | 7.3755859 | 14.5123076 | H | 32.5232858 | 4.6415595 | 12.0455906 |  |
| H | 33.8333133 | 8.3283973 | 13.2777269 | H | 31.4610826 | 5.6223826 | 12.9919858 |  |
| H | 32.6450404 | 4.6440184 | 12.0624712 | H | 34.6890942 | 5.3193686 | 11.6560603 |  |
| H | 31.5371505 | 5.6071580 | 12.9728514 | H | 35.2868722 | 6.8922940 | 12.0155174 |  |
| H | 34.7949992 | 5.3868155 | 11.6867187 | H | 33.5997324 | 14.5828628 | 13.5460682 |  |
| H | 35.3480877 | 6.9683703 | 12.0938867 | H | 33.1185005 | 14.4638531 | 15.2488632 |  |
| H | 33.5313799 | 14.4736193 | 13.6057993 | H | 33.8562823 | 15.9672861 | 14.6380536 |  |
| H | 33.1487465 | 14.5715137 | 15.3347177 | H | 36.2100232 | 16.2669855 | 15.1113641 |  |
| H | 33.8854448 | 15.9682892 | 14.5105456 | H | 34.6851560 | 12.2453437 | 14.7938440 |  |
| H | 36.1957818 | 16.2801429 | 15.1025633 | H | 38.4691452 | 15.3652973 | 15.6313546 |  |
| H | 34.7100934 | 12.2436211 | 14.7787083 | H | 36.9278201 | 11.3537308 | 15.3057174 |  |
| H | 38.4623736 | 15.3970179 | 15.6204050 | H | 39.7661979 | 13.4487608 | 15.9478356 |  |
| H | 36.9695319 | 11.3696050 | 15.3268191 | H | 34.7804576 | 13.6765594 | 8.8662616 |  |
| H | 39.7512045 | 13.5075120 | 15.9484229 | H | 35.2740243 | 14.6542139 | 10.2669948 |  |
| H | 34.7132653 | 13.7459036 | 8.9286437 | H | 36.5101980 | 13.9179922 | 9.2288415 |  |
| H | 35.3807291 | 14.6526305 | 10.3037323 | H | 37.5389640 | 12.7481239 | 11.2686089 |  |
| H | 36.4779411 | 13.8712298 | 9.1500084 | H | 33.4844031 | 11.9382698 | 10.0524914 |  |
| H | 37.5862703 | 12.6151410 | 11.0950626 | H | 37.4924587 | 10.7804829 | 12.7527257 |  |
| H | 33.4213297 | 12.0448308 | 10.1465523 | H | 33.4344020 | 9.9725252 | 11.5582281 |  |
| H | 37.5405457 | 10.6089862 | 12.5393375 | H | 36.0682060 | 9.1206298 | 13.8133685 |  |
| H | 33.3720720 | 10.0452034 | 11.6126943 | H | 38.9181302 | 6.6944635 | 10.6729505 |  |
| H | 36.1409657 | 8.9861095 | 13.6334943 | H | 37.3980731 | 6.4747667 | 9.7575099 |  |
| H | 38.9277391 | 6.6995331 | 10.5903743 | H | 37.9405170 | 8.1187812 | 10.2018155 |  |
| H | 37.3271178 | 6.4282191 | 9.8441960 | H | 37.6120431 | 7.4665830 | 12.4314456 |  |
| H | 37.9042233 | 8.0942237 | 10.1294266 | H | 43.5281514 | 5.0105524 | 12.4789249 |  |
| H | 37.7989917 | 7.5953303 | 12.4092927 | H | 42.0169652 | 4.0814365 | 12.7702097 |  |
| H | 42.5068533 | 4.6256457 | 11.1115658 | H | 42.4703699 | 4.6139334 | 11.1142240 |  |
| H | 43.5188572 | 5.0000040 | 12.5138344 | H | 40.1776074 | 7.1575741 | 13.2137638 |  |
| H | 41.9922312 | 4.0763038 | 12.7426907 | H | 40.4162595 | 5.5263177 | 13.7601906 |  |
| H | 40.1556036 | 7.1680635 | 13.1457645 | H | 55.3010994 | 3.9222844 | 14.7720527 |  |
| H | 40.3542116 | 5.5145192 | 13.6708111 | H | 53.6165931 | 4.2127762 | 14.2070274 |  |
| H | 55.2856120 | 3.9361841 | 14.7946334 | H | 55.2715164 | 1.7521387 | 13.3918989 |  |
| H | 53.6111252 | 4.2043766 | 14.1966993 | H | 55.1145354 | 3.2416523 | 12.4122052 |  |
| H | 55.3056764 | 1.7614781 | 13.4109143 | H | 51.9859590 | 3.1725509 | 15.3265440 |  |
| H | 55.1841607 | 3.2591491 | 12.4375655 | H | 39.5114324 | 10.6786843 | 14.5385844 |  |
| H | 51.9882190 | 3.2018202 | 15.3564570 | H | 41.6267299 | 9.4194941 | 14.5540532 |  |
| H | 39.4871514 | 10.6186044 | 14.8739612 | H | 40.8316975 | 8.0070934 | 15.3200785 |  |
| H | 41.2282881 | 9.1531067 | 14.2959141 | H | 45.0346146 | 7.0181084 | 15.3098756 |  |
| H | 40.6640524 | 7.8486015 | 15.3594008 | H | 43.2687506 | 6.7550389 | 15.5408616 |  |
| H | 41.4635656 | 8.8797141 | 17.3501728 | H | 43.8571199 | 7.6450923 | 14.1068871 |  |
| H | 41.6191271 | 10.4955297 | 16.5939666 | H | 44.3654620 | 7.6640835 | 17.9491824 |  |
| H | 44.5592591 | 7.2484761 | 15.1813668 | H | 44.0918157 | 9.3790008 | 18.3501315 |  |
| H | 42.8850147 | 6.8242916 | 15.7032231 | H | 46.4256075 | 8.8800462 | 18.8284172 |  |
| H | 43.1589823 | 7.7194147 | 14.1450463 | H | 45.7444534 | 10.8636737 | 16.8117932 |  |
| H | 43.8086036 | 7.7148517 | 17.9098650 | H | 46.7713193 | 9.7324676 | 15.1296252 |  |
| H | 43.7685599 | 9.4633047 | 18.3280763 | H | 48.7767733 | 8.4544295 | 17.0535136 |  |
| H | 45.9966199 | 8.6219421 | 18.7374446 | H | 51.7885349 | 5.1990449 | 16.7563598 |  |
| H | 45.6051696 | 10.8090165 | 16.8494774 | H | 46.7206900 | 7.9087376 | 13.8162046 |  |
| H | 46.5500180 | 9.5716813 | 15.1366556 | H | 39.0887559 | 11.0092060 | 16.7416321 |  |
| H | 48.2793757 | 8.0967915 | 17.1899071 | H | 38.8298971 | 9.3596164 | 17.0901415 |  |
| H | 51.5380506 | 5.1139362 | 16.9046547 | H | 41.0885311 | 9.1108182 | 17.5180374 |  |
| H | 46.5328160 | 7.6968988 | 13.7421339 | H | 41.4319059 | 10.6942922 | 16.7307111 |  |

| ***AzeJ_pWT*** Reactant | ***AzeJ_pWT*** Product | Fixed (f) |
| --- | --- | --- |

| C | 48.8200000 | 9.6360000 | 11.4410000 | C | 48.8200000 | 9.6360000 | 11.4410000 | f |
| --- | --- | --- | --- | --- | --- | --- | --- | --- |
| C | 47.3413146 | 9.6019507 | 11.7623543 | C | 47.3332798 | 9.6102826 | 11.7228268 |  |
| C | 46.7737454 | 10.4961231 | 12.6863746 | C | 46.7494628 | 10.5027648 | 12.6396813 |  |
| C | 46.4837389 | 8.6609285 | 11.1680145 | C | 46.4871573 | 8.6732844 | 11.1082083 |  |
| C | 45.4112079 | 10.4551911 | 13.0041031 | C | 45.3820868 | 10.4652059 | 12.9286984 |  |
| C | 45.1228648 | 8.5958892 | 11.4835093 | C | 45.1185220 | 8.6156006 | 11.3928329 |  |
| C | 44.5742502 | 9.4996916 | 12.4087721 | C | 44.5540661 | 9.5187499 | 12.3072843 |  |
| O | 43.2530124 | 9.4730365 | 12.7628993 | O | 43.2254729 | 9.4964988 | 12.6285322 |  |
| C | 42.1220000 | 15.0210000 | 14.3510000 | C | 42.1220000 | 15.0210000 | 14.3510000 | f |
| C | 41.9712136 | 14.9921685 | 15.8828873 | C | 41.9806664 | 15.0091648 | 15.8814122 |  |
| O | 40.9924991 | 14.4531087 | 16.4295065 | O | 40.9838712 | 14.5063998 | 16.4393325 |  |
| C | 42.8283957 | 13.7331415 | 13.8441851 | C | 42.8519060 | 13.7501776 | 13.8399695 |  |
| C | 42.9722565 | 13.7331533 | 12.3108895 | C | 42.9337940 | 13.7272570 | 12.3027091 |  |
| C | 42.1116890 | 12.4585888 | 14.3145006 | C | 42.1977715 | 12.4644597 | 14.3627939 |  |
| C | 43.8372502 | 14.8576699 | 11.7389233 | C | 43.7056712 | 14.8877890 | 11.6725364 |  |
| N | 42.9923144 | 15.5122752 | 16.6047790 | N | 43.0081800 | 15.5047953 | 16.6007870 |  |
| C | 43.1990000 | 15.3970000 | 18.0630000 | C | 43.1990000 | 15.3970000 | 18.0630000 | f |
| C | 44.5276053 | 14.6989969 | 18.3706931 | C | 44.5299796 | 14.7100288 | 18.3854955 |  |
| C | 44.6447890 | 13.2892966 | 17.7891480 | C | 44.6623128 | 13.2969748 | 17.8135055 |  |
| S | 43.4278848 | 12.0965115 | 18.4750205 | S | 43.4716267 | 12.0844355 | 18.5079600 |  |
| C | 44.2891905 | 11.6339265 | 20.0219419 | C | 44.3811984 | 11.5746871 | 20.0101294 |  |
| C | 51.6880000 | 9.6280000 | 19.1070000 | C | 51.6880000 | 9.6280000 | 19.1070000 | f |
| C | 50.3574528 | 9.9151170 | 18.3942023 | C | 50.3687459 | 10.0508829 | 18.4553327 |  |
| O | 49.2831183 | 9.6409667 | 19.0115595 | O | 49.3019422 | 9.7717295 | 19.0805796 |  |
| O | 50.4195347 | 10.3801658 | 17.2286394 | O | 50.4298995 | 10.6053388 | 17.3296996 |  |
| C | 39.1800000 | 7.5290000 | 20.5670000 | C | 39.1800000 | 7.5290000 | 20.5670000 | f |
| C | 39.0677613 | 6.9200886 | 19.1620003 | C | 38.9362610 | 6.9971373 | 19.1483070 |  |
| O | 39.4773754 | 7.5208101 | 18.1554234 | O | 39.2033705 | 7.6989997 | 18.1473698 |  |
| C | 39.9025924 | 8.8744041 | 20.6040521 | C | 39.9349576 | 8.8607295 | 20.5992648 |  |
| C | 39.2168986 | 10.0492398 | 19.9298369 | C | 39.2772994 | 10.0685105 | 19.9498901 |  |
| C | 37.8302620 | 10.1056301 | 19.7159349 | C | 37.9075720 | 10.1346345 | 19.6424824 |  |
| C | 39.9929190 | 11.1493484 | 19.5189185 | C | 40.0747798 | 11.1877804 | 19.6460523 |  |
| C | 37.2382329 | 11.2285593 | 19.1204813 | C | 37.3559938 | 11.2828049 | 19.0547717 |  |
| C | 39.4111808 | 12.2694754 | 18.9156861 | C | 39.5325817 | 12.3326782 | 19.0540038 |  |
| C | 38.0225777 | 12.3152586 | 18.7214576 | C | 38.1635843 | 12.3868875 | 18.7603303 |  |
| N | 38.5205595 | 5.6826609 | 19.0621705 | N | 38.4448565 | 5.7504025 | 19.0342604 |  |
| C | 38.2620000 | 4.9980000 | 17.7770000 | C | 38.2620000 | 4.9980000 | 17.7770000 | f |
| C | 39.5775576 | 4.9575886 | 16.9258677 | C | 39.5751260 | 5.0608403 | 16.9182522 |  |
| O | 39.6033840 | 5.3844403 | 15.7737525 | O | 39.6226435 | 5.6458426 | 15.8366037 |  |
| C | 37.1553868 | 5.7110255 | 16.9878014 | C | 36.9841887 | 5.3689214 | 17.0134254 |  |
| C | 36.8850974 | 5.0401488 | 15.6429553 | C | 36.9387615 | 4.6550073 | 15.6621606 |  |
| N | 36.7025479 | 5.8918419 | 14.6032170 | N | 36.7543649 | 5.4570057 | 14.5791613 |  |
| O | 36.7935217 | 3.8222304 | 15.5509595 | O | 37.0331606 | 3.4376085 | 15.5962793 |  |
| N | 40.6477837 | 4.4166988 | 17.5443341 | N | 40.6098744 | 4.4044789 | 17.4804734 |  |
| C | 41.9510000 | 4.2010000 | 16.9160000 | C | 41.9510000 | 4.2010000 | 16.9160000 | f |
| C | 43.1075191 | 4.5709857 | 17.8533858 | C | 43.0372005 | 4.4497810 | 17.9729149 |  |
| C | 44.4664277 | 3.9439007 | 17.4832996 | C | 44.3931426 | 3.7724797 | 17.6932425 |  |
| C | 44.9964341 | 4.3726187 | 16.1075328 | C | 45.1029276 | 4.3079909 | 16.4433680 |  |
| C | 45.5122688 | 4.2502387 | 18.5638606 | C | 45.3121176 | 3.9027135 | 18.9155981 |  |
| C | 32.5930000 | 5.8730000 | 16.5430000 | C | 32.5930000 | 5.8730000 | 16.5430000 | f |
| C | 32.9876540 | 7.3065188 | 16.8197886 | C | 33.1397236 | 7.2730426 | 16.7140334 |  |
| C | 32.1806166 | 8.1507641 | 17.6005086 | C | 32.5222948 | 8.1952624 | 17.5765174 |  |
| C | 34.1868086 | 7.8287564 | 16.3039207 | C | 34.2872842 | 7.6879054 | 16.0168811 |  |
| C | 32.5599639 | 9.4733681 | 17.8621847 | C | 33.0316857 | 9.4893145 | 17.7367953 |  |
| C | 34.5699289 | 9.1490051 | 16.5612214 | C | 34.7931450 | 8.9831490 | 16.1664820 |  |
| C | 33.7565561 | 9.9787109 | 17.3417519 | C | 34.1680494 | 9.8919206 | 17.0258304 |  |
| C | 31.6810000 | 9.4790000 | 14.1040000 | C | 31.6810000 | 9.4790000 | 14.1040000 | f |
| C | 31.7237736 | 8.0311444 | 13.6307344 | C | 31.6588489 | 8.0333315 | 13.6226309 |  |
| N | 33.0579120 | 7.6731339 | 13.1497310 | N | 32.9645886 | 7.6313056 | 13.0964163 |  |
| C | 33.3531870 | 6.5362604 | 12.5186312 | C | 33.2325115 | 6.4336232 | 12.5722839 |  |
| N | 32.4168569 | 5.5812224 | 12.3782061 | N | 32.2776505 | 5.4903659 | 12.5241933 |  |
| N | 34.5744282 | 6.3326131 | 12.0136388 | N | 34.4516808 | 6.1562403 | 12.0976288 |  |
| C | 33.8900000 | 14.8690000 | 14.5720000 | C | 33.8900000 | 14.8690000 | 14.5720000 | f |
| C | 35.2686563 | 14.3131430 | 14.8736349 | C | 35.2820696 | 14.3515690 | 14.8800270 |  |
| C | 36.3262366 | 15.1415779 | 15.2869121 | C | 36.3135316 | 15.2061270 | 15.3037079 |  |
| C | 35.5406374 | 12.9381022 | 14.7622282 | C | 35.5949376 | 12.9864178 | 14.7447151 |  |
| C | 37.5990809 | 14.6328805 | 15.5760066 | C | 37.6031657 | 14.7337477 | 15.5799682 |  |
| C | 36.8027104 | 12.4137206 | 15.0492414 | C | 36.8753913 | 12.4994348 | 15.0166474 |  |
| C | 37.8493336 | 13.2568485 | 15.4539011 | C | 37.8943808 | 13.3710206 | 15.4312048 |  |
| O | 39.0675117 | 12.7056183 | 15.7162708 | O | 39.1395802 | 12.8633134 | 15.6723684 |  |
| C | 35.5190000 | 13.7510000 | 9.6800000 | C | 35.5190000 | 13.7510000 | 9.6800000 | f |
| C | 35.5228517 | 12.5043341 | 10.5300930 | C | 35.5220380 | 12.4717819 | 10.4759697 |  |
| C | 36.6997255 | 12.0089734 | 11.1133728 | C | 36.6903550 | 12.0081982 | 11.1002103 |  |
| C | 34.3295312 | 11.8120927 | 10.7914508 | C | 34.3490113 | 11.7178937 | 10.6415409 |  |
| C | 36.6917158 | 10.8924956 | 11.9556781 | C | 36.6925874 | 10.8500029 | 11.8800566 |  |
| C | 34.3031279 | 10.7013652 | 11.6340137 | C | 34.3325617 | 10.5575950 | 11.4172673 |  |
| C | 35.4805543 | 10.2528368 | 12.2468329 | C | 35.5041351 | 10.1313171 | 12.0511409 |  |
| O | 35.3886692 | 9.1956542 | 13.1103060 | O | 35.4416130 | 9.0051369 | 12.8363194 |  |
| C | 37.8850000 | 7.0690000 | 10.5480000 | C | 37.8850000 | 7.0690000 | 10.5480000 | f |
| O | 37.2811956 | 7.0523408 | 11.8328411 | O | 37.2878609 | 6.7133310 | 11.7957184 |  |
| C | 42.4750000 | 4.8880000 | 12.1800000 | C | 42.4750000 | 4.8880000 | 12.1800000 | f |
| C | 41.7781237 | 6.2173059 | 12.3484375 | C | 41.7703007 | 6.2202557 | 12.3223178 |  |
| N | 40.6850398 | 6.2616139 | 13.1238416 | N | 40.7452515 | 6.3029703 | 13.1994731 |  |
| O | 42.2148166 | 7.2282826 | 11.7734524 | O | 42.1226105 | 7.1997674 | 11.6547052 |  |
| C | 54.3690000 | 3.4430000 | 14.4320000 | C | 54.3690000 | 3.4430000 | 14.4320000 | f |
| C | 53.9036247 | 2.5092153 | 15.5841013 | C | 53.9089096 | 2.5253983 | 15.5997532 |  |
| O | 54.7123966 | 1.7726632 | 16.1533246 | O | 54.7272821 | 1.8270106 | 16.2035314 |  |
| C | 54.5956060 | 2.6231589 | 13.1486296 | C | 54.5782469 | 2.6066536 | 13.1560444 |  |
| C | 53.2386015 | 2.1684193 | 12.5641420 | C | 53.2181389 | 2.1331774 | 12.5888332 |  |
| O | 52.5155408 | 3.1284715 | 12.1402162 | O | 52.4693073 | 3.0811403 | 12.1880832 |  |
| O | 52.9461711 | 0.9625577 | 12.5816419 | O | 52.9555900 | 0.9193823 | 12.5905382 |  |
| N | 52.5791139 | 2.5393946 | 15.8869804 | N | 52.5779466 | 2.5221373 | 15.8724831 |  |
| C | 51.9070000 | 1.7130000 | 16.8920000 | C | 51.9070000 | 1.7130000 | 16.8920000 | f |
| N | 38.9572120 | 9.7899815 | 16.7003589 | N | 39.6068773 | 10.3514443 | 16.5841312 |  |
| C | 39.5951730 | 9.6396236 | 15.3592241 | C | 39.4858865 | 9.7309841 | 15.2204048 |  |
| C | 38.5735569 | 8.9693806 | 14.4216878 | C | 38.3815166 | 8.7358727 | 15.0148653 |  |
| O | 37.3844823 | 8.8927083 | 14.8500620 | O | 37.9163263 | 8.0718231 | 16.0261659 |  |
| C | 40.9262397 | 8.8850013 | 15.3729354 | C | 40.9194419 | 9.1359228 | 15.3405229 |  |
| C | 41.8685715 | 9.3604381 | 16.4794237 | C | 41.0582953 | 9.8761178 | 16.6804366 |  |
| S | 43.6402102 | 9.0897209 | 16.0577130 | S | 43.9494135 | 8.9291728 | 15.9455271 |  |
| C | 43.6547466 | 7.4750040 | 15.2385392 | C | 44.0119109 | 7.3143194 | 15.1164474 |  |
| C | 44.3322696 | 8.6911022 | 17.7220675 | C | 44.5543794 | 8.5601947 | 17.6373117 |  |
| C | 45.8455616 | 8.7928204 | 17.7442746 | C | 46.0478435 | 8.8033033 | 17.7974359 |  |
| O | 46.3812558 | 7.7517113 | 16.9321888 | O | 46.7388585 | 7.7878683 | 17.0663770 |  |
| C | 46.4496361 | 10.1471709 | 17.2418951 | C | 46.5362772 | 10.1868877 | 17.2688958 |  |
| O | 47.1278004 | 10.8495025 | 18.2312738 | O | 47.1779746 | 10.9695746 | 18.2325945 |  |
| C | 47.3070115 | 9.6803667 | 16.0249376 | C | 47.4067361 | 9.7837132 | 16.0424787 |  |
| O | 48.3565503 | 10.4857563 | 15.6504430 | O | 48.3991909 | 10.6691738 | 15.6570705 |  |
| C | 47.6356472 | 8.2234089 | 16.4064079 | C | 47.8709429 | 8.3651078 | 16.4215024 |  |
| O | 38.9845338 | 8.5873617 | 13.3116311 | O | 37.9426463 | 8.5549978 | 13.8798606 |  |
| N | 50.9722674 | 4.6466705 | 14.9694716 | N | 50.9933533 | 4.6214332 | 14.9446692 |  |
| C | 50.8780428 | 5.3778293 | 16.0872855 | C | 50.9866339 | 5.3952018 | 16.0381206 |  |
| N | 49.9759288 | 6.3094995 | 16.3903409 | N | 50.1568392 | 6.3914730 | 16.3362757 |  |
| C | 49.0938059 | 6.4692743 | 15.3955759 | C | 49.2474714 | 6.5710528 | 15.3678570 |  |
| C | 49.0589592 | 5.7752883 | 14.1802978 | C | 49.1288967 | 5.8389249 | 14.1786154 |  |
| C | 50.0810000 | 4.7970000 | 13.9630000 | C | 50.0810000 | 4.7970000 | 13.9630000 | f |
| N | 50.1621378 | 4.0587866 | 12.8574139 | N | 50.0895045 | 4.0140684 | 12.8802234 |  |
| N | 48.0083920 | 6.2204934 | 13.3971088 | N | 48.0725689 | 6.3134477 | 13.4217035 |  |
| C | 47.4282373 | 7.1546486 | 14.1092793 | C | 47.5679088 | 7.2953494 | 14.1301607 |  |
| N | 48.0368908 | 7.3601793 | 15.3388500 | N | 48.2400968 | 7.5136227 | 15.3212321 |  |
| H | 39.1531580 | 10.7107801 | 17.1204544 | H | 38.5086587 | 7.9020884 | 16.8608872 |  |
| H | 39.1952382 | 9.0158694 | 17.3674121 | H | 49.4443768 | 4.2682865 | 12.1402578 |  |
| H | 37.9381466 | 9.7249440 | 16.5310947 | H | 50.9739994 | 3.4929983 | 12.6181709 |  |
| H | 49.5059176 | 4.2837130 | 12.1176477 | H | 49.1205056 | 10.6982196 | 16.3558183 |  |
| H | 51.0608643 | 3.5517896 | 12.5978512 | H | 48.0245101 | 10.5045590 | 18.5514966 |  |
| H | 49.1071734 | 10.4605051 | 16.3303152 | H | 51.2403988 | 2.3446122 | 17.5026172 |  |
| H | 47.9840914 | 10.3647778 | 18.5045878 | H | 51.3046910 | 0.9087733 | 16.4334116 |  |
| H | 51.2684882 | 2.3385824 | 17.5376222 | H | 52.6679136 | 1.2532578 | 17.5397126 |  |
| H | 51.2768236 | 0.9392645 | 16.4185878 | H | 49.1830147 | 8.6422191 | 11.1370828 |  |
| H | 52.6689877 | 1.2132380 | 17.5079903 | H | 49.0685474 | 10.3482533 | 10.6342122 |  |
| H | 49.1767197 | 8.6497240 | 11.1073250 | H | 49.3784699 | 9.9427361 | 12.3400246 |  |
| H | 49.0426547 | 10.3641890 | 10.6412012 | H | 47.3859971 | 11.2001901 | 13.1918916 |  |
| H | 49.4027198 | 9.9280742 | 12.3293073 | H | 46.9108604 | 7.9421004 | 10.4134523 |  |
| H | 47.4167935 | 11.2044376 | 13.2155581 | H | 44.9542001 | 11.1371092 | 13.6741535 |  |
| H | 46.8941050 | 7.9314558 | 10.4638875 | H | 44.4804034 | 7.8614699 | 10.9250807 |  |
| H | 44.9916888 | 11.1510154 | 13.7341935 | H | 42.7799951 | 8.7425370 | 12.1811467 |  |
| H | 44.4782880 | 7.8394804 | 11.0306699 | H | 42.6582495 | 15.9269847 | 14.0313896 |  |
| H | 42.7856354 | 8.7413617 | 12.2882782 | H | 41.1040900 | 15.0587841 | 13.9319309 |  |
| H | 42.6829778 | 15.9147964 | 14.0405642 | H | 43.8889886 | 13.7862655 | 14.2260433 |  |
| H | 41.1087767 | 15.0808741 | 13.9237863 | H | 41.9064447 | 13.6893862 | 11.8935795 |  |
| H | 43.8506419 | 13.7281963 | 14.2706080 | H | 43.4122655 | 12.7763881 | 12.0110413 |  |
| H | 41.9625922 | 13.7623642 | 11.8601611 | H | 42.6829976 | 11.5753990 | 13.9358783 |  |
| H | 43.4073298 | 12.7619857 | 12.0178144 | H | 42.2613759 | 12.3983335 | 15.4594066 |  |
| H | 42.5913798 | 11.5622379 | 13.8937627 | H | 41.1298798 | 12.4355203 | 14.0891597 |  |
| H | 42.1091914 | 12.3762825 | 15.4126081 | H | 43.8056935 | 14.7455251 | 10.5847505 |  |
| H | 41.0592427 | 12.4641356 | 13.9870179 | H | 43.2081919 | 15.8592889 | 11.8291903 |  |
| H | 43.9697883 | 14.7355841 | 10.6521683 | H | 44.7235007 | 14.9596230 | 12.0929461 |  |
| H | 43.3949733 | 15.8540485 | 11.9030072 | H | 43.7756888 | 15.9183433 | 16.0787322 |  |
| H | 44.8407498 | 14.8555651 | 12.1981537 | H | 42.3538453 | 14.8167409 | 18.4574461 |  |
| H | 43.7417319 | 15.9555783 | 16.0813752 | H | 43.1649969 | 16.4036611 | 18.5097827 |  |
| H | 42.3513857 | 14.8244570 | 18.4641112 | H | 44.6560987 | 14.6921229 | 19.4810407 |  |
| H | 43.1804784 | 16.4015782 | 18.5153031 | H | 45.3631713 | 15.3209723 | 17.9916084 |  |
| H | 44.6666756 | 14.6750257 | 19.4644643 | H | 45.6737502 | 12.8896075 | 17.9693015 |  |
| H | 45.3631839 | 15.3022879 | 17.9707684 | H | 44.4776304 | 13.2998276 | 16.7277057 |  |
| H | 45.6493638 | 12.8673664 | 17.9507172 | H | 43.8229140 | 10.7422056 | 20.4645665 |  |
| H | 44.4628212 | 13.3012086 | 16.7031446 | H | 45.3914261 | 11.2405742 | 19.7256242 |  |
| H | 43.6966123 | 10.8450557 | 20.5095020 | H | 44.4414283 | 12.3987806 | 20.7371119 |  |
| H | 45.2983759 | 11.2614022 | 19.7846701 | H | 52.4975206 | 10.3197557 | 18.8316292 |  |
| H | 44.3537812 | 12.4939539 | 20.7045118 | H | 51.5858536 | 9.5583374 | 20.1996014 |  |
| H | 52.4329886 | 10.3921426 | 18.8412483 | H | 51.9505714 | 8.6287931 | 18.7186960 |  |
| H | 51.5514824 | 9.5750673 | 20.1964455 | H | 39.7442653 | 6.7677635 | 21.1293752 |  |
| H | 52.0641750 | 8.6537546 | 18.7513222 | H | 38.1993932 | 7.6069444 | 21.0675075 |  |
| H | 39.7179744 | 6.8051200 | 21.2022040 | H | 40.9292616 | 8.7168574 | 20.1445749 |  |
| H | 38.1668213 | 7.5965635 | 20.9972463 | H | 40.1308525 | 9.1009706 | 21.6584106 |  |
| H | 40.9089370 | 8.7535829 | 20.1707863 | H | 37.2505406 | 9.2878817 | 19.8534284 |  |
| H | 40.0660826 | 9.1332310 | 21.6647248 | H | 41.1462659 | 11.1624353 | 19.8585150 |  |
| H | 37.1901788 | 9.2702514 | 20.0083300 | H | 36.2884165 | 11.3066443 | 18.8230389 |  |
| H | 41.0757587 | 11.1317522 | 19.6641060 | H | 40.1883763 | 13.1654660 | 18.7862106 |  |
| H | 36.1570308 | 11.2447625 | 18.9605165 | H | 37.7337500 | 13.2734686 | 18.2893106 |  |
| H | 40.0443778 | 13.0908853 | 18.5690740 | H | 38.2355148 | 5.2566472 | 19.8966700 |  |
| H | 37.5621416 | 13.1804432 | 18.2406011 | H | 38.1605320 | 3.9454737 | 18.0771151 |  |
| H | 38.1735042 | 5.2442225 | 19.9094968 | H | 36.8661087 | 6.4480946 | 16.9115662 |  |
| H | 37.9646992 | 3.9639398 | 17.9976446 | H | 36.1277821 | 5.0027435 | 17.6016354 |  |
| H | 37.4289884 | 6.7648169 | 16.8515548 | H | 36.8732293 | 5.0537254 | 13.6534401 |  |
| H | 36.2243187 | 5.6760497 | 17.5761677 | H | 36.9825775 | 6.4404600 | 14.6649325 |  |
| H | 36.6656131 | 5.5229728 | 13.6579747 | H | 40.4271792 | 3.9129939 | 18.3505063 |  |
| H | 36.9562464 | 6.8727627 | 14.6913423 | H | 42.0464911 | 4.8688716 | 16.0487819 |  |
| H | 40.5231080 | 4.0568502 | 18.4856100 | H | 42.0197353 | 3.1653223 | 16.5395514 |  |
| H | 41.9584030 | 4.7849583 | 15.9854189 | H | 42.6636170 | 4.0642034 | 18.9389154 |  |
| H | 42.0411107 | 3.1392311 | 16.6227138 | H | 43.1755029 | 5.5373097 | 18.1131607 |  |
| H | 42.8473140 | 4.2326447 | 18.8721111 | H | 44.1849697 | 2.6960674 | 17.5372003 |  |
| H | 43.1961283 | 5.6712389 | 17.9239856 | H | 45.9903781 | 3.6972057 | 16.2126014 |  |
| H | 44.3067620 | 2.8491237 | 17.4602489 | H | 44.4558459 | 4.2966662 | 15.5511665 |  |
| H | 45.8655779 | 3.7602221 | 15.8204770 | H | 45.4548521 | 5.3390415 | 16.6052854 |  |
| H | 44.2456695 | 4.2679263 | 15.3064105 | H | 46.2712017 | 3.3886166 | 18.7436731 |  |
| H | 45.3445848 | 5.4160699 | 16.1387072 | H | 45.5406184 | 4.9635184 | 19.1197392 |  |
| H | 46.4652597 | 3.7436950 | 18.3425472 | H | 44.8530164 | 3.4718786 | 19.8209569 |  |
| H | 45.7235410 | 5.3328226 | 18.6056165 | H | 32.7423667 | 5.5023831 | 15.5160840 |  |
| H | 45.1771781 | 3.9226995 | 19.5619881 | H | 33.1050997 | 5.1626407 | 17.2158954 |  |
| H | 32.7968682 | 5.5952127 | 15.4957794 | H | 31.5183444 | 5.8259443 | 16.7762070 |  |
| H | 33.1719976 | 5.1743327 | 17.1720697 | H | 31.6242132 | 7.8977358 | 18.1261368 |  |
| H | 31.5263644 | 5.6956031 | 16.7479761 | H | 34.7757005 | 6.9890137 | 15.3342455 |  |
| H | 31.2383722 | 7.7700540 | 18.0060302 | H | 32.5300146 | 10.1899248 | 18.4098345 |  |
| H | 34.8168401 | 7.1948471 | 15.6766943 | H | 35.6615341 | 9.2932335 | 15.5853061 |  |
| H | 31.9113801 | 10.1132534 | 18.4668737 | H | 34.5520493 | 10.9109909 | 17.1202638 |  |
| H | 35.4934628 | 9.5320419 | 16.1262201 | H | 30.6912464 | 9.7490428 | 14.4995974 |  |
| H | 34.0457859 | 11.0171500 | 17.5244310 | H | 32.4181033 | 9.6150572 | 14.9079788 |  |
| H | 30.6833933 | 9.7021496 | 14.5096313 | H | 31.9215898 | 10.1718498 | 13.2832190 |  |
| H | 32.4172326 | 9.6532333 | 14.9019537 | H | 30.8985133 | 7.9089450 | 12.8311952 |  |
| H | 31.8782908 | 10.1813818 | 13.2815525 | H | 31.3770760 | 7.3726621 | 14.4612368 |  |
| H | 30.9938335 | 7.8796236 | 12.8156444 | H | 33.7644986 | 8.2620379 | 13.2007296 |  |
| H | 31.4333565 | 7.3656337 | 14.4625859 | H | 32.4473155 | 4.5998459 | 12.0721676 |  |
| H | 33.8410393 | 8.3245233 | 13.2974915 | H | 31.3425730 | 5.6624198 | 12.8698090 |  |
| H | 32.6118637 | 4.7318560 | 11.8625452 | H | 34.6646295 | 5.2274424 | 11.7551167 |  |
| H | 31.4948721 | 5.6785300 | 12.7821125 | H | 35.2242668 | 6.8252244 | 12.0887116 |  |
| H | 34.8018927 | 5.4336727 | 11.6064682 | H | 33.5792128 | 14.5819604 | 13.5534476 |  |
| H | 35.3562664 | 6.9968610 | 12.0834833 | H | 33.1440991 | 14.4486347 | 15.2683511 |  |
| H | 33.4137105 | 14.3191494 | 13.7444122 | H | 33.8416967 | 15.9655781 | 14.6474082 |  |
| H | 33.2228741 | 14.7847216 | 15.4486484 | H | 36.1111633 | 16.2751144 | 15.4198253 |  |
| H | 33.9365356 | 15.9336493 | 14.2949229 | H | 34.8293036 | 12.2863674 | 14.3963090 |  |
| H | 36.1587690 | 16.2192122 | 15.3807411 | H | 38.3905434 | 15.4171743 | 15.9083416 |  |
| H | 34.7572813 | 12.2558894 | 14.4187198 | H | 37.0960108 | 11.4391464 | 14.8739748 |  |
| H | 38.4080941 | 15.2972858 | 15.8913404 | H | 39.8269789 | 13.5708491 | 15.9001767 |  |
| H | 36.9881557 | 11.3460953 | 14.9252039 | H | 34.7437216 | 13.7422525 | 8.8977025 |  |
| H | 39.7531929 | 13.3961630 | 15.9246395 | H | 35.3167584 | 14.6190345 | 10.3325411 |  |
| H | 34.7484124 | 13.7040351 | 8.8940026 | H | 36.4921319 | 13.9294598 | 9.1962854 |  |
| H | 35.3054409 | 14.6441021 | 10.2940407 | H | 37.6235313 | 12.5659617 | 10.9812570 |  |
| H | 36.4936851 | 13.9157616 | 9.1942655 | H | 33.4257431 | 12.0422101 | 10.1524794 |  |
| H | 37.6519878 | 12.5083699 | 10.9118057 | H | 37.6110059 | 10.5014813 | 12.3556099 |  |
| H | 33.3965235 | 12.1522393 | 10.3317839 | H | 33.4170468 | 9.9739386 | 11.5251990 |  |
| H | 37.6243533 | 10.5168239 | 12.3792099 | H | 36.3170502 | 8.8091332 | 13.2232969 |  |
| H | 33.3675244 | 10.1763253 | 11.8244128 | H | 38.9748235 | 6.8867061 | 10.5489639 |  |
| H | 36.1852855 | 9.0878029 | 13.6934032 | H | 37.4298247 | 6.4422890 | 9.7671194 |  |
| H | 38.9298754 | 6.7084598 | 10.5816647 | H | 37.7043354 | 8.1297589 | 10.2938384 |  |
| H | 37.3137018 | 6.3982599 | 9.8865423 | H | 37.7225554 | 7.2286172 | 12.4923029 |  |
| H | 37.8798488 | 8.0797084 | 10.0980881 | H | 43.5122286 | 5.0080652 | 12.5334339 |  |
| H | 37.8185987 | 7.6459681 | 12.4052480 | H | 41.9896575 | 4.0736487 | 12.7356975 |  |
| H | 42.4347607 | 4.6091725 | 11.1155612 | H | 42.5262027 | 4.6344838 | 11.1111866 |  |
| H | 43.5387322 | 5.0080186 | 12.4382309 | H | 40.2982189 | 7.2068063 | 13.3041352 |  |
| H | 42.0334875 | 4.0814389 | 12.7823184 | H | 40.4887764 | 5.5877646 | 13.8759158 |  |
| H | 40.1664796 | 7.1411311 | 13.1865561 | H | 55.3153650 | 3.9024835 | 14.7590972 |  |
| H | 40.3570322 | 5.4833060 | 13.6881123 | H | 53.6288143 | 4.2294594 | 14.2233147 |  |
| H | 55.3107537 | 3.9019897 | 14.7735662 | H | 55.2230770 | 1.7404420 | 13.3727324 |  |
| H | 53.6294638 | 4.2312214 | 14.2275644 | H | 55.0738013 | 3.2363156 | 12.3967501 |  |
| H | 55.2323423 | 1.7512520 | 13.3658741 | H | 51.9877709 | 3.1730109 | 15.3295364 |  |
| H | 55.1042976 | 3.2603426 | 12.4049214 | H | 39.3971939 | 10.5268045 | 14.4713099 |  |
| H | 51.9939476 | 3.2063147 | 15.3605909 | H | 41.6009191 | 9.4435950 | 14.5401934 |  |
| H | 39.7419380 | 10.6636379 | 14.9846880 | H | 40.9354075 | 8.0436160 | 15.4453957 |  |
| H | 41.3671088 | 9.0508878 | 14.3799454 | H | 45.0287993 | 6.9081479 | 15.1294419 |  |
| H | 40.7451997 | 7.8054967 | 15.4829965 | H | 43.3223530 | 6.5967954 | 15.5840559 |  |
| H | 41.6660486 | 8.8388617 | 17.4275716 | H | 43.7020429 | 7.5052045 | 14.0807173 |  |
| H | 41.8239458 | 10.4447116 | 16.6551480 | H | 44.2993304 | 7.5209878 | 17.8972609 |  |
| H | 44.7063072 | 7.1695838 | 15.2444224 | H | 43.9975447 | 9.2335797 | 18.3046169 |  |
| H | 43.0391526 | 6.7573578 | 15.7918562 | H | 46.3138969 | 8.7342414 | 18.8696034 |  |
| H | 43.2991296 | 7.6395412 | 14.2167550 | H | 45.6753941 | 10.7737241 | 16.9136983 |  |
| H | 43.9747488 | 7.6863824 | 17.9917325 | H | 46.7053722 | 9.6697769 | 15.2018456 |  |
| H | 43.8895610 | 9.4472599 | 18.3811622 | H | 48.7327551 | 8.3958212 | 17.1064284 |  |
| H | 46.1582670 | 8.6595640 | 18.7969652 | H | 51.7667239 | 5.1623537 | 16.7739952 |  |
| H | 45.6446911 | 10.8100665 | 16.8828104 | H | 46.7232053 | 7.9123636 | 13.8320352 |  |
| H | 46.6106715 | 9.6122849 | 15.1708899 | H | 39.4541895 | 11.3896602 | 16.5121318 |  |
| H | 48.4194585 | 8.1771594 | 17.1815353 | H | 38.9973845 | 9.9772745 | 17.3257447 |  |
| H | 51.6450544 | 5.1680210 | 16.8436686 | H | 41.1900576 | 9.2483642 | 17.5656620 |  |
| H | 46.57426656 | 7.7481276 | 13.78665036 | H | 41.7472477 | 10.7242224 | 16.7072525 |  |

| ***AzeJ_F134L*** Reactant | ***AzeJ_F134L*** Product | Fixed (f) |
| --- | --- | --- |

| C | 48.8200000 | 9.6360000 | 11.4410000 | C | 48.8200000 | 9.6360000 | 11.4410000 | f |
| --- | --- | --- | --- | --- | --- | --- | --- | --- |
| C | 47.3339556 | 9.6114398 | 11.7308350 | C | 47.3252498 | 9.6204890 | 11.6912980 |  |
| C | 46.7521832 | 10.5339964 | 12.6183955 | C | 46.7244510 | 10.5813346 | 12.5263994 |  |
| C | 46.4835503 | 8.6568343 | 11.1493433 | C | 46.4892248 | 8.6345641 | 11.1433282 |  |
| C | 45.3841349 | 10.5110721 | 12.9078201 | C | 45.3549884 | 10.5626019 | 12.7999171 |  |
| C | 45.1152782 | 8.6088546 | 11.4379838 | C | 45.1156297 | 8.5941248 | 11.4163105 |  |
| C | 44.5507219 | 9.5452694 | 12.3207960 | C | 44.5351028 | 9.5644703 | 12.2486596 |  |
| O | 43.2248875 | 9.5488496 | 12.6398415 | O | 43.2088448 | 9.5690344 | 12.5586555 |  |
| C | 42.1220000 | 15.0210000 | 14.3510000 | C | 42.1220000 | 15.0210000 | 14.3510000 | f |
| C | 41.9720000 | 15.0819802 | 15.8800893 | C | 41.9672738 | 15.0470975 | 15.8806711 |  |
| O | 40.9356230 | 14.7021219 | 16.4423967 | O | 40.9448184 | 14.6107745 | 16.4344819 |  |
| C | 42.7758577 | 13.6853367 | 13.9039091 | C | 42.7368512 | 13.6765693 | 13.8706899 |  |
| C | 42.7602083 | 13.5369380 | 12.3716015 | C | 42.7236633 | 13.5709335 | 12.3349114 |  |
| C | 42.1103605 | 12.4709358 | 14.5663511 | C | 42.0402752 | 12.4612284 | 14.4985205 |  |
| C | 43.5341737 | 14.6110367 | 11.6055591 | C | 43.5401430 | 14.6363165 | 11.6014732 |  |
| N | 43.0444818 | 15.4993674 | 16.5965573 | N | 43.0186127 | 15.5064365 | 16.5994366 |  |
| C | 43.1990000 | 15.3970000 | 18.0630000 | C | 43.1990000 | 15.3970000 | 18.0630000 | f |
| C | 44.5326343 | 14.7271271 | 18.4065099 | C | 44.5126668 | 14.6810729 | 18.3882428 |  |
| C | 44.6653278 | 13.3041004 | 17.8588816 | C | 44.6072648 | 13.2648957 | 17.8166288 |  |
| S | 43.4807644 | 12.1228185 | 18.6161116 | S | 43.3951485 | 12.0838042 | 18.5309007 |  |
| C | 44.4483470 | 11.6532955 | 20.0970703 | C | 44.3355746 | 11.5621558 | 20.0107726 |  |
| C | 51.6880000 | 9.6280000 | 19.1070000 | C | 51.6880000 | 9.6280000 | 19.1070000 | f |
| C | 50.3574445 | 9.8724084 | 18.3678038 | C | 50.3908960 | 10.0751874 | 18.4241558 |  |
| O | 49.2816137 | 9.5977614 | 18.9798993 | O | 49.3060854 | 9.8247493 | 19.0286189 |  |
| O | 50.4270276 | 10.3083527 | 17.1914263 | O | 50.4904446 | 10.6194161 | 17.2963782 |  |
| C | 39.1811000 | 7.5264000 | 20.5701000 | C | 39.1811000 | 7.5264000 | 20.5701000 | f |
| C | 39.1938863 | 6.8683474 | 19.1781100 | C | 39.0220266 | 6.9811053 | 19.1384348 |  |
| O | 39.7403137 | 7.3884698 | 18.2065139 | O | 39.2421560 | 7.6635306 | 18.1354307 |  |
| C | 40.0920822 | 8.7388272 | 20.7610094 | C | 39.9896566 | 8.8194546 | 20.7204877 |  |
| C | 39.7732508 | 10.0191286 | 19.9589348 | C | 39.4732717 | 10.0929360 | 20.0134324 |  |
| C | 38.2766006 | 10.3462810 | 19.8860995 | C | 37.9529920 | 10.2720268 | 20.0961412 |  |
| C | 40.5557042 | 11.1997967 | 20.5453902 | C | 40.2067427 | 11.3255465 | 20.5552708 |  |
| N | 38.5549430 | 5.6568675 | 19.0681334 | N | 38.5968381 | 5.6823515 | 19.0413677 |  |
| C | 38.2620000 | 4.9980000 | 17.7770000 | C | 38.2620000 | 4.9980000 | 17.7770000 | f |
| C | 39.5628796 | 4.8784010 | 16.9158260 | C | 39.5245527 | 4.7349387 | 16.9023749 |  |
| O | 39.5507627 | 5.1396979 | 15.7153072 | O | 39.4050276 | 4.6162824 | 15.6837766 |  |
| C | 37.1901186 | 5.7629642 | 16.9874325 | C | 37.2209074 | 5.7891949 | 16.9634568 |  |
| C | 36.8285791 | 5.0602220 | 15.6809400 | C | 36.5485687 | 4.9782913 | 15.8635063 |  |
| N | 36.6750363 | 5.8748892 | 14.6133433 | N | 36.5364368 | 5.5622264 | 14.6424204 |  |
| O | 36.6432325 | 3.8461244 | 15.6453115 | O | 35.9823385 | 3.9167486 | 16.1135580 |  |
| N | 40.6614762 | 4.4308123 | 17.5622313 | N | 40.7002110 | 4.6147789 | 17.5542033 |  |
| C | 41.9510000 | 4.2010000 | 16.9160000 | C | 41.9510000 | 4.2010000 | 16.9160000 | f |
| C | 43.1280009 | 4.6573104 | 17.7844247 | C | 43.1842641 | 4.7100930 | 17.6611411 |  |
| C | 44.4872016 | 4.0459258 | 17.3889699 | C | 44.4815790 | 3.9480905 | 17.3169122 |  |
| C | 44.9105559 | 4.3533700 | 15.9453041 | C | 44.7430076 | 3.8252155 | 15.8082578 |  |
| C | 45.5867116 | 4.4925439 | 18.3621728 | C | 45.6840911 | 4.5851142 | 18.0271415 |  |
| C | 32.5930000 | 5.8730000 | 16.5430000 | C | 32.5930000 | 5.8730000 | 16.5430000 | f |
| C | 33.0210277 | 7.2964413 | 16.8245454 | C | 32.9265294 | 7.3095636 | 16.8677089 |  |
| C | 32.2276713 | 8.1675687 | 17.5889821 | C | 32.0398567 | 8.1147248 | 17.6016333 |  |
| C | 34.2503470 | 7.7746912 | 16.3389014 | C | 34.1373675 | 7.8799509 | 16.4346972 |  |
| C | 32.6584135 | 9.4713393 | 17.8688283 | C | 32.3534494 | 9.4455534 | 17.9051551 |  |
| C | 34.6955408 | 9.0697366 | 16.6243941 | C | 34.4587321 | 9.2060065 | 16.7420953 |  |
| C | 33.8946211 | 9.9243446 | 17.3928829 | C | 33.5673859 | 9.9948535 | 17.4798434 |  |
| C | 31.6810000 | 9.4790000 | 14.1040000 | C | 31.6810000 | 9.4790000 | 14.1040000 | f |
| C | 31.7177947 | 8.0275980 | 13.6402760 | C | 31.6532914 | 8.0345997 | 13.6253905 |  |
| N | 33.0506041 | 7.6704515 | 13.1591136 | N | 32.9704922 | 7.6152756 | 13.1466098 |  |
| C | 33.3565369 | 6.5172054 | 12.5682728 | C | 33.2128623 | 6.4372870 | 12.5742838 |  |
| N | 32.4326250 | 5.5402699 | 12.4779965 | N | 32.2383127 | 5.5096184 | 12.5076874 |  |
| N | 34.5671820 | 6.3200183 | 12.0346505 | N | 34.4102018 | 6.1612289 | 12.0479935 |  |
| C | 33.8900000 | 14.8690000 | 14.5720000 | C | 33.8900000 | 14.8690000 | 14.5720000 | f |
| C | 35.2461219 | 14.3591585 | 15.0242393 | C | 35.2331551 | 14.3568722 | 15.0599182 |  |
| C | 36.3822602 | 15.1843924 | 15.0073145 | C | 36.3892942 | 15.1543972 | 15.0045770 |  |
| C | 35.4298750 | 13.0280166 | 15.4445841 | C | 35.3857138 | 13.0458694 | 15.5443708 |  |
| C | 37.6462931 | 14.7134111 | 15.3840545 | C | 37.6437832 | 14.6731116 | 15.3996739 |  |
| C | 36.6811311 | 12.5379808 | 15.8278500 | C | 36.6285640 | 12.5475719 | 15.9452114 |  |
| C | 37.8081573 | 13.3792700 | 15.7963222 | C | 37.7722636 | 13.3563589 | 15.8683198 |  |
| O | 39.0096329 | 12.8680071 | 16.1635797 | O | 38.9757927 | 12.8282729 | 16.2427591 |  |
| C | 35.5190000 | 13.7510000 | 9.6800000 | C | 35.5190000 | 13.7510000 | 9.6800000 | f |
| C | 35.4794363 | 12.5270384 | 10.5684004 | C | 35.4509531 | 12.5469467 | 10.5991614 |  |
| C | 36.2993406 | 12.4264685 | 11.7064659 | C | 36.2170791 | 12.4822599 | 11.7761895 |  |
| C | 34.6381861 | 11.4400573 | 10.2799605 | C | 34.6406355 | 11.4374010 | 10.3011221 |  |
| C | 36.2899298 | 11.2931947 | 12.5227377 | C | 36.1854747 | 11.3668406 | 12.6184479 |  |
| C | 34.6160244 | 10.2981683 | 11.0857080 | C | 34.5934523 | 10.3149083 | 11.1342822 |  |
| C | 35.4422103 | 10.2180963 | 12.2163995 | C | 35.3649336 | 10.2730717 | 12.3049356 |  |
| O | 35.3646595 | 9.1070220 | 12.9951327 | O | 35.2688798 | 9.1752831 | 13.1094150 |  |
| C | 37.8850000 | 7.0690000 | 10.5480000 | C | 37.8850000 | 7.0690000 | 10.5480000 | f |
| O | 37.2904487 | 6.9668802 | 11.8265874 | O | 37.0296300 | 6.9164552 | 11.6682532 |  |
| C | 42.4750000 | 4.8880000 | 12.1800000 | C | 42.4750000 | 4.8880000 | 12.1800000 | f |
| C | 41.7735029 | 6.2205735 | 12.3472977 | C | 41.6640522 | 6.1423356 | 12.4457965 |  |
| N | 40.6842461 | 6.2702674 | 13.1229177 | N | 40.5825019 | 6.0244753 | 13.2342382 |  |
| O | 42.2200794 | 7.2264463 | 11.7657911 | O | 41.9983791 | 7.2208869 | 11.9320429 |  |
| C | 54.3690000 | 3.4430000 | 14.4320000 | C | 54.3690000 | 3.4430000 | 14.4320000 | f |
| C | 53.9010415 | 2.5082304 | 15.5811930 | C | 53.9067842 | 2.5251855 | 15.5981924 |  |
| O | 54.7113783 | 1.7772290 | 16.1564854 | O | 54.7252658 | 1.8282887 | 16.2044401 |  |
| C | 54.6584548 | 2.6207213 | 13.1650565 | C | 54.6131284 | 2.6030603 | 13.1659447 |  |
| C | 53.3438713 | 2.1297945 | 12.5105876 | C | 53.2766651 | 2.0999618 | 12.5640955 |  |
| O | 52.5585576 | 3.0683523 | 12.1624279 | O | 52.4813716 | 3.0301025 | 12.2196660 |  |
| O | 53.1539934 | 0.9088069 | 12.3852920 | O | 53.0839207 | 0.8755081 | 12.4753982 |  |
| N | 52.5739072 | 2.5279294 | 15.8737360 | N | 52.5749152 | 2.5176101 | 15.8662484 |  |
| C | 51.9070000 | 1.7130000 | 16.8920000 | C | 51.9070000 | 1.7130000 | 16.8920000 | f |
| N | 38.8159767 | 9.8500857 | 16.6466543 | N | 39.4573577 | 10.0609994 | 16.4230442 |  |
| C | 39.3894191 | 9.6753910 | 15.3354247 | C | 39.4393530 | 9.5806059 | 14.9863237 |  |
| C | 38.4430958 | 8.9246458 | 14.3735525 | C | 38.1953539 | 8.8117057 | 14.5707287 |  |
| O | 37.2310823 | 8.7833667 | 14.7049734 | O | 37.0995078 | 9.2536731 | 14.9884307 |  |
| C | 40.7902456 | 8.9988066 | 15.2664456 | C | 40.7887617 | 8.8810073 | 15.1862851 |  |
| C | 41.7110731 | 9.4334965 | 16.4057535 | C | 40.9351560 | 9.6655885 | 16.4982680 |  |
| S | 43.4918401 | 9.1812406 | 15.9770050 | S | 43.9841786 | 9.0527505 | 15.7861476 |  |
| C | 43.5240279 | 7.5662936 | 15.1594900 | C | 44.0357387 | 7.3910371 | 15.0501825 |  |
| C | 44.2134113 | 8.7769590 | 17.6243443 | C | 44.5442953 | 8.7293836 | 17.5019895 |  |
| C | 45.7326129 | 8.8249989 | 17.6503615 | C | 46.0396492 | 8.9354072 | 17.6887686 |  |
| O | 46.2545669 | 7.7586737 | 16.8556045 | O | 46.7247729 | 7.8874189 | 16.9951074 |  |
| C | 46.4078929 | 10.1507479 | 17.1609259 | C | 46.5798508 | 10.2914621 | 17.1451028 |  |
| O | 47.1129197 | 10.8123000 | 18.1639708 | O | 47.2219958 | 11.0711764 | 18.1124992 |  |
| C | 47.2668880 | 9.6475759 | 15.9620101 | C | 47.4664341 | 9.8429429 | 15.9451307 |  |
| O | 48.3554244 | 10.4084205 | 15.5986939 | O | 48.4959795 | 10.6907453 | 15.5692672 |  |
| C | 47.5368268 | 8.1851664 | 16.3675879 | C | 47.8817090 | 8.4187927 | 16.3608305 |  |
| O | 38.9268461 | 8.5017385 | 13.2909329 | O | 38.3762829 | 7.8294948 | 13.8080111 |  |
| N | 50.9463299 | 4.6423253 | 14.9907592 | N | 50.9930969 | 4.6307760 | 14.9466506 |  |
| C | 50.8071836 | 5.3541993 | 16.1175048 | C | 50.9891831 | 5.4208968 | 16.0289536 |  |
| N | 49.8819457 | 6.2652800 | 16.4089385 | N | 50.1638828 | 6.4249001 | 16.3114622 |  |
| C | 49.0247887 | 6.4292918 | 15.3926268 | C | 49.2520957 | 6.5925511 | 15.3426041 |  |
| C | 49.0371104 | 5.7546166 | 14.1653391 | C | 49.1296007 | 5.8411675 | 14.1647878 |  |
| C | 50.0810000 | 4.7970000 | 13.9630000 | C | 50.0810000 | 4.7970000 | 13.9630000 | f |
| N | 50.2065385 | 4.0789592 | 12.8473020 | N | 50.0908246 | 3.9989079 | 12.8897964 |  |
| N | 48.0038170 | 6.1969556 | 13.3583358 | N | 48.0695299 | 6.3018912 | 13.4050443 |  |
| C | 47.3893726 | 7.1118907 | 14.0678258 | C | 47.5670702 | 7.2948216 | 14.1003206 |  |
| N | 47.9559400 | 7.3042611 | 15.3185465 | N | 48.2450200 | 7.5341541 | 15.2830180 |  |
| H | 39.1076128 | 10.7247123 | 17.0722288 | H | 49.4498097 | 4.2425042 | 12.1430182 |  |
| H | 38.9863251 | 9.0584419 | 17.2627468 | H | 50.9695151 | 3.4702571 | 12.6434370 |  |
| H | 49.5678133 | 4.2978776 | 12.0911311 | H | 49.1948943 | 10.7187142 | 16.2884531 |  |
| H | 51.0988546 | 3.5606186 | 12.6195747 | H | 48.0432666 | 10.5874666 | 18.4582225 |  |
| H | 49.0932315 | 10.3726045 | 16.2888641 | H | 51.2544482 | 2.3496230 | 17.5125946 |  |
| H | 47.9534993 | 10.3081374 | 18.4338103 | H | 51.2900302 | 0.9173299 | 16.4382261 |  |
| H | 51.3345988 | 2.3511776 | 17.5865163 | H | 52.6708834 | 1.2431837 | 17.5288264 |  |
| H | 51.2124106 | 0.9891670 | 16.4308288 | H | 49.1739845 | 8.6607459 | 11.0742041 |  |
| H | 52.6707351 | 1.1578963 | 17.4558600 | H | 49.1011683 | 10.4033649 | 10.6981604 |  |
| H | 49.1644371 | 8.6704983 | 11.0409343 | H | 49.3594509 | 9.8658598 | 12.3747945 |  |
| H | 49.0772608 | 10.4201876 | 10.7072929 | H | 47.3529495 | 11.3255881 | 13.0241474 |  |
| H | 49.3867698 | 9.8453269 | 12.3628876 | H | 46.9244939 | 7.8497555 | 10.5179772 |  |
| H | 47.3896761 | 11.2537951 | 13.1389987 | H | 44.9121670 | 11.2906623 | 13.4818149 |  |
| H | 46.9045119 | 7.9027762 | 10.4781783 | H | 44.4839913 | 7.8023646 | 11.0041274 |  |
| H | 44.9520736 | 11.2285583 | 13.6091704 | H | 42.7631184 | 8.7512889 | 12.2380787 |  |
| H | 44.4750140 | 7.8411366 | 10.9983296 | H | 42.7361910 | 15.8684884 | 14.0111766 |  |
| H | 42.7631424 | 8.7800498 | 12.2204064 | H | 41.1118143 | 15.1346133 | 13.9267911 |  |
| H | 42.7106087 | 15.8779846 | 13.9890369 | H | 43.7951025 | 13.6617961 | 14.1962651 |  |
| H | 41.1076297 | 15.0948505 | 13.9294622 | H | 41.6724708 | 13.5941186 | 11.9898070 |  |
| H | 43.8357157 | 13.7115483 | 14.2246771 | H | 43.1076235 | 12.5720962 | 12.0658193 |  |
| H | 41.7078094 | 13.5134167 | 12.0318023 | H | 42.4305406 | 11.5251876 | 14.0709267 |  |
| H | 43.1770627 | 12.5450858 | 12.1261111 | H | 42.1931766 | 12.4271315 | 15.5876296 |  |
| H | 42.5206886 | 11.5351303 | 14.1574402 | H | 40.9515334 | 12.4996718 | 14.3230566 |  |
| H | 42.2594149 | 12.4703672 | 15.6576317 | H | 43.5508931 | 14.4444345 | 10.5165229 |  |
| H | 41.0221318 | 12.4724308 | 14.3923551 | H | 43.1362272 | 15.6516243 | 11.7495579 |  |
| H | 43.5483440 | 14.3897505 | 10.5261971 | H | 44.5876814 | 14.6377654 | 11.9484479 |  |
| H | 43.0924738 | 15.6140054 | 11.7274412 | H | 43.8105312 | 15.8669638 | 16.0750032 |  |
| H | 44.5821618 | 14.6622873 | 11.9478176 | H | 42.3381575 | 14.8325617 | 18.4484137 |  |
| H | 43.8504230 | 15.8335260 | 16.0761665 | H | 43.1833020 | 16.4035574 | 18.5123886 |  |
| H | 42.3496058 | 14.8067157 | 18.4343174 | H | 44.6395527 | 14.6598347 | 19.4837601 |  |
| H | 43.1438436 | 16.4007919 | 18.5162394 | H | 45.3603763 | 15.2722470 | 17.9937235 |  |
| H | 44.6582665 | 14.7281213 | 19.5022107 | H | 45.6129579 | 12.8409224 | 17.9624229 |  |
| H | 45.3639870 | 15.3350490 | 18.0031595 | H | 44.4119900 | 13.2715662 | 16.7328250 |  |
| H | 45.6799721 | 12.9018034 | 18.0044971 | H | 43.7865163 | 10.7290817 | 20.4741921 |  |
| H | 44.4583230 | 13.2863141 | 16.7772225 | H | 45.3394976 | 11.2267718 | 19.7061704 |  |
| H | 43.8943352 | 10.8600297 | 20.6195926 | H | 44.4183563 | 12.3813433 | 20.7415047 |  |
| H | 45.4354247 | 11.2791771 | 19.7839355 | H | 52.5170538 | 10.3024740 | 18.8467405 |  |
| H | 44.5630131 | 12.5090218 | 20.7793060 | H | 51.5606209 | 9.5652575 | 20.1974929 |  |
| H | 52.4330516 | 10.3798536 | 18.8083412 | H | 51.9385372 | 8.6219563 | 18.7283303 |  |
| H | 51.5405347 | 9.6324172 | 20.1966052 | H | 39.6329765 | 6.7356899 | 21.1927607 |  |
| H | 52.0698900 | 8.6364614 | 18.8095251 | H | 38.1584600 | 7.6614736 | 20.9653378 |  |
| H | 39.4398162 | 6.7488812 | 21.3101620 | H | 41.0293577 | 8.6384366 | 20.3964386 |  |
| H | 38.1303899 | 7.7902381 | 20.7840434 | H | 40.0446657 | 9.0257320 | 21.8043654 |  |
| H | 41.1365074 | 8.4454206 | 20.5552623 | H | 37.4129121 | 9.4625547 | 19.5784460 |  |
| H | 40.0537787 | 8.9883773 | 21.8367811 | H | 38.3778619 | 5.1944358 | 19.9032982 |  |
| H | 37.7087551 | 9.5905608 | 19.3218050 | H | 37.8360109 | 4.0174517 | 18.0385140 |  |
| H | 38.0605652 | 5.3084853 | 19.8828634 | H | 37.6738943 | 6.7056771 | 16.5710196 |  |
| H | 37.9116289 | 3.9775331 | 17.9881472 | H | 36.4140724 | 6.0866484 | 17.6522725 |  |
| H | 37.5256896 | 6.7935497 | 16.8070503 | H | 36.1272635 | 5.0481033 | 13.8696568 |  |
| H | 36.2753768 | 5.8111852 | 17.6007527 | H | 37.1786590 | 6.3200057 | 14.4073568 |  |
| H | 36.5658229 | 5.4688973 | 13.6905309 | H | 40.6835130 | 4.6875656 | 18.5660429 |  |
| H | 36.9388695 | 6.8616990 | 14.6526697 | H | 41.9276149 | 4.5680014 | 15.8794741 |  |
| H | 40.5852538 | 4.2440881 | 18.5568907 | H | 41.9795523 | 3.0963690 | 16.8573739 |  |
| H | 41.9223821 | 4.7235306 | 15.9497981 | H | 43.0144851 | 4.6158968 | 18.7496033 |  |
| H | 42.0620783 | 3.1242413 | 16.6870603 | H | 43.3120207 | 5.7877204 | 17.4652357 |  |
| H | 42.9224509 | 4.3726051 | 18.8320121 | H | 44.3551372 | 2.9208233 | 17.7111586 |  |
| H | 43.1843451 | 5.7607360 | 17.7847467 | H | 45.6725866 | 3.2628642 | 15.6257263 |  |
| H | 44.3710313 | 2.9487479 | 17.4795038 | H | 43.9310730 | 3.2982554 | 15.2817389 |  |
| H | 45.8046558 | 3.7704254 | 15.6727478 | H | 44.8650806 | 4.8135782 | 15.3402642 |  |
| H | 44.1263726 | 4.1146267 | 15.2082846 | H | 46.5921437 | 3.9778598 | 17.8807760 |  |
| H | 45.1830270 | 5.4137716 | 15.8411369 | H | 45.9008256 | 5.5937527 | 17.6361414 |  |
| H | 46.5386460 | 3.9840073 | 18.1392004 | H | 45.5101984 | 4.6722999 | 19.1132677 |  |
| H | 45.7710595 | 5.5759686 | 18.2679885 | H | 32.3935844 | 5.7412681 | 15.4652929 |  |
| H | 45.3208451 | 4.2700311 | 19.4091930 | H | 33.4372297 | 5.2021686 | 16.7756501 |  |
| H | 32.7875531 | 5.5954112 | 15.4937945 | H | 31.7066367 | 5.5229190 | 17.0927918 |  |
| H | 33.1614300 | 5.1589697 | 17.1643596 | H | 31.0907168 | 7.6922920 | 17.9457750 |  |
| H | 31.5232132 | 5.7163730 | 16.7511424 | H | 34.8388237 | 7.2833424 | 15.8449603 |  |
| H | 31.2611900 | 7.8233172 | 17.9705025 | H | 31.6465200 | 10.0521872 | 18.4782349 |  |
| H | 34.8686039 | 7.1219876 | 15.7208132 | H | 35.4066108 | 9.6108363 | 16.3882524 |  |
| H | 32.0230896 | 10.1350379 | 18.4626533 | H | 33.8195347 | 11.0316148 | 17.7191196 |  |
| H | 35.6675495 | 9.3905056 | 16.2413484 | H | 30.6775954 | 9.7780849 | 14.4401801 |  |
| H | 34.2344020 | 10.9395981 | 17.6151801 | H | 32.3749394 | 9.5984722 | 14.9481958 |  |
| H | 30.6674096 | 9.7373260 | 14.4437832 | H | 31.9928437 | 10.1571605 | 13.2930202 |  |
| H | 32.3754669 | 9.6359335 | 14.9430982 | H | 30.9183842 | 7.9181958 | 12.8084388 |  |
| H | 31.9594915 | 10.1603141 | 13.2838237 | H | 31.3281299 | 7.3837466 | 14.4562014 |  |
| H | 30.9858188 | 7.8701297 | 12.8274096 | H | 33.7701019 | 8.2659112 | 13.2251640 |  |
| H | 31.4278665 | 7.3681634 | 14.4775022 | H | 32.4133516 | 4.6027112 | 12.0930680 |  |
| H | 33.8301723 | 8.3442479 | 13.2492147 | H | 31.3567435 | 5.6360457 | 12.9867087 |  |
| H | 32.6581940 | 4.6516454 | 12.0488012 | H | 34.5773753 | 5.2492783 | 11.6404115 |  |
| H | 31.5409236 | 5.6149081 | 12.9487455 | H | 35.2088921 | 6.8124181 | 11.9949789 |  |
| H | 34.8075550 | 5.4066470 | 11.6696642 | H | 33.7253516 | 14.5833033 | 13.5175565 |  |
| H | 35.3466293 | 6.9913114 | 12.0914977 | H | 33.0597971 | 14.4442968 | 15.1585861 |  |
| H | 33.6819270 | 14.5634580 | 13.5306785 | H | 33.8298821 | 15.9667568 | 14.6321764 |  |
| H | 33.0771706 | 14.4628663 | 15.1962002 | H | 36.3146161 | 16.1834660 | 14.6393819 |  |
| H | 33.8372572 | 15.9681877 | 14.6125895 | H | 34.5161142 | 12.3834220 | 15.5939037 |  |
| H | 36.2831775 | 16.2283053 | 14.6918798 | H | 38.5284312 | 15.3130343 | 15.3552154 |  |
| H | 34.5729309 | 12.3467540 | 15.4635606 | H | 36.7259763 | 11.5131307 | 16.2793022 |  |
| H | 38.5150032 | 15.3768147 | 15.3738228 | H | 39.7061125 | 13.5088860 | 16.2489611 |  |
| H | 36.8217782 | 11.4994484 | 16.1395056 | H | 34.6197021 | 13.8297517 | 9.0492295 |  |
| H | 39.7075570 | 13.5689168 | 16.1935175 | H | 35.6197244 | 14.6872620 | 10.2523665 |  |
| H | 34.6361421 | 13.8050934 | 9.0241053 | H | 36.3909996 | 13.6885860 | 9.0048086 |  |
| H | 35.5587479 | 14.6782996 | 10.2751575 | H | 36.8488461 | 13.3300340 | 12.0587727 |  |
| H | 36.4131655 | 13.7476965 | 9.0314339 | H | 34.0271137 | 11.4482409 | 9.3945664 |  |
| H | 36.9519698 | 13.2617851 | 11.9791283 | H | 36.7681360 | 11.3521259 | 13.5393669 |  |
| H | 33.9832487 | 11.4845656 | 9.4038303 | H | 33.9569204 | 9.4617500 | 10.8864007 |  |
| H | 36.9057260 | 11.2535511 | 13.4218654 | H | 35.9237685 | 9.1911154 | 13.8707296 |  |
| H | 33.9593961 | 9.4571123 | 10.8489545 | H | 38.9035063 | 6.6904504 | 10.7550603 |  |
| H | 36.1276173 | 9.0122905 | 13.6612966 | H | 37.4680577 | 6.4892650 | 9.7087487 |  |
| H | 38.9458580 | 6.7521707 | 10.5569922 | H | 37.9701860 | 8.1242262 | 10.2239530 |  |
| H | 37.3409586 | 6.4103494 | 9.8499720 | H | 37.4519938 | 7.3518225 | 12.4429372 |  |
| H | 37.8412650 | 8.0993037 | 10.1438014 | H | 42.5555740 | 4.7513426 | 11.0912734 |  |
| H | 37.8155691 | 7.5617828 | 12.4263227 | H | 43.4964665 | 5.0428638 | 12.5635482 |  |
| H | 42.5073918 | 4.6461099 | 11.1068071 | H | 42.0483819 | 3.9834868 | 12.6359413 |  |
| H | 43.5185306 | 4.9930950 | 12.5170543 | H | 39.9712064 | 6.8300639 | 13.4022267 |  |
| H | 41.9913639 | 4.0662584 | 12.7271037 | H | 40.3462627 | 5.1728491 | 13.7369961 |  |
| H | 40.1565279 | 7.1515689 | 13.1928041 | H | 55.3017715 | 3.9216215 | 14.7711616 |  |
| H | 40.3573256 | 5.4905337 | 13.6878317 | H | 53.6175662 | 4.2136011 | 14.2064275 |  |
| H | 55.2854486 | 3.9355352 | 14.7959620 | H | 55.2692937 | 1.7489564 | 13.3962190 |  |
| H | 53.6111689 | 4.2047828 | 14.1979982 | H | 55.1135223 | 3.2361448 | 12.4118751 |  |
| H | 55.3064373 | 1.7640162 | 13.4072720 | H | 51.9853120 | 3.1758344 | 15.3300222 |  |
| H | 55.1841300 | 3.2636752 | 12.4371194 | H | 39.5156149 | 10.4652944 | 14.3352054 |  |
| H | 51.9892814 | 3.2065345 | 15.3604291 | H | 41.5441568 | 9.0637660 | 14.4168380 |  |
| H | 39.4841296 | 10.6835279 | 14.8956670 | H | 40.6755074 | 7.8026583 | 15.3536052 |  |
| H | 41.2317491 | 9.2263848 | 14.2859659 | H | 41.1497989 | 9.0971383 | 17.4075456 |  |
| H | 40.6689394 | 7.9076053 | 15.3258034 | H | 41.5623160 | 10.5593382 | 16.4405163 |  |
| H | 41.5093715 | 8.8639181 | 17.3248947 | H | 45.0118602 | 6.9294128 | 15.2383938 |  |
| H | 41.6576100 | 10.5080260 | 16.6274381 | H | 43.2319852 | 6.7507762 | 15.4424478 |  |
| H | 44.5762234 | 7.2640525 | 15.1739099 | H | 43.8853980 | 7.5227763 | 13.9714751 |  |
| H | 42.9030306 | 6.8497235 | 15.7089832 | H | 44.2542321 | 7.7094836 | 17.7953035 |  |
| H | 43.1708517 | 7.7222675 | 14.1365605 | H | 43.9934392 | 9.4424241 | 18.1300665 |  |
| H | 43.8248935 | 7.7903513 | 17.9161942 | H | 46.2829094 | 8.8802320 | 18.7670301 |  |
| H | 43.8063412 | 9.5571161 | 18.2800389 | H | 45.7451587 | 10.8967026 | 16.7591451 |  |
| H | 46.0263878 | 8.6833384 | 18.7076100 | H | 46.7826034 | 9.7367385 | 15.0888133 |  |
| H | 45.6422113 | 10.8553907 | 16.7962267 | H | 48.7343631 | 8.4401494 | 17.0577740 |  |
| H | 46.5856693 | 9.5972962 | 15.0947297 | H | 51.7680768 | 5.1958198 | 16.7687813 |  |
| H | 48.2976099 | 8.1267744 | 17.1661309 | H | 46.7151595 | 7.9015869 | 13.8013772 |  |
| H | 51.5562935 | 5.1453373 | 16.8919216 | H | 37.6478050 | 11.2224410 | 19.6270933 |  |
| H | 46.5354519 | 7.6975779 | 13.7304149 | H | 37.6072889 | 10.2943259 | 21.1443802 |  |
| H | 38.1180378 | 11.3082577 | 19.3722929 | H | 41.2992595 | 11.1972593 | 20.5072142 |  |
| H | 37.8365619 | 10.4281317 | 20.8960468 | H | 39.9332673 | 11.5155768 | 21.6080253 |  |
| H | 41.6219106 | 10.9540039 | 20.6666961 | H | 39.7358713 | 9.9983201 | 18.9516231 |  |
| H | 40.1646539 | 11.4748807 | 21.5411405 | H | 39.9599290 | 12.2252418 | 19.9691436 |  |
| H | 40.1263791 | 9.8600015 | 18.9303276 | H | 38.8921225 | 9.4591594 | 17.0404088 |  |
| H | 40.4963294 | 12.0864360 | 19.8949642 | H | 39.2293630 | 11.0711357 | 16.5516014 |  |

| ***AzeJ_L136D*** Reactant | ***AzeJ_L136D*** Product | Fixed (f) |
| --- | --- | --- |

| C | 48.8200000 | 9.6360000 | 11.4410000 | C | 48.8200000 | 9.6360000 | 11.4410000 | f |
| --- | --- | --- | --- | --- | --- | --- | --- | --- |
| C | 47.3247321 | 9.6356751 | 11.6692247 | C | 47.3261204 | 9.6228054 | 11.6826318 |  |
| C | 46.7220093 | 10.5832997 | 12.5143555 | C | 46.7211388 | 10.5635166 | 12.5363692 |  |
| C | 46.4909069 | 8.6605782 | 11.0983970 | C | 46.4989175 | 8.6361788 | 11.1239798 |  |
| C | 45.3530657 | 10.5489964 | 12.7949867 | C | 45.3562165 | 10.5148689 | 12.8303083 |  |
| C | 45.1210568 | 8.6050521 | 11.3742349 | C | 45.1308767 | 8.5709893 | 11.4090536 |  |
| C | 44.5378253 | 9.5509328 | 12.2357764 | C | 44.5449272 | 9.5125660 | 12.2723222 |  |
| O | 43.2178200 | 9.5316678 | 12.5620391 | O | 43.2258376 | 9.4855234 | 12.5968274 |  |
| C | 42.1220000 | 15.0210000 | 14.3510000 | C | 42.1220000 | 15.0210000 | 14.3510000 | f |
| C | 41.9686776 | 15.0512334 | 15.8824374 | C | 41.9652688 | 15.0078620 | 15.8819746 |  |
| O | 40.9497401 | 14.6170990 | 16.4400990 | O | 40.9698968 | 14.5008841 | 16.4273345 |  |
| C | 42.8259990 | 13.7185856 | 13.8792339 | C | 42.8263288 | 13.7336913 | 13.8411941 |  |
| C | 42.8744482 | 13.6368463 | 12.3422629 | C | 42.9289436 | 13.7223164 | 12.3045333 |  |
| C | 42.1641068 | 12.4621274 | 14.4615526 | C | 42.1298216 | 12.4601559 | 14.3396567 |  |
| C | 43.6742509 | 14.7450241 | 11.6550284 | C | 43.7334893 | 14.8702709 | 11.6919194 |  |
| N | 43.0223449 | 15.5125971 | 16.6005834 | N | 42.9881652 | 15.5236254 | 16.6049400 |  |
| C | 43.1990000 | 15.3970000 | 18.0630000 | C | 43.1990000 | 15.3970000 | 18.0630000 | f |
| C | 44.5415199 | 14.7355423 | 18.3871869 | C | 44.5378571 | 14.7105340 | 18.3540707 |  |
| C | 44.7009256 | 13.3383372 | 17.7853266 | C | 44.6816252 | 13.3196574 | 17.7310781 |  |
| S | 43.4919588 | 12.1177025 | 18.4271416 | S | 43.5211474 | 12.0586756 | 18.3878842 |  |
| C | 44.3683757 | 11.5924731 | 19.9438085 | C | 44.4445116 | 11.5452351 | 19.8797369 |  |
| C | 51.6880000 | 9.6280000 | 19.1070000 | C | 51.6880000 | 9.6280000 | 19.1070000 | f |
| C | 50.4022349 | 10.0404027 | 18.3636231 | C | 50.4477388 | 10.2790187 | 18.4819464 |  |
| O | 49.2986640 | 9.8413482 | 18.9511779 | O | 49.3724036 | 10.2095485 | 19.1436343 |  |
| O | 50.5361538 | 10.5180076 | 17.2087476 | O | 50.5766680 | 10.7839291 | 17.3374135 |  |
| C | 39.1800000 | 7.5290000 | 20.5670000 | C | 39.1800000 | 7.5290000 | 20.5670000 | f |
| C | 39.2240800 | 6.8406275 | 19.1862927 | C | 39.0175199 | 6.9373443 | 19.1530927 |  |
| O | 39.8495427 | 7.3192446 | 18.2450986 | O | 39.3034814 | 7.5828570 | 18.1447655 |  |
| C | 39.9577783 | 8.8417766 | 20.6288006 | C | 39.9384044 | 8.8563231 | 20.5730862 |  |
| C | 39.3398285 | 10.0590504 | 19.9618718 | C | 39.2776457 | 10.0441705 | 19.8945990 |  |
| C | 37.9634287 | 10.1962965 | 19.7322588 | C | 37.9054658 | 10.0978971 | 19.6025275 |  |
| C | 40.1778293 | 11.1259305 | 19.5901623 | C | 40.0682791 | 11.1580158 | 19.5561805 |  |
| C | 37.4419985 | 11.3639123 | 19.1613764 | C | 37.3401023 | 11.2327239 | 19.0067969 |  |
| C | 39.6664220 | 12.2897283 | 19.0119069 | C | 39.5114478 | 12.2922405 | 18.9584554 |  |
| C | 38.2871615 | 12.4155930 | 18.8018055 | C | 38.1368154 | 12.3371393 | 18.6903465 |  |
| N | 38.5568168 | 5.6480424 | 19.0696992 | N | 38.5429922 | 5.6615942 | 19.0635764 |  |
| C | 38.2620000 | 4.9980000 | 17.7770000 | C | 38.2620000 | 4.9980000 | 17.7770000 | f |
| C | 39.5483397 | 4.8615999 | 16.8860659 | C | 39.5526634 | 4.9153641 | 16.8936530 |  |
| O | 39.5699967 | 5.3220594 | 15.7456735 | O | 39.5435926 | 5.3127264 | 15.7278306 |  |
| C | 37.1993892 | 5.7836275 | 16.9952837 | C | 37.1420145 | 5.7201973 | 17.0120050 |  |
| C | 36.8551586 | 5.1014726 | 15.6743352 | C | 36.8415542 | 5.0744077 | 15.6633054 |  |
| N | 36.7252200 | 5.9289251 | 14.6132779 | N | 36.6896014 | 5.9478543 | 14.6339159 |  |
| O | 36.6629364 | 3.8881589 | 15.6187222 | O | 36.6829716 | 3.8634215 | 15.5522157 |  |
| N | 40.5687701 | 4.1690330 | 17.4328341 | N | 40.6072487 | 4.3393536 | 17.4942153 |  |
| C | 41.9510000 | 4.2010000 | 16.9160000 | C | 41.9510000 | 4.2010000 | 16.9160000 | f |
| C | 42.8162705 | 5.0515593 | 17.8318238 | C | 42.9857246 | 5.0188875 | 17.6702847 |  |
| C | 44.2959878 | 5.1899541 | 17.3899160 | C | 44.4346407 | 4.7563817 | 17.1640611 |  |
| O | 44.5875120 | 4.7506100 | 16.2536452 | O | 44.5369703 | 4.0523013 | 16.1324760 |  |
| O | 45.0258428 | 5.7906360 | 18.2145340 | O | 45.3396898 | 5.3097643 | 17.8274220 |  |
| C | 32.5930000 | 5.8730000 | 16.5430000 | C | 32.5930000 | 5.8730000 | 16.5430000 | f |
| C | 33.0495067 | 7.2934083 | 16.8047039 | C | 32.9756496 | 7.3091777 | 16.8258557 |  |
| C | 32.2640673 | 8.1972615 | 17.5389164 | C | 32.1228477 | 8.1710107 | 17.5352116 |  |
| C | 34.2975057 | 7.7349047 | 16.3311597 | C | 34.2153115 | 7.8116382 | 16.3932013 |  |
| C | 32.7190778 | 9.4977048 | 17.7970854 | C | 32.5002819 | 9.4921119 | 17.8091428 |  |
| C | 34.7703328 | 9.0239063 | 16.6005168 | C | 34.6038799 | 9.1258264 | 16.6726913 |  |
| C | 33.9731938 | 9.9128704 | 17.3331314 | C | 33.7425209 | 9.9734643 | 17.3801505 |  |
| C | 31.6810000 | 9.4790000 | 14.1040000 | C | 31.6810000 | 9.4790000 | 14.1040000 | f |
| C | 31.7653322 | 8.0255821 | 13.6537076 | C | 31.7456928 | 8.0241537 | 13.6601106 |  |
| N | 33.1097411 | 7.6909330 | 13.1882518 | N | 33.0818101 | 7.6657662 | 13.1837462 |  |
| C | 33.4282420 | 6.5500968 | 12.5791005 | C | 33.3650150 | 6.5075591 | 12.5852363 |  |
| N | 32.5046879 | 5.5734100 | 12.4576843 | N | 32.4182600 | 5.5504328 | 12.5019620 |  |
| N | 34.6419975 | 6.3653321 | 12.0527862 | N | 34.5596545 | 6.2859125 | 12.0358954 |  |
| C | 33.8900000 | 14.8690000 | 14.5720000 | C | 33.8900000 | 14.8690000 | 14.5720000 | f |
| C | 35.2710460 | 14.3355366 | 14.9016428 | C | 35.2683042 | 14.3239084 | 14.8992452 |  |
| C | 36.3472661 | 15.1964657 | 15.1627986 | C | 36.3639291 | 15.1691546 | 15.1363878 |  |
| C | 35.5310743 | 12.9505322 | 14.9452381 | C | 35.5051780 | 12.9373556 | 14.9614724 |  |
| C | 37.6315201 | 14.7106760 | 15.4419093 | C | 37.6430089 | 14.6666891 | 15.4139653 |  |
| C | 36.8001992 | 12.4454442 | 15.2292901 | C | 36.7702296 | 12.4182681 | 15.2390572 |  |
| C | 37.8726456 | 13.3265605 | 15.4663322 | C | 37.8579606 | 13.2806844 | 15.4577407 |  |
| O | 39.0949668 | 12.8046707 | 15.7068636 | O | 39.0783691 | 12.7348210 | 15.7070856 |  |
| C | 35.5190000 | 13.7510000 | 9.6800000 | C | 35.5190000 | 13.7510000 | 9.6800000 | f |
| C | 35.5198440 | 12.5102572 | 10.5401734 | C | 35.5217702 | 12.5263631 | 10.5698834 |  |
| C | 36.6744237 | 12.0942732 | 11.2247553 | C | 36.6715351 | 12.1319240 | 11.2756895 |  |
| C | 34.3580409 | 11.7426184 | 10.7090327 | C | 34.3655511 | 11.7503439 | 10.7464755 |  |
| C | 36.6738459 | 10.9806607 | 12.0666231 | C | 36.6686351 | 11.0372058 | 12.1447053 |  |
| C | 34.3382119 | 10.6279250 | 11.5489827 | C | 34.3448226 | 10.6557955 | 11.6140847 |  |
| C | 35.4894144 | 10.2512132 | 12.2579508 | C | 35.4892339 | 10.3053318 | 12.3453244 |  |
| O | 35.4060760 | 9.1934482 | 13.1029247 | O | 35.3953121 | 9.2618809 | 13.2202535 |  |
| C | 37.8850000 | 7.0690000 | 10.5480000 | C | 37.8850000 | 7.0690000 | 10.5480000 | f |
| O | 37.3084765 | 7.0870559 | 11.8379680 | O | 37.1637127 | 7.0391600 | 11.7680708 |  |
| C | 42.4750000 | 4.8880000 | 12.1800000 | C | 42.4750000 | 4.8880000 | 12.1800000 | f |
| C | 41.7765219 | 6.2264398 | 12.2731428 | C | 41.7665579 | 6.2226239 | 12.2715877 |  |
| N | 40.6792019 | 6.3048154 | 13.0356574 | N | 40.7034768 | 6.3056915 | 13.0879432 |  |
| O | 42.2331535 | 7.2133691 | 11.6662652 | O | 42.1660527 | 7.1977584 | 11.6125460 |  |
| C | 54.3690000 | 3.4430000 | 14.4320000 | C | 54.3690000 | 3.4430000 | 14.4320000 | f |
| C | 53.9009558 | 2.5165198 | 15.5880456 | C | 53.9091434 | 2.5313533 | 15.6041157 |  |
| O | 54.7154872 | 1.7971459 | 16.1748497 | O | 54.7355987 | 1.8623463 | 16.2335267 |  |
| C | 54.6773132 | 2.6074556 | 13.1795982 | C | 54.6389219 | 2.5967069 | 13.1770008 |  |
| C | 53.3871752 | 2.0554946 | 12.5159610 | C | 53.3322105 | 2.0515107 | 12.5363431 |  |
| O | 52.5106738 | 2.9360619 | 12.2586484 | O | 52.4462428 | 2.9304743 | 12.3149745 |  |
| O | 53.3180408 | 0.8355711 | 12.2817865 | O | 53.2669657 | 0.8346320 | 12.2812605 |  |
| N | 52.5725671 | 2.5260323 | 15.8702933 | N | 52.5743150 | 2.4954570 | 15.8481123 |  |
| C | 51.9070000 | 1.7130000 | 16.8920000 | C | 51.9070000 | 1.7130000 | 16.8920000 | f |
| N | 38.9884266 | 9.7425407 | 16.6434957 | N | 39.6143123 | 9.9619531 | 16.5460751 |  |
| C | 39.5635565 | 9.5829096 | 15.3343814 | C | 39.6933277 | 9.6296993 | 15.0783200 |  |
| C | 38.5801148 | 8.9314655 | 14.3428103 | C | 38.5082188 | 8.9548261 | 14.4270006 |  |
| O | 37.3671502 | 8.7894741 | 14.6785109 | O | 37.3830160 | 8.9690476 | 14.9993032 |  |
| C | 40.9354744 | 8.8503931 | 15.2582373 | C | 41.0004672 | 8.8506382 | 15.3165258 |  |
| C | 41.8915988 | 9.3021876 | 16.3652413 | C | 41.0866560 | 9.5642641 | 16.6748838 |  |
| S | 43.6686400 | 9.0504438 | 15.9079236 | S | 44.2394691 | 8.7690769 | 15.9405173 |  |
| C | 43.7130415 | 7.4109253 | 15.1286034 | C | 44.5103601 | 7.1525591 | 15.1322689 |  |
| C | 44.4484939 | 8.7462677 | 17.5505453 | C | 44.9436909 | 8.5338442 | 17.6200803 |  |
| C | 45.9552623 | 8.9442899 | 17.5232411 | C | 46.3943194 | 8.9865545 | 17.7415840 |  |
| O | 46.5198314 | 7.9419816 | 16.6921591 | O | 47.2078884 | 8.0106060 | 17.0880515 |  |
| C | 46.5186858 | 10.3163555 | 17.0262879 | C | 46.7320997 | 10.3718875 | 17.1214554 |  |
| O | 47.1550030 | 11.0502606 | 18.0328603 | O | 47.2939886 | 11.2830625 | 18.0275524 |  |
| C | 47.4409254 | 9.8824635 | 15.8400332 | C | 47.6384598 | 9.9988738 | 15.9068576 |  |
| O | 48.5047975 | 10.7102100 | 15.5321928 | O | 48.5947846 | 10.9397720 | 15.5330567 |  |
| C | 47.7675470 | 8.4268237 | 16.2236043 | C | 48.2044047 | 8.6218894 | 16.3049406 |  |
| O | 39.0207303 | 8.6028670 | 13.2064906 | O | 38.7559861 | 8.4645991 | 13.3017273 |  |
| N | 50.9804822 | 4.6647925 | 14.9636951 | N | 51.0194640 | 4.6144624 | 14.9176934 |  |
| C | 50.9533761 | 5.4886277 | 16.0206491 | C | 51.1272423 | 5.4629731 | 15.9489719 |  |
| N | 50.1173619 | 6.4954479 | 16.2573621 | N | 50.3952085 | 6.5431715 | 16.2027818 |  |
| C | 49.2195207 | 6.6270990 | 15.2708404 | C | 49.4479464 | 6.7204625 | 15.2699494 |  |
| C | 49.1239332 | 5.8426028 | 14.1126066 | C | 49.2157573 | 5.9135721 | 14.1442585 |  |
| C | 50.0810000 | 4.7970000 | 13.9630000 | C | 50.0810000 | 4.7970000 | 13.9630000 | f |
| N | 50.1126787 | 3.9659831 | 12.9142510 | N | 50.0070710 | 3.9423517 | 12.9310192 |  |
| N | 48.0680605 | 6.2650355 | 13.3249730 | N | 48.1470059 | 6.4009878 | 13.4145946 |  |
| C | 47.5385727 | 7.2642925 | 13.9881817 | C | 47.7402578 | 7.4526845 | 14.0849058 |  |
| N | 48.2008502 | 7.5531701 | 15.1679466 | N | 48.5021061 | 7.7227911 | 15.2065989 |  |
| H | 39.2672557 | 10.6157460 | 17.0765814 | H | 39.3519429 | 10.9394653 | 16.7517510 |  |
| H | 39.1833340 | 8.9624870 | 17.2663313 | H | 39.0244121 | 9.3050172 | 17.0825532 |  |
| H | 49.4771146 | 4.1733471 | 12.1525246 | H | 49.3684884 | 4.1908342 | 12.1842426 |  |
| H | 50.9862022 | 3.4281517 | 12.7007277 | H | 50.8597148 | 3.3858973 | 12.6939207 |  |
| H | 49.2180357 | 10.6597620 | 16.2394184 | H | 49.2816257 | 10.9879608 | 16.2548265 |  |
| H | 47.9887976 | 10.5723187 | 18.3530854 | H | 48.1228201 | 10.8837928 | 18.4500883 |  |
| H | 51.3113717 | 2.3516715 | 17.5656073 | H | 51.2752032 | 2.3665782 | 17.5166180 |  |
| H | 51.2326950 | 0.9695291 | 16.4320172 | H | 51.2665797 | 0.9279822 | 16.4532501 |  |
| H | 52.6726198 | 1.1816630 | 17.4759300 | H | 52.6716711 | 1.2341868 | 17.5211509 |  |
| H | 49.1715878 | 8.6473726 | 11.1089180 | H | 49.1776182 | 8.6495336 | 11.1092688 |  |
| H | 49.1203091 | 10.3778095 | 10.6793851 | H | 49.1080117 | 10.3789366 | 10.6754213 |  |
| H | 49.3474584 | 9.8880241 | 12.3757610 | H | 49.3542005 | 9.8953330 | 12.3701636 |  |
| H | 47.3473926 | 11.3267333 | 13.0176496 | H | 47.3449969 | 11.3088742 | 13.0392842 |  |
| H | 46.9292559 | 7.8889582 | 10.4588032 | H | 46.9403703 | 7.8653842 | 10.4854579 |  |
| H | 44.9087528 | 11.2718970 | 13.4819557 | H | 44.9139078 | 11.2180215 | 13.5380211 |  |
| H | 44.4945785 | 7.8162783 | 10.9526712 | H | 44.5069628 | 7.7765505 | 10.9929855 |  |
| H | 42.7672394 | 8.7441003 | 12.1631593 | H | 42.7836871 | 8.6953101 | 12.2007910 |  |
| H | 42.6798455 | 15.9061136 | 14.0084296 | H | 42.6812008 | 15.9154300 | 14.0371999 |  |
| H | 41.1062605 | 15.0655037 | 13.9284731 | H | 41.1085347 | 15.0800415 | 13.9233247 |  |
| H | 43.8718032 | 13.7579622 | 14.2428125 | H | 43.8588080 | 13.7403809 | 14.2414079 |  |
| H | 41.8373007 | 13.6226212 | 11.9576986 | H | 41.9062882 | 13.7090910 | 11.8822350 |  |
| H | 43.3077029 | 12.6589739 | 12.0717921 | H | 43.3903684 | 12.7640490 | 12.0108960 |  |
| H | 42.6360920 | 11.5503684 | 14.0658451 | H | 42.5847809 | 11.5602693 | 13.8992648 |  |
| H | 42.2272839 | 12.4362056 | 15.5603137 | H | 42.1822031 | 12.3672460 | 15.4349693 |  |
| H | 41.0947680 | 12.4266980 | 14.2010806 | H | 41.0635707 | 12.4746313 | 14.0608638 |  |
| H | 43.7395159 | 14.5673466 | 10.5694563 | H | 43.8449390 | 14.7339753 | 10.6041403 |  |
| H | 43.2211364 | 15.7405123 | 11.7962890 | H | 43.2554519 | 15.8515164 | 11.8498866 |  |
| H | 44.7053182 | 14.7875240 | 12.0467146 | H | 44.7472685 | 14.9170175 | 12.1257745 |  |
| H | 43.8157006 | 15.8709257 | 16.0772357 | H | 43.7601185 | 15.9218563 | 16.0781857 |  |
| H | 42.3628069 | 14.7938668 | 18.4419206 | H | 42.3636927 | 14.8016373 | 18.4573295 |  |
| H | 43.1388159 | 16.3962491 | 18.5258253 | H | 43.1635587 | 16.3959500 | 18.5277833 |  |
| H | 44.6581497 | 14.6950545 | 19.4832785 | H | 44.6723770 | 14.6554659 | 19.4476499 |  |
| H | 45.3663962 | 15.3716616 | 18.0146378 | H | 45.3628331 | 15.3440195 | 17.9768160 |  |
| H | 45.7100544 | 12.9313759 | 17.9534634 | H | 45.7020118 | 12.9256167 | 17.8600869 |  |
| H | 44.5382114 | 13.3664346 | 16.6965496 | H | 44.4853812 | 13.3594301 | 16.6482977 |  |
| H | 43.7908332 | 10.7692061 | 20.3904439 | H | 43.9306307 | 10.6666104 | 20.2971452 |  |
| H | 45.3780755 | 11.2435877 | 19.6767012 | H | 45.4725166 | 11.2760956 | 19.5916339 |  |
| H | 44.4298973 | 12.4150006 | 20.6723535 | H | 44.4585826 | 12.3427370 | 20.6389254 |  |
| H | 52.5109183 | 10.3151148 | 18.8599578 | H | 52.6024580 | 10.1608315 | 18.8058234 |  |
| H | 51.5216895 | 9.5881791 | 20.1932500 | H | 51.6047938 | 9.5782431 | 20.2024649 |  |
| H | 51.9716958 | 8.6205421 | 18.7576157 | H | 51.7533337 | 8.5986200 | 18.7134091 |  |
| H | 39.6054315 | 6.8163909 | 21.2950948 | H | 39.7149023 | 6.7941573 | 21.1911807 |  |
| H | 38.1259963 | 7.6557032 | 20.8650917 | H | 38.1764099 | 7.6289942 | 21.0152276 |  |
| H | 40.9597062 | 8.6746415 | 20.2024669 | H | 40.9314516 | 8.7002427 | 20.1203265 |  |
| H | 40.1190236 | 9.0826665 | 21.6949909 | H | 40.1332797 | 9.1271708 | 21.6258317 |  |
| H | 37.2775794 | 9.3818786 | 19.9762728 | H | 37.2596472 | 9.2453263 | 19.8227088 |  |
| H | 41.2567555 | 11.0356544 | 19.7369122 | H | 41.1449322 | 11.1350779 | 19.7436579 |  |
| H | 36.3685735 | 11.4330287 | 18.9673635 | H | 36.2716722 | 11.2432183 | 18.7760745 |  |
| H | 40.3487511 | 13.0773439 | 18.6813639 | H | 40.1581938 | 13.1229171 | 18.6627511 |  |
| H | 37.8814301 | 13.3096791 | 18.3235652 | H | 37.6956337 | 13.2137168 | 18.2121801 |  |
| H | 38.0081071 | 5.3389051 | 19.8650958 | H | 38.2882469 | 5.1785468 | 19.9178409 |  |
| H | 37.8951819 | 3.9837480 | 17.9890003 | H | 37.9508080 | 3.9673767 | 17.9988071 |  |
| H | 37.5475222 | 6.8134747 | 16.8329008 | H | 37.4112543 | 6.7754275 | 16.8860485 |  |
| H | 36.2786513 | 5.8286689 | 17.6000824 | H | 36.2217370 | 5.6729340 | 17.6170339 |  |
| H | 36.6497884 | 5.5317040 | 13.6833846 | H | 36.6701340 | 5.5892293 | 13.6841263 |  |
| H | 37.0254634 | 6.9053010 | 14.6655301 | H | 37.0026060 | 6.9105056 | 14.7395165 |  |
| H | 40.4707820 | 3.9231276 | 18.4137299 | H | 40.5127815 | 4.1118545 | 18.4789759 |  |
| H | 41.9182986 | 4.6129391 | 15.9006268 | H | 41.8954615 | 4.5156388 | 15.8671787 |  |
| H | 42.3459293 | 3.1761848 | 16.8394991 | H | 42.2401254 | 3.1388274 | 16.9149985 |  |
| H | 42.8001334 | 4.6548197 | 18.8626876 | H | 42.9492580 | 4.7998389 | 18.7530646 |  |
| H | 42.3759798 | 6.0640445 | 17.9013245 | H | 42.7700134 | 6.0980011 | 17.5666436 |  |
| H | 32.7961383 | 5.5719165 | 15.5018523 | H | 32.8364368 | 5.5894391 | 15.5057957 |  |
| H | 33.1375873 | 5.1586795 | 17.1848313 | H | 33.1526803 | 5.1795101 | 17.1944906 |  |
| H | 31.5171115 | 5.7441286 | 16.7391903 | H | 31.5197452 | 5.6939132 | 16.7105105 |  |
| H | 31.2841811 | 7.8823633 | 17.9120728 | H | 31.1490473 | 7.8055506 | 17.8759995 |  |
| H | 34.9093607 | 7.0587975 | 15.7327978 | H | 34.8817872 | 7.1659221 | 15.8186795 |  |
| H | 32.0877210 | 10.1886779 | 18.3637103 | H | 31.8171867 | 10.1480140 | 18.3564864 |  |
| H | 35.7613137 | 9.3169658 | 16.2427744 | H | 35.5791655 | 9.4762428 | 16.3318486 |  |
| H | 34.3297749 | 10.9275254 | 17.5308983 | H | 34.0363767 | 11.0068240 | 17.5840309 |  |
| H | 30.6688355 | 9.6909107 | 14.4789436 | H | 30.6770026 | 9.6989114 | 14.4956215 |  |
| H | 32.3942503 | 9.6743024 | 14.9191698 | H | 32.4081943 | 9.6751671 | 14.9058442 |  |
| H | 31.8962178 | 10.1713759 | 13.2765228 | H | 31.8838384 | 10.1667325 | 13.2698402 |  |
| H | 31.0453898 | 7.8405226 | 12.8361054 | H | 31.0178273 | 7.8438073 | 12.8490653 |  |
| H | 31.4831273 | 7.3691840 | 14.4957891 | H | 31.4615202 | 7.3760482 | 14.5079237 |  |
| H | 33.8880641 | 8.3598729 | 13.3215459 | H | 33.8694643 | 8.3171604 | 13.3253879 |  |
| H | 32.7481244 | 4.6862261 | 12.0349694 | H | 32.6263546 | 4.6593702 | 12.0684148 |  |
| H | 31.6321640 | 5.6152370 | 12.9671004 | H | 31.5608053 | 5.6135069 | 13.0341902 |  |
| H | 34.8898637 | 5.4518615 | 11.6926579 | H | 34.7795840 | 5.3571954 | 11.6958424 |  |
| H | 35.4261386 | 7.0341277 | 12.1165542 | H | 35.3627243 | 6.9378495 | 12.0388031 |  |
| H | 33.5817137 | 14.5698840 | 13.5546998 | H | 33.5739498 | 14.5583496 | 13.5610153 |  |
| H | 33.1266642 | 14.4751938 | 15.2658072 | H | 33.1292628 | 14.4919120 | 15.2771379 |  |
| H | 33.8582430 | 15.9684770 | 14.6246097 | H | 33.8709307 | 15.9690914 | 14.6096429 |  |
| H | 36.1864313 | 16.2797154 | 15.1437117 | H | 36.2229886 | 16.2542959 | 15.1008718 |  |
| H | 34.7217108 | 12.2420212 | 14.7403526 | H | 34.6842427 | 12.2382407 | 14.7728203 |  |
| H | 38.4578826 | 15.3981604 | 15.6420502 | H | 38.4811353 | 15.3433504 | 15.6000421 |  |
| H | 36.9898161 | 11.3696892 | 15.2638189 | H | 36.9345001 | 11.3394627 | 15.2660500 |  |
| H | 39.7568806 | 13.5060308 | 15.9289351 | H | 39.7663472 | 13.4264378 | 15.9092613 |  |
| H | 34.6818326 | 13.7484772 | 8.9638364 | H | 34.6883832 | 13.7245592 | 8.9572288 |  |
| H | 35.4219980 | 14.6629882 | 10.2964992 | H | 35.4087786 | 14.6734589 | 10.2777787 |  |
| H | 36.4560618 | 13.8495767 | 9.1076136 | H | 36.4612699 | 13.8434796 | 9.1158534 |  |
| H | 37.6036987 | 12.6599421 | 11.1049914 | H | 37.5999685 | 12.6976608 | 11.1518181 |  |
| H | 33.4439273 | 12.0225253 | 10.1754807 | H | 33.4541056 | 12.0077449 | 10.1975891 |  |
| H | 37.5892902 | 10.6690396 | 12.5694699 | H | 37.5825833 | 10.7476764 | 12.6623219 |  |
| H | 33.4264894 | 10.0393159 | 11.6620899 | H | 33.4351070 | 10.0656494 | 11.7313983 |  |
| H | 36.2383456 | 9.0427203 | 13.6759632 | H | 36.1709365 | 9.1691785 | 13.8453523 |  |
| H | 38.9243057 | 6.6889556 | 10.5657964 | H | 38.9218690 | 6.7081477 | 10.6786520 |  |
| H | 37.2902549 | 6.4006581 | 9.9018858 | H | 37.3800575 | 6.4089809 | 9.8233562 |  |
| H | 37.8963538 | 8.0714268 | 10.0768839 | H | 37.9267784 | 8.0859988 | 10.1128672 |  |
| H | 37.8771028 | 7.6892043 | 12.3949622 | H | 37.6693813 | 7.6040765 | 12.4097254 |  |
| H | 42.9771702 | 4.8111647 | 11.2067835 | H | 42.9367408 | 4.7950660 | 11.1884182 |  |
| H | 43.2486113 | 4.8375583 | 12.9673160 | H | 43.2793391 | 4.8601438 | 12.9369813 |  |
| H | 41.7871409 | 4.0421378 | 12.3240800 | H | 41.8009715 | 4.0400585 | 12.3704691 |  |
| H | 40.1911385 | 7.2067210 | 13.1107988 | H | 40.1988943 | 7.1934755 | 13.1525660 |  |
| H | 40.3734083 | 5.5587096 | 13.6561419 | H | 40.4344263 | 5.5777701 | 13.7470115 |  |
| H | 55.2761364 | 3.9526495 | 14.7962281 | H | 55.2900108 | 3.9411337 | 14.7756444 |  |
| H | 53.6003237 | 4.1882855 | 14.1820505 | H | 53.6044456 | 4.1953232 | 14.1907463 |  |
| H | 55.3528095 | 1.7749020 | 13.4310267 | H | 55.3117232 | 1.7576278 | 13.4152468 |  |
| H | 55.1860401 | 3.2515181 | 12.4392638 | H | 55.1399939 | 3.2310207 | 12.4227335 |  |
| H | 51.9851127 | 3.2039194 | 15.3552291 | H | 51.9806526 | 3.1441871 | 15.3011423 |  |
| H | 39.7078959 | 10.5965238 | 14.9229116 | H | 39.8728926 | 10.5740731 | 14.5449243 |  |
| H | 41.3696399 | 9.0344211 | 14.2652437 | H | 41.8101530 | 9.0364850 | 14.6050678 |  |
| H | 40.7769034 | 7.7664606 | 15.3583178 | H | 40.8272476 | 7.7719331 | 15.4291012 |  |
| H | 41.7072242 | 8.7594793 | 17.3039492 | H | 41.2573907 | 8.9543112 | 17.5654652 |  |
| H | 41.8380107 | 10.3814652 | 16.5638210 | H | 41.7178539 | 10.4592004 | 16.6850115 |  |
| H | 44.7147368 | 6.9981486 | 15.3048656 | H | 45.4323303 | 6.6925852 | 15.5073964 |  |
| H | 42.9701843 | 6.7544674 | 15.5905936 | H | 43.6759625 | 6.4625563 | 15.3106585 |  |
| H | 43.5058192 | 7.5727242 | 14.0655566 | H | 44.5926019 | 7.3473690 | 14.0533073 |  |
| H | 44.2381290 | 7.7086801 | 17.8630406 | H | 44.8831126 | 7.4663772 | 17.8917577 |  |
| H | 43.9720225 | 9.4801794 | 18.2096236 | H | 44.3098188 | 9.1311641 | 18.2919389 |  |
| H | 46.2978436 | 8.8077073 | 18.5677017 | H | 46.6676799 | 9.0209056 | 18.8138374 |  |
| H | 45.7023340 | 10.9500602 | 16.6432404 | H | 45.8130562 | 10.8408741 | 16.7395262 |  |
| H | 46.7927730 | 9.8193907 | 14.9516236 | H | 46.9573852 | 9.8465222 | 15.0579525 |  |
| H | 48.5377775 | 8.3871535 | 17.0142271 | H | 49.1477143 | 8.7386028 | 16.8609140 |  |
| H | 51.7209574 | 5.2927445 | 16.7804065 | H | 51.9213459 | 5.2206548 | 16.6670384 |  |
| H | 46.6660211 | 7.8338385 | 13.6754997 | H | 46.8936214 | 8.0766573 | 13.8154086 |  |

| ***AzeJ_Y175F*** Reactant | ***AzeJ_Y75F*** Product | Fixed (f) |
| --- | --- | --- |

| C | 48.8200000 | 9.6360000 | 11.4410000 | C | 48.8200000 | 9.6360000 | 11.4410000 | f |
| --- | --- | --- | --- | --- | --- | --- | --- | --- |
| C | 47.3349700 | 9.6139044 | 11.7383833 | C | 47.3259955 | 9.6305665 | 11.6894841 |  |
| C | 46.7568457 | 10.5278084 | 12.6375206 | C | 46.7266726 | 10.5631165 | 12.5566626 |  |
| C | 46.4814332 | 8.6672626 | 11.1483442 | C | 46.4877029 | 8.6714215 | 11.0993472 |  |
| C | 45.3888769 | 10.5039030 | 12.9298125 | C | 45.3543280 | 10.5405377 | 12.8228225 |  |
| C | 45.1139142 | 8.6184088 | 11.4398280 | C | 45.1139736 | 8.6279457 | 11.3615821 |  |
| C | 44.5527795 | 9.5460627 | 12.3343136 | C | 44.5335937 | 9.5686036 | 12.2287757 |  |
| O | 43.2268948 | 9.5468824 | 12.6553632 | O | 43.2056763 | 9.5609701 | 12.5328047 |  |
| C | 42.1220000 | 15.0210000 | 14.3510000 | C | 42.1220000 | 15.0210000 | 14.3510000 | f |
| C | 41.9505542 | 15.0864338 | 15.8846656 | C | 41.9728695 | 14.9582553 | 15.8861999 |  |
| O | 40.9110672 | 14.7194209 | 16.4306032 | O | 41.0095069 | 14.4013477 | 16.4160159 |  |
| C | 42.6365256 | 13.6184173 | 13.9232599 | C | 42.6702823 | 13.6848117 | 13.7830566 |  |
| C | 42.9223144 | 13.5451902 | 12.4111434 | C | 43.0904620 | 13.8216734 | 12.3069533 |  |
| C | 41.6726116 | 12.4967501 | 14.3367338 | C | 41.6679224 | 12.5359756 | 13.9532657 |  |
| C | 44.0909863 | 14.4050218 | 11.9273150 | C | 44.3155658 | 14.7050402 | 12.0625278 |  |
| N | 43.0436348 | 15.4906928 | 16.6001345 | N | 42.9959451 | 15.5058117 | 16.6074511 |  |
| C | 43.1990000 | 15.3970000 | 18.0630000 | C | 43.1990000 | 15.3970000 | 18.0630000 | f |
| C | 44.5382659 | 14.7427693 | 18.4208435 | C | 44.5347913 | 14.7188433 | 18.3826182 |  |
| C | 44.7269708 | 13.3470711 | 17.8223936 | C | 44.6525351 | 13.2951681 | 17.8365396 |  |
| S | 43.4951807 | 12.1241668 | 18.4150095 | S | 43.5054266 | 12.0922138 | 18.6163209 |  |
| C | 44.2908602 | 11.6322038 | 19.9866911 | C | 44.4968085 | 11.6443115 | 20.0858352 |  |
| C | 51.6880000 | 9.6280000 | 19.1070000 | C | 51.6880000 | 9.6280000 | 19.1070000 | f |
| C | 50.3513126 | 9.8679647 | 18.3786585 | C | 50.3946957 | 10.0875705 | 18.4302265 |  |
| O | 49.2817234 | 9.5779321 | 18.9940468 | O | 49.3115365 | 9.8439316 | 19.0407753 |  |
| O | 50.4112660 | 10.3166059 | 17.2064178 | O | 50.4931250 | 10.6327931 | 17.3031611 |  |
| C | 39.1800000 | 7.5290000 | 20.5670000 | C | 39.1800000 | 7.5290000 | 20.5670000 | f |
| C | 39.1654281 | 6.8716284 | 19.1737295 | C | 39.0156283 | 6.9282035 | 19.1579504 |  |
| O | 39.7232902 | 7.3864601 | 18.2075136 | O | 39.3231392 | 7.5571463 | 18.1422336 |  |
| C | 39.9813853 | 8.8281549 | 20.6298591 | C | 39.9031555 | 8.8769586 | 20.5689658 |  |
| C | 39.3848913 | 10.0540756 | 19.9595036 | C | 39.2195080 | 10.0433394 | 19.8746769 |  |
| C | 38.0035420 | 10.2494297 | 19.8086851 | C | 37.8430006 | 10.0725687 | 19.5956937 |  |
| C | 40.2448514 | 11.0708055 | 19.5092828 | C | 39.9941057 | 11.1553757 | 19.4965660 |  |
| C | 37.5022937 | 11.4239269 | 19.2351557 | C | 37.2617719 | 11.1751308 | 18.9568610 |  |
| C | 39.7536523 | 12.2429646 | 18.9257074 | C | 39.4262214 | 12.2544665 | 18.8430797 |  |
| C | 38.3712014 | 12.4259094 | 18.7946570 | C | 38.0490209 | 12.2691034 | 18.5745638 |  |
| N | 38.5272581 | 5.6630784 | 19.0672158 | N | 38.5281362 | 5.6591998 | 19.0687627 |  |
| C | 38.2620000 | 4.9980000 | 17.7770000 | C | 38.2620000 | 4.9980000 | 17.7770000 | f |
| C | 39.5693275 | 4.9001293 | 16.9221620 | C | 39.5658694 | 4.9322269 | 16.9098312 |  |
| O | 39.5676781 | 5.1986247 | 15.7301403 | O | 39.5317526 | 5.1800006 | 15.7071875 |  |
| C | 37.1833639 | 5.7342697 | 16.9718230 | C | 37.1377210 | 5.7025652 | 17.0044479 |  |
| C | 36.8431578 | 5.0013186 | 15.6760625 | C | 36.8078690 | 5.0129408 | 15.6831180 |  |
| N | 36.6851657 | 5.7908628 | 14.5908541 | N | 36.6195060 | 5.8531655 | 14.6352484 |  |
| O | 36.6790882 | 3.7833458 | 15.6643742 | O | 36.6643835 | 3.7971557 | 15.6151218 |  |
| N | 40.6600222 | 4.4248641 | 17.5615537 | N | 40.6803659 | 4.5352846 | 17.5630136 |  |
| C | 41.9510000 | 4.2010000 | 16.9160000 | C | 41.9510000 | 4.2010000 | 16.9160000 | f |
| C | 43.1245592 | 4.6427220 | 17.7971297 | C | 43.1718061 | 4.7246863 | 17.6754542 |  |
| C | 44.4852198 | 4.0320567 | 17.4061065 | C | 44.4841759 | 3.9936412 | 17.3204796 |  |
| C | 44.9245030 | 4.3575126 | 15.9711649 | C | 44.7569517 | 3.9132736 | 15.8108196 |  |
| C | 45.5768045 | 4.4615937 | 18.3960602 | C | 45.6744783 | 4.6290385 | 18.0520777 |  |
| C | 32.5930000 | 5.8730000 | 16.5430000 | C | 32.5930000 | 5.8730000 | 16.5430000 | f |
| C | 33.0527494 | 7.2896371 | 16.8131344 | C | 32.9555146 | 7.3133004 | 16.8301053 |  |
| C | 32.2608231 | 8.1963996 | 17.5365282 | C | 32.0894615 | 8.1612029 | 17.5399959 |  |
| C | 34.3102169 | 7.7249193 | 16.3596360 | C | 34.1875880 | 7.8354835 | 16.3986339 |  |
| C | 32.7185991 | 9.4936125 | 17.8053176 | C | 32.4456225 | 9.4877510 | 17.8153747 |  |
| C | 34.7856319 | 9.0096603 | 16.6419281 | C | 34.5538536 | 9.1561314 | 16.6774402 |  |
| C | 33.9829210 | 9.9018283 | 17.3641763 | C | 33.6793761 | 9.9894584 | 17.3859272 |  |
| C | 31.6810000 | 9.4790000 | 14.1040000 | C | 31.6810000 | 9.4790000 | 14.1040000 | f |
| C | 31.7308085 | 8.0210845 | 13.6617619 | C | 31.7083604 | 8.0232532 | 13.6552547 |  |
| N | 33.0627532 | 7.6640703 | 13.1778326 | N | 33.0290515 | 7.6475728 | 13.1514975 |  |
| C | 33.3649554 | 6.5142505 | 12.5780118 | C | 33.3001850 | 6.4871223 | 12.5539429 |  |
| N | 32.4349650 | 5.5442421 | 12.4754199 | N | 32.3501658 | 5.5359722 | 12.4732169 |  |
| N | 34.5772554 | 6.3108792 | 12.0516603 | N | 34.4997324 | 6.2548896 | 12.0139533 |  |
| C | 33.8900000 | 14.8690000 | 14.5720000 | C | 33.8900000 | 14.8690000 | 14.5720000 | f |
| C | 35.2263155 | 14.2251987 | 14.8781137 | C | 35.2744365 | 14.3124786 | 14.8317170 |  |
| C | 36.3665460 | 15.0007650 | 15.1491459 | C | 36.3689722 | 15.1570021 | 15.0869748 |  |
| C | 35.3561667 | 12.8237122 | 14.9042023 | C | 35.5010168 | 12.9240328 | 14.8201269 |  |
| C | 37.5982844 | 14.3993277 | 15.4428875 | C | 37.6482598 | 14.6352639 | 15.3191024 |  |
| C | 36.5792673 | 12.2157199 | 15.2027921 | C | 36.7755496 | 12.3977249 | 15.0520406 |  |
| C | 37.7025263 | 13.0060219 | 15.4734819 | C | 37.8562075 | 13.2532565 | 15.2985323 |  |
| C | 35.5190000 | 13.7510000 | 9.6800000 | C | 35.5190000 | 13.7510000 | 9.6800000 | f |
| C | 35.5075868 | 12.4995537 | 10.5234662 | C | 35.5076922 | 12.5077484 | 10.5409496 |  |
| C | 36.6704204 | 12.0271173 | 11.1543740 | C | 36.6765137 | 12.0105108 | 11.1414829 |  |
| C | 34.3214397 | 11.7795249 | 10.7317667 | C | 34.3118537 | 11.8169452 | 10.7938018 |  |
| C | 36.6558740 | 10.9060242 | 11.9880669 | C | 36.6576484 | 10.8968179 | 11.9882320 |  |
| C | 34.2879310 | 10.6602026 | 11.5637372 | C | 34.2750024 | 10.7091284 | 11.6414078 |  |
| C | 35.4490331 | 10.2308922 | 12.2229760 | C | 35.4428333 | 10.2583745 | 12.2720202 |  |
| O | 35.3462552 | 9.1685902 | 13.0652254 | O | 35.3332578 | 9.2005291 | 13.1314085 |  |
| C | 37.8850000 | 7.0690000 | 10.5480000 | C | 37.8850000 | 7.0690000 | 10.5480000 | f |
| O | 37.2684067 | 7.0321226 | 11.8201114 | O | 37.1529358 | 6.9787081 | 11.7600476 |  |
| C | 42.4750000 | 4.8880000 | 12.1800000 | C | 42.4750000 | 4.8880000 | 12.1800000 | f |
| C | 41.7789123 | 6.2249270 | 12.3279407 | C | 41.7852951 | 6.2309214 | 12.3143676 |  |
| N | 40.6760831 | 6.2848321 | 13.0845958 | N | 40.7253054 | 6.3156821 | 13.1348360 |  |
| O | 42.2369491 | 7.2253221 | 11.7473508 | O | 42.1948677 | 7.2058475 | 11.6644502 |  |
| C | 54.3690000 | 3.4430000 | 14.4320000 | C | 54.3690000 | 3.4430000 | 14.4320000 | f |
| C | 53.9021116 | 2.5107371 | 15.5837738 | C | 53.9074745 | 2.5246420 | 15.5980730 |  |
| O | 54.7136514 | 1.7839198 | 16.1626998 | O | 54.7264267 | 1.8278546 | 16.2039057 |  |
| C | 54.6545710 | 2.6174208 | 13.1662028 | C | 54.6131675 | 2.6042074 | 13.1652777 |  |
| C | 53.3374791 | 2.1256292 | 12.5173434 | C | 53.2770676 | 2.1006256 | 12.5628850 |  |
| O | 52.5544009 | 3.0639483 | 12.1637481 | O | 52.4794524 | 3.0303398 | 12.2229928 |  |
| O | 53.1435666 | 0.9043332 | 12.4021328 | O | 53.0871778 | 0.8760636 | 12.4690642 |  |
| N | 52.5743565 | 2.5272027 | 15.8735802 | N | 52.5755772 | 2.5166710 | 15.8660365 |  |
| C | 51.9070000 | 1.7130000 | 16.8920000 | C | 51.9070000 | 1.7130000 | 16.8920000 | f |
| N | 38.7802021 | 9.7947075 | 16.6038761 | N | 39.3939019 | 9.8773049 | 16.6066916 |  |
| C | 39.4080079 | 9.6239557 | 15.3190536 | C | 39.4640625 | 9.7412396 | 15.1042187 |  |
| C | 38.4840243 | 8.8870593 | 14.3328478 | C | 38.3946721 | 8.9178412 | 14.4199665 |  |
| O | 37.2902358 | 8.6485029 | 14.6713501 | O | 37.2587655 | 8.8246132 | 14.9599404 |  |
| C | 40.8015013 | 8.9323766 | 15.2838427 | C | 40.8951750 | 9.1969164 | 15.2188042 |  |
| C | 41.7175098 | 9.4235194 | 16.4023291 | C | 40.9175601 | 9.7488358 | 16.6519325 |  |
| S | 43.5020119 | 9.1526451 | 15.9946961 | S | 43.9568950 | 9.1120797 | 15.9478367 |  |
| C | 43.5353697 | 7.5447324 | 15.1624348 | C | 44.0326418 | 7.4924311 | 15.1234879 |  |
| C | 44.2136066 | 8.7234736 | 17.6406611 | C | 44.6085961 | 8.7357563 | 17.6189947 |  |
| C | 45.7322047 | 8.7890539 | 17.6746026 | C | 46.1091626 | 8.9507897 | 17.7350179 |  |
| O | 46.2646357 | 7.7339170 | 16.8719715 | O | 46.7617078 | 7.9053089 | 17.0062994 |  |
| C | 46.3952724 | 10.1249365 | 17.1971846 | C | 46.6172081 | 10.3096467 | 17.1657764 |  |
| O | 47.0940444 | 10.7827902 | 18.2074445 | O | 47.2648295 | 11.1129349 | 18.1088772 |  |
| C | 47.2546493 | 9.6402310 | 15.9911746 | C | 47.4852599 | 9.8601449 | 15.9509894 |  |
| O | 48.3318877 | 10.4161737 | 15.6280927 | O | 48.5077971 | 10.7101014 | 15.5599823 |  |
| C | 47.5410106 | 8.1772328 | 16.3828172 | C | 47.9101964 | 8.4365916 | 16.3578975 |  |
| O | 38.9671534 | 8.5711340 | 13.2128503 | O | 38.7338360 | 8.4400822 | 13.3154517 |  |
| N | 50.9458034 | 4.6371574 | 14.9902961 | N | 50.9913133 | 4.6266599 | 14.9474175 |  |
| C | 50.8093940 | 5.3467654 | 16.1187663 | C | 50.9921641 | 5.4188143 | 16.0282518 |  |
| N | 49.8870047 | 6.2598348 | 16.4126704 | N | 50.1734719 | 6.4287345 | 16.3085795 |  |
| C | 49.0304114 | 6.4295549 | 15.3967387 | C | 49.2632556 | 6.6009425 | 15.3389686 |  |
| C | 49.0409099 | 5.7585566 | 14.1673604 | C | 49.1361561 | 5.8480512 | 14.1627377 |  |
| C | 50.0810000 | 4.7970000 | 13.9630000 | C | 50.0810000 | 4.7970000 | 13.9630000 | f |
| N | 50.2034672 | 4.0799383 | 12.8462295 | N | 50.0869431 | 3.9978825 | 12.8904289 |  |
| N | 48.0085309 | 6.2062363 | 13.3620018 | N | 48.0803132 | 6.3158255 | 13.4014053 |  |
| C | 47.3962576 | 7.1201833 | 14.0747277 | C | 47.5842990 | 7.3138032 | 14.0942820 |  |
| N | 47.9637896 | 7.3074369 | 15.3258174 | N | 48.2633091 | 7.5504096 | 15.2775427 |  |
| H | 39.2351407 | 10.5150078 | 17.1565798 | H | 49.4484601 | 4.2456286 | 12.1428012 |  |
| H | 38.8006669 | 8.9354219 | 17.1501736 | H | 50.9647977 | 3.4682153 | 12.6439576 |  |
| H | 49.5662327 | 4.3031405 | 12.0901176 | H | 49.2058706 | 10.7453818 | 16.2787012 |  |
| H | 51.0942351 | 3.5590223 | 12.6181703 | H | 48.0792957 | 10.6266383 | 18.4681133 |  |
| H | 49.0747261 | 10.3804632 | 16.3128291 | H | 51.2742269 | 2.3531863 | 17.5295288 |  |
| H | 47.9409576 | 10.2855128 | 18.4659861 | H | 51.2696784 | 0.9343336 | 16.4370721 |  |
| H | 51.3318906 | 2.3513941 | 17.5840971 | H | 52.6710103 | 1.2224750 | 17.5128282 |  |
| H | 51.2147643 | 0.9870525 | 16.4306254 | H | 49.1730838 | 8.6439223 | 11.1207265 |  |
| H | 52.6704859 | 1.1603403 | 17.4586227 | H | 49.1024110 | 10.3654811 | 10.6609847 |  |
| H | 49.1650201 | 8.6609044 | 11.0651227 | H | 49.3605224 | 9.9093516 | 12.3621567 |  |
| H | 49.0707239 | 10.4001749 | 10.6841403 | H | 47.3564565 | 11.2842794 | 13.0859357 |  |
| H | 49.3916326 | 9.8715354 | 12.3533827 | H | 46.9224684 | 7.9094498 | 10.4456841 |  |
| H | 47.3964195 | 11.2408382 | 13.1649097 | H | 44.9091836 | 11.2437116 | 13.5295086 |  |
| H | 46.8991651 | 7.9203783 | 10.4671087 | H | 44.4820703 | 7.8542832 | 10.9191808 |  |
| H | 44.9574023 | 11.2152120 | 13.6382202 | H | 42.7654924 | 8.7634112 | 12.1515714 |  |
| H | 44.4722114 | 7.8562814 | 10.9928909 | H | 42.7813174 | 15.8560953 | 14.0696339 |  |
| H | 42.7667723 | 8.7850674 | 12.2228346 | H | 41.1229890 | 15.2170935 | 13.9300972 |  |
| H | 42.8151045 | 15.8034536 | 14.0052613 | H | 43.5815845 | 13.4266942 | 14.3583200 |  |
| H | 41.1335082 | 15.2062342 | 13.9034252 | H | 42.2277047 | 14.1950083 | 11.7234410 |  |
| H | 43.5979973 | 13.4480791 | 14.4481217 | H | 43.3045651 | 12.8090887 | 11.9237824 |  |
| H | 41.9996457 | 13.8148163 | 11.8637058 | H | 42.0884708 | 11.5991534 | 13.5579077 |  |
| H | 43.1273510 | 12.4902922 | 12.1589262 | H | 41.4020663 | 12.3964041 | 15.0107711 |  |
| H | 42.0866724 | 11.5163676 | 14.0527157 | H | 40.7353701 | 12.7540497 | 13.4030828 |  |
| H | 41.4697309 | 12.5080825 | 15.4177149 | H | 44.5857396 | 14.7181983 | 10.9944038 |  |
| H | 40.7009159 | 12.6121817 | 13.8270249 | H | 44.1492938 | 15.7508978 | 12.3701558 |  |
| H | 44.2688809 | 14.2567047 | 10.8499659 | H | 45.1891778 | 14.3262449 | 12.6201437 |  |
| H | 43.9138008 | 15.4818269 | 12.0849505 | H | 43.7470041 | 15.9433378 | 16.0831565 |  |
| H | 45.0221693 | 14.1366987 | 12.4551878 | H | 42.3607347 | 14.8079192 | 18.4616448 |  |
| H | 43.8513057 | 15.8146236 | 16.0772683 | H | 43.1597540 | 16.4008192 | 18.5187386 |  |
| H | 42.3561859 | 14.8000315 | 18.4379083 | H | 44.6826696 | 14.7218304 | 19.4759802 |  |
| H | 43.1330037 | 16.3991685 | 18.5207248 | H | 45.3612187 | 15.3214455 | 17.9609914 |  |
| H | 44.6314359 | 14.7042348 | 19.5192434 | H | 45.6726836 | 12.8979961 | 17.9543137 |  |
| H | 45.3684676 | 15.3823393 | 18.0671251 | H | 44.4149377 | 13.2743194 | 16.7613388 |  |
| H | 45.7293566 | 12.9394553 | 18.0282555 | H | 43.9705289 | 10.8262924 | 20.6002850 |  |
| H | 44.6032867 | 13.3766360 | 16.7289794 | H | 45.4937363 | 11.3064909 | 19.7615155 |  |
| H | 43.6688035 | 10.8484388 | 20.4449923 | H | 44.5924562 | 12.4940254 | 20.7793330 |  |
| H | 45.3000980 | 11.2424072 | 19.7807220 | H | 52.5238745 | 10.2922678 | 18.8421662 |  |
| H | 44.3455307 | 12.4830017 | 20.6819441 | H | 51.5652253 | 9.5677984 | 20.1981371 |  |
| H | 52.4225471 | 10.3923087 | 18.8138808 | H | 51.9257598 | 8.6186543 | 18.7286209 |  |
| H | 51.5468383 | 9.6173153 | 20.1974124 | H | 39.7392601 | 6.8074065 | 21.1853253 |  |
| H | 52.0799084 | 8.6450640 | 18.7944608 | H | 38.1797081 | 7.6011149 | 21.0275017 |  |
| H | 39.6082384 | 6.7962808 | 21.2726948 | H | 40.9028399 | 8.7449113 | 20.1237632 |  |
| H | 38.1367620 | 7.6719760 | 20.8951859 | H | 40.0819982 | 9.1580991 | 21.6216237 |  |
| H | 40.9827772 | 8.6446175 | 20.2083990 | H | 37.2064564 | 9.2248249 | 19.8584441 |  |
| H | 40.1418210 | 9.0649990 | 21.6969493 | H | 41.0703794 | 11.1555073 | 19.6847191 |  |
| H | 37.2978588 | 9.4769165 | 20.1226074 | H | 36.1909788 | 11.1672441 | 18.7389060 |  |
| H | 41.3246242 | 10.9361007 | 19.6025489 | H | 40.0613938 | 13.0776167 | 18.5016149 |  |
| H | 36.4234154 | 11.5405954 | 19.1056362 | H | 37.6005070 | 13.1166143 | 18.0533110 |  |
| H | 40.4461159 | 12.9958012 | 18.5390877 | H | 38.2404562 | 5.1868286 | 19.9192700 |  |
| H | 37.9801814 | 13.3249592 | 18.3148727 | H | 37.9662832 | 3.9600970 | 17.9876023 |  |
| H | 38.0203975 | 5.3194704 | 19.8764313 | H | 37.4035494 | 6.7540342 | 16.8424693 |  |
| H | 37.9293528 | 3.9711234 | 17.9862808 | H | 36.2270426 | 5.6776034 | 17.6250634 |  |
| H | 37.5042170 | 6.7658586 | 16.7736675 | H | 36.5360559 | 5.4682688 | 13.6998797 |  |
| H | 36.2652243 | 5.7789243 | 17.5798571 | H | 36.8882457 | 6.8334799 | 14.7051733 |  |
| H | 36.5815093 | 5.3615066 | 13.6781887 | H | 40.5983321 | 4.3385086 | 18.5553391 |  |
| H | 36.9545567 | 6.7777433 | 14.6061266 | H | 41.9081971 | 4.6073823 | 15.8962422 |  |
| H | 40.5749928 | 4.2016589 | 18.5479885 | H | 42.0190022 | 3.1011827 | 16.8198252 |  |
| H | 41.9264965 | 4.7369624 | 15.9572953 | H | 42.9988716 | 4.6079351 | 18.7609215 |  |
| H | 42.0602136 | 3.1272086 | 16.6730847 | H | 43.2760332 | 5.8085135 | 17.4997238 |  |
| H | 42.9098897 | 4.3464226 | 18.8396762 | H | 44.3707795 | 2.9548459 | 17.6880113 |  |
| H | 43.1829943 | 5.7458877 | 17.8114012 | H | 45.6938315 | 3.3659692 | 15.6197210 |  |
| H | 44.3643052 | 2.9341622 | 17.4810765 | H | 43.9543778 | 3.3926077 | 15.2640751 |  |
| H | 45.8149660 | 3.7695546 | 15.6976085 | H | 44.8722456 | 4.9149984 | 15.3707708 |  |
| H | 44.1446520 | 4.1380609 | 15.2235009 | H | 46.5880266 | 4.0296707 | 17.9070238 |  |
| H | 45.2094063 | 5.4163643 | 15.8869730 | H | 45.8902255 | 5.6426590 | 17.6742934 |  |
| H | 46.5289876 | 3.9524625 | 18.1759187 | H | 45.4891828 | 4.7015188 | 19.1374573 |  |
| H | 45.7666802 | 5.5454739 | 18.3177729 | H | 32.8046888 | 5.6069091 | 15.4940793 |  |
| H | 45.2997454 | 4.2266318 | 19.4374452 | H | 33.1893152 | 5.1833824 | 17.1654165 |  |
| H | 32.8032030 | 5.5741860 | 15.5026438 | H | 31.5302970 | 5.6700151 | 16.7449569 |  |
| H | 33.1275233 | 5.1540729 | 17.1881841 | H | 31.1216170 | 7.7802859 | 17.8804636 |  |
| H | 31.5148967 | 5.7483586 | 16.7289765 | H | 34.8644119 | 7.1992372 | 15.8258359 |  |
| H | 31.2731696 | 7.8863314 | 17.8923219 | H | 31.7522954 | 10.1318541 | 18.3636035 |  |
| H | 34.9256295 | 7.0468333 | 15.7671330 | H | 35.5218494 | 9.5222668 | 16.3322509 |  |
| H | 32.0816260 | 10.1869109 | 18.3624397 | H | 33.9546003 | 11.0279379 | 17.5899085 |  |
| H | 35.7832305 | 9.2983054 | 16.3022526 | H | 30.6982064 | 9.7086828 | 14.5409504 |  |
| H | 34.3434405 | 10.9134861 | 17.5695902 | H | 32.4447480 | 9.6655375 | 14.8732571 |  |
| H | 30.6836853 | 9.7074371 | 14.5077727 | H | 31.8500990 | 10.1674271 | 13.2638749 |  |
| H | 32.4203172 | 9.6689012 | 14.8973848 | H | 30.9592407 | 7.8582050 | 12.8603339 |  |
| H | 31.8786104 | 10.1642189 | 13.2668891 | H | 31.4332992 | 7.3770272 | 14.5072275 |  |
| H | 30.9939537 | 7.8442063 | 12.8575165 | H | 33.8212399 | 8.2974978 | 13.2691246 |  |
| H | 31.4527909 | 7.3741461 | 14.5123934 | H | 32.5390140 | 4.6515551 | 12.0180245 |  |
| H | 33.8444360 | 8.3314813 | 13.2889375 | H | 31.4641872 | 5.6319563 | 12.9508573 |  |
| H | 32.6525559 | 4.6617127 | 12.0298840 | H | 34.7052399 | 5.3359764 | 11.6405366 |  |
| H | 31.5426212 | 5.6197335 | 12.9446876 | H | 35.2991858 | 6.9070661 | 12.0198034 |  |
| H | 34.8088354 | 5.3974655 | 11.6810853 | H | 33.5033451 | 14.5170587 | 13.6005861 |  |
| H | 35.3633974 | 6.9758480 | 12.0998407 | H | 33.1747210 | 14.5343314 | 15.3430389 |  |
| H | 33.4825133 | 14.4937072 | 13.6178067 | H | 33.8885623 | 15.9693941 | 14.5630980 |  |
| H | 33.1454937 | 14.6348995 | 15.3529005 | H | 36.2178532 | 16.2410302 | 15.1015506 |  |
| H | 33.9717093 | 15.9644193 | 14.5012956 | H | 34.6720963 | 12.2415220 | 14.6093198 |  |
| H | 36.2885449 | 16.0928349 | 15.1301344 | H | 38.4955669 | 15.2945174 | 15.5253989 |  |
| H | 34.4879925 | 12.1965356 | 14.6781759 | H | 36.9145818 | 11.3141910 | 15.0304634 |  |
| H | 38.4860224 | 14.9997983 | 15.6603916 | H | 34.6896940 | 13.7491456 | 8.9551729 |  |
| H | 36.6684112 | 11.1276161 | 15.2242015 | H | 35.4127166 | 14.6593165 | 10.2997315 |  |
| H | 34.6938679 | 13.7574760 | 8.9500549 | H | 36.4633447 | 13.8490671 | 9.1210824 |  |
| H | 35.4080375 | 14.6529956 | 10.3086140 | H | 37.6330475 | 12.5053229 | 10.9482665 |  |
| H | 36.4654191 | 13.8597662 | 9.1258337 | H | 33.3835525 | 12.1545630 | 10.3225208 |  |
| H | 37.6177306 | 12.5517240 | 10.9965779 | H | 37.5858782 | 10.5156871 | 12.4158063 |  |
| H | 33.4000264 | 12.1020556 | 10.2365563 | H | 33.3363410 | 10.1859815 | 11.8220847 |  |
| H | 37.5786264 | 10.5475492 | 12.4457660 | H | 36.0949361 | 9.0873176 | 13.7633873 |  |
| H | 33.3583015 | 10.1091558 | 11.7104330 | H | 38.9236437 | 6.7094042 | 10.6705831 |  |
| H | 36.1616961 | 8.9910431 | 13.6349402 | H | 37.3914023 | 6.4386269 | 9.7904688 |  |
| H | 38.9223760 | 6.6842843 | 10.5803846 | H | 37.9235737 | 8.1046745 | 10.1598784 |  |
| H | 37.3087610 | 6.4321044 | 9.8557474 | H | 37.6478510 | 7.5271681 | 12.4184163 |  |
| H | 37.9124990 | 8.0909904 | 10.1226671 | H | 42.4714544 | 4.6024812 | 11.1172795 |  |
| H | 37.8173530 | 7.6188854 | 12.4039447 | H | 43.5281539 | 5.0027432 | 12.4819257 |  |
| H | 42.5028692 | 4.6294619 | 11.1104332 | H | 42.0063854 | 4.0928543 | 12.7773235 |  |
| H | 43.5200087 | 4.9943987 | 12.5115868 | H | 40.2007165 | 7.1936084 | 13.1764526 |  |
| H | 41.9896031 | 4.0761680 | 12.7401777 | H | 40.4166105 | 5.5653388 | 13.7484933 |  |
| H | 40.1586189 | 7.1719517 | 13.1367422 | H | 55.3016728 | 3.9218052 | 14.7712109 |  |
| H | 40.3420215 | 5.5152759 | 13.6587361 | H | 53.6172752 | 4.2134743 | 14.2070211 |  |
| H | 55.2869769 | 3.9347181 | 14.7930848 | H | 55.2700576 | 1.7504055 | 13.3944813 |  |
| H | 53.6117223 | 4.2053994 | 14.1981315 | H | 55.1129583 | 3.2383286 | 12.4116178 |  |
| H | 55.3025513 | 1.7608946 | 13.4091022 | H | 51.9857047 | 3.1749431 | 15.3299007 |  |
| H | 55.1787527 | 3.2582233 | 12.4353382 | H | 39.4460844 | 10.7515931 | 14.6675873 |  |
| H | 51.9890337 | 3.2033745 | 15.3578529 | H | 41.6273657 | 9.5898379 | 14.5083191 |  |
| H | 39.5382668 | 10.6266295 | 14.8733332 | H | 40.9192113 | 8.0995867 | 15.2004938 |  |
| H | 41.2428582 | 9.1196554 | 14.2948063 | H | 41.2295298 | 9.0831947 | 17.4604102 |  |
| H | 40.6713146 | 7.8453891 | 15.3930344 | H | 41.3751193 | 10.7385845 | 16.7638796 |  |
| H | 41.4997161 | 8.9134196 | 17.3523914 | H | 45.0260215 | 7.0502484 | 15.2615587 |  |
| H | 41.6708954 | 10.5099418 | 16.5572870 | H | 43.2585781 | 6.8080916 | 15.5008895 |  |
| H | 44.5871097 | 7.2411389 | 15.1796434 | H | 43.8514609 | 7.6819079 | 14.0581342 |  |
| H | 42.9109657 | 6.8229482 | 15.7006693 | H | 44.3429380 | 7.7040288 | 17.8938903 |  |
| H | 43.1894139 | 7.7111410 | 14.1388191 | H | 44.0848455 | 9.4231183 | 18.2966237 |  |
| H | 43.8393749 | 7.7242895 | 17.9063376 | H | 46.4063205 | 8.8950568 | 18.7997160 |  |
| H | 43.7909539 | 9.4753803 | 18.3162699 | H | 45.7665928 | 10.8963633 | 16.7885544 |  |
| H | 46.0240968 | 8.6408437 | 18.7313953 | H | 46.7878360 | 9.7526137 | 15.1060363 |  |
| H | 45.6231999 | 10.8254741 | 16.8413408 | H | 48.7708828 | 8.4590807 | 17.0446848 |  |
| H | 46.5686913 | 9.5896731 | 15.1273657 | H | 51.7695852 | 5.1902405 | 16.7685779 |  |
| H | 48.3047035 | 8.1190164 | 17.1784280 | H | 46.7380255 | 7.9267182 | 13.7915735 |  |
| H | 51.5581634 | 5.1336621 | 16.8925375 | H | 38.8652233 | 12.8781331 | 15.4866909 |  |
| H | 46.5435819 | 7.7089557 | 13.7394226 | H | 38.9764617 | 9.0548078 | 17.0819871 |  |
| H | 38.6582924 | 12.5440478 | 15.7235189 | H | 38.9720713 | 10.7305153 | 16.9985369 |  |

| ***Lig_Hyd*** Reactant | ***Lig_Hyd*** Product | Fixed (f) |
| --- | --- | --- |

| H | 39.4879064 | 10.2854935 | 15.6620644 | H | 38.2293899 | 10.2571202 | 15.9201389 |  |
| --- | --- | --- | --- | --- | --- | --- | --- | --- |
| H | 41.3068251 | 9.3924584 | 14.2424822 | H | 40.7243456 | 10.4689390 | 15.6657819 |  |
| H | 41.1251799 | 7.7931016 | 15.0127569 | H | 40.6667548 | 9.0498638 | 14.5761204 |  |
| H | 41.5953271 | 8.8588013 | 17.2746692 | H | 40.8734926 | 7.5265591 | 16.5581575 |  |
| H | 42.0050726 | 10.4000744 | 16.4302888 | H | 41.1219221 | 8.9721556 | 17.5943027 |  |
| H | 44.5951287 | 6.6079888 | 16.3212466 | H | 44.9401551 | 6.4880845 | 16.2157348 |  |
| H | 42.8809526 | 6.6669194 | 16.9160173 | H | 43.2041945 | 6.4241368 | 16.6884725 |  |
| H | 43.2272358 | 6.6478210 | 15.1383832 | H | 43.6604350 | 6.4295098 | 14.9540042 |  |
| H | 48.4702396 | 8.4380739 | 16.8697002 | H | 48.6689028 | 8.5440584 | 16.7855420 |  |
| H | 51.6984771 | 5.4488000 | 16.4543898 | H | 51.8076193 | 5.4161107 | 16.4976498 |  |
| H | 46.3959036 | 9.5413017 | 14.8925769 | H | 46.4927761 | 9.5141015 | 14.8575814 |  |
| H | 45.7364142 | 11.1316080 | 16.5678555 | H | 45.7866408 | 11.1227154 | 16.4938171 |  |
| H | 46.2673610 | 9.3267122 | 18.7443499 | H | 46.2346362 | 9.4126729 | 18.7021622 |  |
| H | 45.9857901 | 7.3450982 | 14.1034555 | H | 46.1352732 | 7.3843732 | 14.1158200 |  |
| H | 44.0596347 | 8.3491685 | 18.4301411 | H | 44.1643196 | 8.2508774 | 18.2305210 |  |
| H | 43.9604896 | 10.1007595 | 18.0755544 | H | 43.9815713 | 9.9775365 | 17.8608113 |  |
| N | 39.1823038 | 8.4956496 | 16.7571454 | N | 39.0527189 | 8.5652759 | 17.0606702 |  |
| C | 39.5789235 | 9.1925810 | 15.5300280 | C | 38.8007510 | 9.3304466 | 15.7808325 |  |
| C | 38.7114234 | 8.8056285 | 14.2723862 | C | 38.0599066 | 8.3316367 | 14.8311007 |  |
| O | 37.8694208 | 7.8964292 | 14.4550565 | O | 37.8506852 | 8.7253921 | 13.6821564 |  |
| C | 41.0482852 | 8.8810185 | 15.1805431 | C | 40.3232860 | 9.4548714 | 15.5362465 |  |
| C | 41.9450883 | 9.3052598 | 16.3300069 | C | 40.5384837 | 8.5494683 | 16.7683340 |  |
| S | 43.6912381 | 8.7857726 | 16.0951039 | S | 43.8441244 | 8.6338713 | 15.8697400 |  |
| C | 43.5798410 | 6.9733041 | 16.1253499 | C | 43.9249282 | 6.8101451 | 15.9511329 |  |
| C | 44.3997813 | 9.1458220 | 17.7530939 | C | 44.4796671 | 9.0489215 | 17.5408036 |  |
| C | 45.9191650 | 9.2320092 | 17.6982798 | C | 45.9931729 | 9.2412661 | 17.6349438 |  |
| O | 46.4239699 | 8.0304500 | 17.1113505 | O | 46.6705394 | 8.0614396 | 17.1810044 |  |
| C | 46.5082588 | 10.4138345 | 16.8812217 | C | 46.5675345 | 10.4212512 | 16.8201522 |  |
| O | 47.4679797 | 11.1676442 | 17.5964496 | O | 47.5158219 | 11.1972576 | 17.5413069 |  |
| C | 47.1615569 | 9.7123972 | 15.6679221 | C | 47.2515511 | 9.7296495 | 15.6247281 |  |
| O | 48.2229491 | 10.4126847 | 15.1051504 | O | 48.2853302 | 10.4711663 | 15.0512416 |  |
| C | 47.5402157 | 8.3486083 | 16.2787231 | C | 47.7024023 | 8.4017623 | 16.2666589 |  |
| O | 38.9919503 | 9.4228070 | 13.2233410 | O | 37.8008846 | 7.2221781 | 15.3819707 |  |
| N | 50.7000315 | 4.5716770 | 14.8721053 | N | 50.7905655 | 4.5258038 | 14.9345628 |  |
| C | 50.7917404 | 5.4940960 | 15.8388540 | C | 50.9031574 | 5.4700435 | 15.8791440 |  |
| N | 49.9200481 | 6.4600279 | 16.1456328 | N | 50.0561766 | 6.4635473 | 16.1611645 |  |
| C | 48.8531260 | 6.4355843 | 15.3431019 | C | 48.9870573 | 6.4479275 | 15.3586847 |  |
| C | 48.6119812 | 5.5380725 | 14.2960299 | C | 48.7278621 | 5.5307077 | 14.3318328 |  |
| C | 49.6156848 | 4.5588293 | 14.0713139 | C | 49.7071873 | 4.5231170 | 14.1330555 |  |
| N | 49.5182103 | 3.6339888 | 13.1005986 | N | 49.5870527 | 3.5769613 | 13.1830575 |  |
| N | 47.4057089 | 5.8126823 | 13.6789500 | N | 47.5273047 | 5.8158808 | 13.7085687 |  |
| C | 46.9307596 | 6.8473076 | 14.3226824 | C | 47.0737288 | 6.8740999 | 14.3326487 |  |
| N | 47.7590951 | 7.2838441 | 15.3483339 | N | 47.9120540 | 7.3173955 | 15.3449185 |  |
| H | 38.5970934 | 7.7199975 | 16.4180433 | H | 48.7812271 | 3.5657044 | 12.5715830 |  |
| H | 38.5257716 | 9.0844846 | 17.2713805 | H | 50.3132025 | 2.8798713 | 13.0721186 |  |
| H | 48.7155372 | 3.6195882 | 12.4846729 | H | 48.6251018 | 11.0529601 | 15.7537132 |  |
| H | 50.2662674 | 2.9642056 | 12.9667928 | H | 48.0680441 | 10.6181006 | 18.0882660 |  |
| H | 48.5724619 | 11.0014733 | 15.7968878 | H | 38.7540451 | 8.9820474 | 17.9456972 |  |
| H | 48.0451906 | 10.5810600 | 18.1095381 | H | 38.5316130 | 7.6575760 | 16.8280619 |  |

| ***pLig_Hyd*** Reactant | ***pLig_Hyd*** Product | Fixed (f) |
| --- | --- | --- |

| H | 39.5599277 | 10.4307159 | 15.8168509 | H | 38.3297459 | 10.2543801 | 15.9867115 |
| --- | --- | --- | --- | --- | --- | --- | --- |
| H | 41.2170128 | 9.1116550 | 14.4850814 | H | 40.7725797 | 10.3857798 | 15.5538887 |
| H | 40.7546967 | 7.6413710 | 15.3613458 | H | 40.7008118 | 8.8580890 | 14.6240341 |
| H | 41.5560524 | 8.6602721 | 17.5416130 | H | 40.9801807 | 7.5567329 | 16.7285343 |
| H | 41.9065778 | 10.1955408 | 16.6865781 | H | 41.0684837 | 9.0942016 | 17.6562360 |
| H | 44.5018727 | 6.4290701 | 16.2746868 | H | 44.9415801 | 6.5219988 | 16.1479523 |
| H | 42.9142231 | 6.4564898 | 17.1514743 | H | 43.2366728 | 6.3949611 | 16.7020122 |
| H | 42.9630343 | 6.4168530 | 15.3346729 | H | 43.6053947 | 6.4781447 | 14.9464628 |
| H | 48.4140880 | 8.4495656 | 16.7639601 | H | 48.6455092 | 8.5590187 | 16.7625205 |
| H | 51.4821964 | 5.2736115 | 16.9607103 | H | 51.7746909 | 5.4121134 | 16.5596544 |
| H | 46.5924445 | 9.5747592 | 14.5641428 | H | 46.4375419 | 9.4998604 | 14.8510848 |
| H | 45.5018424 | 10.9100152 | 16.1420020 | H | 45.7452029 | 11.1010393 | 16.4968730 |
| H | 46.4180108 | 9.2393469 | 18.4221050 | H | 46.2772890 | 9.3829439 | 18.6942558 |
| H | 46.4554068 | 7.4898124 | 13.5544914 | H | 46.1630858 | 7.3927811 | 14.0468139 |
| H | 44.2222112 | 8.2213154 | 18.4476038 | H | 44.1958292 | 8.2089628 | 18.2757830 |
| H | 44.0042845 | 9.9599993 | 18.0717098 | H | 43.9933356 | 9.9454843 | 17.9555359 |
| N | 38.6894667 | 8.8327369 | 16.8184023 | N | 39.0603533 | 8.5157355 | 17.0589425 |
| C | 39.4533814 | 9.3392624 | 15.6838914 | C | 38.8295891 | 9.2954203 | 15.7911467 |
| C | 38.6042098 | 9.1941014 | 14.3961915 | C | 38.0291169 | 8.4361103 | 14.8175626 |
| O | 37.3140300 | 9.0207775 | 14.6440118 | O | 37.7782773 | 8.9675164 | 13.6309904 |
| C | 40.8499184 | 8.7367045 | 15.4520426 | C | 40.3644573 | 9.3688799 | 15.5347996 |
| C | 41.8210171 | 9.1041592 | 16.5698099 | C | 40.5730967 | 8.5672487 | 16.8344148 |
| S | 43.5332151 | 8.5794769 | 16.1677999 | S | 43.7875108 | 8.6523898 | 15.9373604 |
| C | 43.4585341 | 6.7680131 | 16.2493319 | C | 43.9081586 | 6.8280424 | 15.9440246 |
| C | 44.4323824 | 9.0126220 | 17.7140815 | C | 44.4822302 | 9.0265790 | 17.5962825 |
| C | 45.9214860 | 9.1566672 | 17.4345003 | C | 45.9972746 | 9.2210603 | 17.6348573 |
| O | 46.3703718 | 7.9973418 | 16.7479924 | O | 46.6507123 | 8.0456346 | 17.1423107 |
| C | 46.3611222 | 10.3782251 | 16.5761004 | C | 46.5415000 | 10.4105717 | 16.8098653 |
| O | 47.0771558 | 11.3492061 | 17.3037594 | O | 47.4885744 | 11.2010225 | 17.5126761 |
| C | 47.2286359 | 9.7365463 | 15.4522907 | C | 47.2107366 | 9.7264310 | 15.6009851 |
| O | 48.3344189 | 10.5027944 | 15.0988243 | O | 48.2205606 | 10.4787168 | 15.0030055 |
| C | 47.5742116 | 8.3575023 | 16.0524063 | C | 47.6856862 | 8.4039232 | 16.2350816 |
| O | 39.0695872 | 9.2668401 | 13.2841487 | O | 37.6827360 | 7.3188961 | 15.1124510 |
| N | 50.7936280 | 4.4847416 | 15.1802692 | N | 50.7884280 | 4.5212692 | 14.9774515 |
| C | 50.7239497 | 5.3768878 | 16.1747768 | C | 50.8847446 | 5.4678315 | 15.9207790 |
| N | 49.8477666 | 6.3749387 | 16.3280708 | N | 50.0355678 | 6.4665146 | 16.1795902 |
| C | 48.9704047 | 6.4204899 | 15.3238784 | C | 48.9857402 | 6.4513408 | 15.3533136 |
| C | 48.9116489 | 5.5623180 | 14.2209879 | C | 48.7441772 | 5.5317904 | 14.3246281 |
| C | 49.8990093 | 4.5420318 | 14.1738109 | C | 49.7241432 | 4.5193489 | 14.1507781 |
| N | 49.9680489 | 3.6442377 | 13.1763223 | N | 49.6223829 | 3.5692504 | 13.2035512 |
| N | 47.8692958 | 5.9084544 | 13.3811535 | N | 47.5571016 | 5.8192954 | 13.6760147 |
| C | 47.3146716 | 6.9464854 | 13.9486577 | C | 47.0949664 | 6.8809414 | 14.2868895 |
| N | 47.9325730 | 7.3221283 | 15.1359511 | N | 47.9142575 | 7.3258210 | 15.3139862 |
| H | 38.8553754 | 9.3394493 | 17.6856768 | H | 38.7151315 | 8.9514713 | 17.9214751 |
| H | 38.8388132 | 7.8369582 | 16.9873476 | H | 38.6322783 | 7.5761634 | 16.9771115 |
| H | 37.2516221 | 8.9529819 | 15.6330709 | H | 38.0963244 | 9.8820469 | 13.5419887 |
| H | 49.3185074 | 3.6824692 | 12.4013676 | H | 48.8408977 | 3.5676535 | 12.5611572 |
| H | 50.7028303 | 2.9467333 | 13.1799140 | H | 50.3567001 | 2.8788151 | 13.1030694 |
| H | 48.4039063 | 11.2221962 | 15.7521935 | H | 48.5740160 | 11.0661363 | 15.6937914 |
| H | 47.7021815 | 10.9243530 | 17.9123833 | H | 48.0717973 | 10.6324635 | 18.0384590 |

| ***Lig_Wat*** Reactant | ***Lig_Wat*** Product | Fixed (f) |
| --- | --- | --- |

| H | 39.6357430 | 10.4221405 | 15.4685731 | H | 38.5420380 | 10.4045404 | 15.1154910 |  |
| --- | --- | --- | --- | --- | --- | --- | --- | --- |
| H | 41.3688266 | 9.1653423 | 14.2713959 | H | 41.0306277 | 10.2743193 | 15.0434278 |  |
| H | 40.9459209 | 7.6801908 | 15.1667559 | H | 40.8917622 | 8.6499472 | 14.3003327 |  |
| H | 41.5511827 | 8.7785099 | 17.3410433 | H | 40.7654776 | 7.6242397 | 16.5799755 |  |
| H | 41.9941434 | 10.2857786 | 16.4658191 | H | 41.0361047 | 9.2489719 | 17.2940794 |  |
| H | 44.5145560 | 6.4512478 | 16.3895072 | H | 44.9221251 | 6.3438653 | 16.4873670 |  |
| H | 42.8372447 | 6.5468581 | 17.0666039 | H | 43.1921454 | 6.3576773 | 16.9880216 |  |
| H | 43.0942144 | 6.4774538 | 15.2707571 | H | 43.6203713 | 6.2726723 | 15.2481749 |  |
| H | 48.4245913 | 8.3979284 | 16.8985782 | H | 48.7108783 | 8.5042828 | 16.8721487 |  |
| H | 51.9180737 | 5.7369988 | 16.2973348 | H | 51.9514855 | 5.5100838 | 16.2659699 |  |
| H | 46.3637016 | 9.4069770 | 14.8622580 | H | 46.4800763 | 9.4601392 | 15.0063854 |  |
| H | 45.6143215 | 10.9880765 | 16.4960152 | H | 45.7954902 | 11.0624563 | 16.6596119 |  |
| H | 46.2538873 | 9.2468041 | 18.7206814 | H | 46.2967956 | 9.3630745 | 18.8588640 |  |
| H | 45.9509871 | 7.0921217 | 14.2208327 | H | 46.0372894 | 7.2914948 | 14.3750924 |  |
| H | 44.0531680 | 8.2328036 | 18.4823609 | H | 44.2352815 | 8.1799379 | 18.4301680 |  |
| H | 43.9181796 | 9.9786274 | 18.1076729 | H | 44.0212929 | 9.8953759 | 18.0340333 |  |
| N | 39.0085308 | 8.8109803 | 16.7192464 | N | 39.0121266 | 8.8857616 | 16.6072672 |  |
| C | 39.5722143 | 9.3156619 | 15.4661759 | C | 38.9752949 | 9.3988383 | 15.1863501 |  |
| C | 38.6743901 | 8.9610003 | 14.2292140 | C | 38.1903632 | 8.3881119 | 14.3070035 |  |
| O | 37.5434090 | 8.4757655 | 14.4783861 | O | 37.6884184 | 7.4149520 | 14.9269367 |  |
| C | 40.9975036 | 8.7801063 | 15.2318303 | C | 40.5197086 | 9.3048360 | 15.0988735 |  |
| C | 41.9017538 | 9.1918795 | 16.3833520 | C | 40.5123172 | 8.6883215 | 16.5132964 |  |
| S | 43.6302263 | 8.6303712 | 16.1491515 | S | 43.8861526 | 8.5074297 | 16.0681436 |  |
| C | 43.4950840 | 6.8240239 | 16.2315893 | C | 43.9126823 | 6.6870632 | 16.2240068 |  |
| C | 44.3625680 | 9.0258527 | 17.7874688 | C | 44.5309059 | 8.9703339 | 17.7239805 |  |
| C | 45.8753206 | 9.1373302 | 17.6878159 | C | 46.0416053 | 9.1860821 | 17.7971860 |  |
| O | 46.3862675 | 7.9354855 | 17.0978125 | O | 46.7321238 | 8.0100021 | 17.3382865 |  |
| C | 46.4170992 | 10.3193786 | 16.8382690 | C | 46.5894670 | 10.3683157 | 16.9688067 |  |
| O | 47.3373612 | 11.1369849 | 17.5343197 | O | 47.5510895 | 11.1516773 | 17.6636653 |  |
| C | 47.1090491 | 9.6118549 | 15.6489726 | C | 47.2510995 | 9.6799045 | 15.7595476 |  |
| O | 48.1667025 | 10.3304720 | 15.0966517 | O | 48.2714753 | 10.4270898 | 15.1644803 |  |
| C | 47.5102671 | 8.2695940 | 16.2911017 | C | 47.7264093 | 8.3554547 | 16.3911457 |  |
| O | 39.1649640 | 9.2073927 | 13.1038853 | O | 38.1671272 | 8.6454292 | 13.0914359 |  |
| N | 50.9346098 | 4.7677110 | 14.7599448 | N | 50.8496207 | 4.6040286 | 14.7705787 |  |
| C | 50.9838736 | 5.6976020 | 15.7233779 | C | 50.9992761 | 5.5373257 | 15.7212698 |  |
| N | 50.0429875 | 6.5800420 | 16.0709571 | N | 50.1398594 | 6.4918915 | 16.0873874 |  |
| C | 48.9442757 | 6.4564337 | 15.3185926 | C | 49.0096221 | 6.4465928 | 15.3711825 |  |
| C | 48.7412759 | 5.5372174 | 14.2815765 | C | 48.7066639 | 5.5338253 | 14.3520931 |  |
| C | 49.8192209 | 4.6528605 | 14.0076352 | C | 49.7051061 | 4.5686386 | 14.0547933 |  |
| N | 49.7646602 | 3.7229904 | 13.0409852 | N | 49.5480785 | 3.6376396 | 13.0993109 |  |
| N | 47.4874716 | 5.6973650 | 13.7217005 | N | 47.4506582 | 5.7806869 | 13.8299074 |  |
| C | 46.9485149 | 6.6861181 | 14.3907388 | C | 47.0076385 | 6.8126637 | 14.5064743 |  |
| N | 47.7781504 | 7.1969347 | 15.3766363 | N | 47.9052113 | 7.2720735 | 15.4561060 |  |
| H | 38.0757840 | 8.4651774 | 16.4695028 | H | 48.6965955 | 3.5991168 | 12.5532492 |  |
| H | 38.8563631 | 9.5758769 | 17.3762550 | H | 50.2859592 | 2.9681112 | 12.9142164 |  |
| H | 48.9392986 | 3.6269492 | 12.4627317 | H | 48.6342843 | 10.9956535 | 15.8676859 |  |
| H | 50.5610135 | 3.1194557 | 12.8711326 | H | 48.1108532 | 10.5811181 | 18.2130756 |  |
| H | 48.4857558 | 10.9329836 | 15.7929418 | H | 38.6850108 | 9.5170346 | 17.3440853 |  |
| H | 47.9142893 | 10.5952888 | 18.0958609 | H | 38.4392644 | 8.0152764 | 16.5990064 |  |

| ***pLig_Wat*** Reactant | ***pLig_Wat*** Product | Fixed (f) |
| --- | --- | --- |

| H | 39.6595601 | 10.3915745 | 15.4958309 | H | 38.6685450 | 10.3669712 | 15.3114375 |  |
| --- | --- | --- | --- | --- | --- | --- | --- | --- |
| H | 41.4551631 | 9.1394521 | 14.3082513 | H | 41.1020404 | 10.1301337 | 14.9796019 |  |
| H | 40.9938495 | 7.6508378 | 15.1613932 | H | 40.8883549 | 8.4288655 | 14.4543309 |  |
| H | 41.5613558 | 8.7417719 | 17.3852984 | H | 40.9479947 | 7.7338553 | 16.8505592 |  |
| H | 41.9804156 | 10.2554133 | 16.5212698 | H | 40.8745093 | 9.4571430 | 17.3722922 |  |
| H | 44.5560872 | 6.4614554 | 16.4801571 | H | 44.9368961 | 6.3620512 | 16.4971618 |  |
| H | 42.8890175 | 6.5508752 | 17.1835500 | H | 43.2123850 | 6.3487687 | 17.0128576 |  |
| H | 43.1187525 | 6.4431250 | 15.3844364 | H | 43.6262581 | 6.2686423 | 15.2687618 |  |
| H | 48.4209288 | 8.4884574 | 16.8404815 | H | 48.6985641 | 8.5128334 | 16.8520872 |  |
| H | 51.8193666 | 5.6822932 | 16.3294104 | H | 51.9565569 | 5.5449790 | 16.2439898 |  |
| H | 46.3000917 | 9.4664130 | 14.8499508 | H | 46.4513493 | 9.4515933 | 14.9962633 |  |
| H | 45.5657683 | 11.0437518 | 16.4875361 | H | 45.7814154 | 11.0648917 | 16.6442553 |  |
| H | 46.2850219 | 9.3385103 | 18.7162121 | H | 46.3021686 | 9.3758231 | 18.8479635 |  |
| H | 45.9160344 | 7.2013991 | 14.1831232 | H | 46.0227785 | 7.2751433 | 14.3667742 |  |
| H | 44.0930221 | 8.2921774 | 18.5432102 | H | 44.2314513 | 8.2000766 | 18.4467825 |  |
| H | 43.9151215 | 10.0272962 | 18.1297658 | H | 44.0215908 | 9.9124937 | 18.0351307 |  |
| N | 38.8520717 | 8.7855741 | 16.5498674 | N | 39.0074627 | 8.6899146 | 16.6068685 |  |
| C | 39.6119791 | 9.2916971 | 15.4104232 | C | 39.0291718 | 9.3318609 | 15.2257625 |  |
| C | 38.7962682 | 9.0435131 | 14.1237276 | C | 38.2158638 | 8.6152662 | 14.1557078 |  |
| O | 37.5261834 | 8.7576129 | 14.3691795 | O | 36.9604220 | 8.2885750 | 14.4690688 |  |
| C | 41.0391673 | 8.7492960 | 15.2484612 | C | 40.5611824 | 9.1963108 | 15.1661432 |  |
| C | 41.9090957 | 9.1612913 | 16.4297619 | C | 40.5385726 | 8.7245810 | 16.6313352 |  |
| S | 43.6417444 | 8.6218968 | 16.2043887 | S | 43.8674841 | 8.5084454 | 16.0843713 |  |
| C | 43.5290329 | 6.8168607 | 16.3311618 | C | 43.9203211 | 6.6886196 | 16.2416452 |  |
| C | 44.3756949 | 9.0764105 | 17.8273679 | C | 44.5256342 | 8.9824264 | 17.7311811 |  |
| C | 45.8843466 | 9.2121595 | 17.6938161 | C | 46.0375828 | 9.1944789 | 17.7892664 |  |
| O | 46.3940786 | 8.0115570 | 17.1023067 | O | 46.7224108 | 8.0158947 | 17.3284424 |  |
| C | 46.3872505 | 10.3921224 | 16.8179391 | C | 46.5783982 | 10.3729219 | 16.9508156 |  |
| O | 47.3089697 | 11.2328599 | 17.4820610 | O | 47.5443347 | 11.1601663 | 17.6346777 |  |
| C | 47.0610184 | 9.6825110 | 15.6183601 | C | 47.2299000 | 9.6781993 | 15.7397275 |  |
| O | 48.0976922 | 10.4092197 | 15.0380003 | O | 48.2423660 | 10.4227034 | 15.1289199 |  |
| C | 47.4919532 | 8.3486792 | 16.2589515 | C | 47.7128326 | 8.3586432 | 16.3752756 |  |
| O | 39.2547748 | 9.1323169 | 13.0060345 | O | 38.6769236 | 8.3644277 | 13.0729338 |  |
| N | 50.8132882 | 4.7186281 | 14.8029135 | N | 50.8545194 | 4.6224896 | 14.7588061 |  |
| C | 50.8886088 | 5.6646896 | 15.7486709 | C | 51.0014003 | 5.5615419 | 15.7041455 |  |
| N | 49.9765245 | 6.5860490 | 16.0719024 | N | 50.1358595 | 6.5106152 | 16.0702819 |  |
| C | 48.8800071 | 6.4864253 | 15.3132145 | C | 49.0030951 | 6.4533159 | 15.3593643 |  |
| C | 48.6521505 | 5.5553540 | 14.2922859 | C | 48.7030913 | 5.5345052 | 14.3449046 |  |
| C | 49.7001597 | 4.6282903 | 14.0437764 | C | 49.7071499 | 4.5747426 | 14.0486593 |  |
| N | 49.6201914 | 3.6827133 | 13.0944645 | N | 49.5525738 | 3.6368562 | 13.0993476 |  |
| N | 47.4076683 | 5.7471916 | 13.7212303 | N | 47.4438434 | 5.7710181 | 13.8255969 |  |
| C | 46.8982574 | 6.7655108 | 14.3681556 | C | 46.9960043 | 6.8025890 | 14.4993596 |  |
| N | 47.7397435 | 7.2683780 | 15.3486609 | N | 47.8927951 | 7.2712133 | 15.4455008 |  |
| H | 38.9502438 | 9.3608718 | 17.3842544 | H | 38.5384904 | 9.2253992 | 17.3465901 |  |
| H | 39.1056056 | 7.8263917 | 16.7922037 | H | 38.6226478 | 7.7376139 | 16.6341919 |  |
| H | 37.4811937 | 8.7157813 | 15.3686697 | H | 36.6638013 | 8.6510443 | 15.3211270 |  |
| H | 48.7922535 | 3.5981283 | 12.5180566 | H | 48.6982280 | 3.5876200 | 12.5586980 |  |
| H | 50.3918959 | 3.0418348 | 12.9491738 | H | 50.2941752 | 2.9709608 | 12.9161209 |  |
| H | 48.4200981 | 11.0265047 | 15.7196918 | H | 48.6153992 | 10.9937433 | 15.8245070 |  |
| H | 47.9108310 | 10.7079753 | 18.0332891 | H | 48.1092360 | 10.5925959 | 18.1818346 |  |

| ***VioH_WT*** Reactant | ***VioH_WT*** Product | Fixed (f) |
| --- | --- | --- |

| C | 49.6530000 | 9.5620000 | 11.6920000 | C | 49.6530000 | 9.5620000 | 11.6920000 | f |
| --- | --- | --- | --- | --- | --- | --- | --- | --- |
| C | 48.2847676 | 8.9716428 | 11.8640155 | C | 48.2806518 | 8.9776904 | 11.8234347 |  |
| C | 47.1585926 | 9.7472747 | 12.1670150 | C | 47.1957975 | 9.7093110 | 12.3226797 |  |
| C | 48.1227440 | 7.5851175 | 11.7117281 | C | 48.0778731 | 7.6351577 | 11.4704783 |  |
| C | 45.8869481 | 9.1701016 | 12.3022699 | C | 45.9290891 | 9.1312239 | 12.4749290 |  |
| C | 46.8711282 | 7.0026218 | 11.8183521 | C | 46.8291183 | 7.0509726 | 11.6039242 |  |
| C | 45.7520000 | 7.7950000 | 12.1120000 | C | 45.7520000 | 7.7950000 | 12.1120000 | f |
| O | 44.5738946 | 7.1045593 | 12.2017078 | O | 44.5819004 | 7.1056667 | 12.1789893 |  |
| C | 41.9040000 | 14.7280000 | 14.4270000 | C | 41.9040000 | 14.7280000 | 14.4270000 | f |
| C | 41.9459783 | 14.9061026 | 15.9537341 | C | 41.8979691 | 14.9653946 | 15.9459129 |  |
| O | 41.0218970 | 14.4560423 | 16.6589043 | O | 40.8939226 | 14.6664493 | 16.6221462 |  |
| C | 42.1180637 | 13.2596343 | 14.0117399 | C | 42.0471603 | 13.2480893 | 14.0350126 |  |
| C | 41.6940000 | 12.9630000 | 12.5580000 | C | 41.6940000 | 12.9630000 | 12.5580000 | f |
| C | 42.2990461 | 11.6387368 | 12.0724552 | C | 42.1571197 | 11.5494337 | 12.1766035 |  |
| C | 40.1662146 | 12.9506873 | 12.4013340 | C | 40.1887971 | 13.1229202 | 12.2951750 |  |
| N | 42.9823573 | 15.5482935 | 16.5243968 | N | 42.9798730 | 15.5122261 | 16.5322586 |  |
| C | 43.1070000 | 15.8350000 | 17.9710000 | C | 43.1070000 | 15.8350000 | 17.9710000 | f |
| C | 44.2300000 | 15.0460000 | 18.6750000 | C | 44.2300000 | 15.0460000 | 18.6750000 | f |
| C | 45.6007817 | 15.2514492 | 18.0135004 | C | 45.5955227 | 15.2334929 | 17.9942928 |  |
| C | 43.8198065 | 13.5843043 | 18.8597599 | C | 43.8160769 | 13.5922813 | 18.8850126 |  |
| C | 46.7774633 | 14.9078506 | 18.9301714 | C | 46.7837362 | 14.9339803 | 18.9118228 |  |
| C | 48.5070000 | 10.5290000 | 19.6580000 | C | 48.5070000 | 10.5290000 | 19.6580000 | f |
| C | 47.2541195 | 10.0111096 | 18.9702552 | C | 47.3284916 | 9.9956669 | 18.8701293 |  |
| S | 46.1778618 | 11.2786277 | 18.1997788 | S | 46.2223929 | 11.3126748 | 18.2535767 |  |
| C | 47.2860000 | 12.0880000 | 16.9850000 | C | 47.2860000 | 12.0880000 | 16.9850000 | f |
| C | 45.3810000 | 11.5260000 | 22.9720000 | C | 45.3810000 | 11.5260000 | 22.9720000 | f |
| C | 44.5146553 | 10.6978326 | 22.0373257 | C | 44.5807623 | 10.7073506 | 21.9732268 |  |
| N | 44.7815853 | 9.3807627 | 21.9600249 | N | 44.8964128 | 9.4100192 | 21.8388551 |  |
| O | 43.6215615 | 11.2378278 | 21.3722697 | O | 43.6861349 | 11.2464273 | 21.3005254 |  |
| C | 51.1580000 | 5.4550000 | 18.9390000 | C | 51.1580000 | 5.4550000 | 18.9390000 | f |
| C | 50.1320489 | 5.9961098 | 17.9205008 | C | 50.4593346 | 6.2678551 | 17.8422819 |  |
| O | 50.3614704 | 5.7590798 | 16.7072493 | O | 50.7533707 | 5.9693791 | 16.6559181 |  |
| O | 49.1470383 | 6.6605380 | 18.3593523 | O | 49.6535616 | 7.1737406 | 18.1987843 |  |
| C | 50.6390000 | -1.7230000 | 16.1830000 | C | 50.6390000 | -1.7230000 | 16.1830000 | f |
| C | 49.8940344 | -2.6259812 | 17.1865835 | C | 49.8902749 | -2.6293506 | 17.1815300 |  |
| O | 50.4492756 | -3.5838366 | 17.7235301 | O | 50.4437195 | -3.5926730 | 17.7111532 |  |
| N | 48.5761523 | -2.3413355 | 17.3871964 | N | 48.5748478 | -2.3404555 | 17.3876220 |  |
| C | 47.6540000 | -3.2120000 | 18.1260000 | C | 47.6540000 | -3.2120000 | 18.1260000 | f |
| C | 47.6709509 | -4.6863769 | 17.5955115 | C | 47.6815136 | -4.6896568 | 17.6023245 |  |
| O | 47.4243848 | -5.6138517 | 18.3694585 | O | 47.4630737 | -5.6175086 | 18.3845108 |  |
| C | 46.2382843 | -2.6423832 | 18.0478951 | C | 46.2355638 | -2.6502102 | 18.0369458 |  |
| N | 47.9097465 | -4.8413460 | 16.2808253 | N | 47.8964282 | -4.8447998 | 16.2838260 |  |
| C | 48.0570000 | -6.1290000 | 15.5940000 | C | 48.0570000 | -6.1290000 | 15.5940000 | f |
| C | 48.5074957 | -5.9027747 | 14.1463154 | C | 48.5132564 | -5.8961526 | 14.1490784 |  |
| C | 47.5244230 | -5.1247220 | 13.2545979 | C | 47.5230641 | -5.1382310 | 13.2476975 |  |
| C | 47.5152155 | -3.5747595 | 13.3357631 | C | 47.4799297 | -3.5887108 | 13.3290608 |  |
| O | 48.2039859 | -3.0232410 | 14.2624685 | O | 48.1246783 | -3.0252533 | 14.2798943 |  |
| O | 46.8221597 | -2.9709879 | 12.5013499 | O | 46.8024749 | -2.9979648 | 12.4733213 |  |
| C | 38.8100000 | 8.1240000 | 20.3900000 | C | 38.8100000 | 8.1240000 | 20.3900000 | f |
| C | 38.8818500 | 7.3266310 | 19.0703467 | C | 38.7544917 | 7.3880473 | 19.0371922 |  |
| O | 39.4079905 | 7.7796493 | 18.0559996 | O | 39.0741861 | 7.9346858 | 17.9785883 |  |
| C | 39.7343841 | 9.3330548 | 20.4337275 | C | 39.7614543 | 9.3170541 | 20.3954485 |  |
| C | 39.1781459 | 10.6048749 | 19.8357611 | C | 39.2085748 | 10.5964772 | 19.8046278 |  |
| C | 37.8919289 | 10.6881961 | 19.2842599 | C | 37.9830232 | 10.6477181 | 19.1214221 |  |
| C | 39.9896997 | 11.7533059 | 19.8373543 | C | 39.9362220 | 11.7878188 | 19.9678600 |  |
| C | 37.4096958 | 11.8933098 | 18.7653602 | C | 37.4871978 | 11.8538828 | 18.6208201 |  |
| C | 39.5136945 | 12.9563052 | 19.3199577 | C | 39.4437047 | 12.9945690 | 19.4682976 |  |
| C | 38.2190000 | 13.0260000 | 18.7930000 | C | 38.2190000 | 13.0260000 | 18.7930000 | f |
| N | 38.3464132 | 6.0653141 | 19.0716158 | N | 38.3353508 | 6.0925282 | 19.0532520 |  |
| C | 38.1120000 | 5.2860000 | 17.8400000 | C | 38.1120000 | 5.2860000 | 17.8400000 | f |
| C | 39.4217268 | 4.8526981 | 17.1228989 | C | 39.4337307 | 4.9387544 | 17.0923967 |  |
| O | 39.3947213 | 4.6203521 | 15.9143055 | O | 39.4245009 | 4.8331378 | 15.8675909 |  |
| C | 37.2086058 | 6.0476227 | 16.8639659 | C | 37.1097879 | 5.9270195 | 16.8818068 |  |
| C | 36.6277375 | 5.1771526 | 15.7600973 | C | 36.5599275 | 4.9357939 | 15.8653259 |  |
| N | 36.6895324 | 5.7180127 | 14.5199688 | N | 36.4591517 | 5.4086957 | 14.5997497 |  |
| O | 36.0704161 | 4.1107703 | 16.0080951 | O | 36.1678259 | 3.8230420 | 16.2055426 |  |
| C | 42.2260000 | 4.7200000 | 12.4680000 | C | 42.2260000 | 4.7200000 | 12.4680000 | f |
| C | 41.4263662 | 5.9878851 | 12.6997197 | C | 41.3795667 | 5.9727477 | 12.6473908 |  |
| N | 40.3582434 | 5.8618782 | 13.4996938 | N | 40.1625928 | 5.7779901 | 13.1955132 |  |
| O | 41.7551438 | 7.0772862 | 12.2013666 | O | 41.7670715 | 7.0941456 | 12.2925205 |  |
| C | 31.1480000 | 6.1300000 | 16.5410000 | C | 31.1480000 | 6.1300000 | 16.5410000 | f |
| C | 32.2504752 | 7.1354072 | 16.6647746 | C | 32.2447393 | 7.1406379 | 16.6586384 |  |
| C | 32.1123403 | 8.2563980 | 17.5008510 | C | 32.0999852 | 8.2713435 | 17.4798659 |  |
| C | 33.4513866 | 6.9700634 | 15.9652278 | C | 33.4523341 | 6.9647290 | 15.9742766 |  |
| C | 33.1429211 | 9.1831055 | 17.6487040 | C | 33.1324031 | 9.1955886 | 17.6279132 |  |
| C | 34.4822796 | 7.8964292 | 16.0840557 | C | 34.4871148 | 7.8851823 | 16.0987283 |  |
| C | 34.3370000 | 8.9970000 | 16.9390000 | C | 34.3370000 | 8.9970000 | 16.9390000 | f |
| O | 35.3425605 | 9.8977522 | 17.0865719 | O | 35.3396844 | 9.8965604 | 17.0982299 |  |
| C | 31.5110000 | 9.5750000 | 13.7680000 | C | 31.5110000 | 9.5750000 | 13.7680000 | f |
| C | 31.6196142 | 8.1512029 | 13.2363307 | C | 31.5997989 | 8.1522337 | 13.2298427 |  |
| N | 33.0052898 | 7.8322626 | 12.8973116 | N | 32.9805999 | 7.8280219 | 12.8801976 |  |
| C | 33.4045757 | 6.7036073 | 12.3080339 | C | 33.3838177 | 6.6811405 | 12.3325006 |  |
| N | 32.5137005 | 5.7581867 | 11.9740121 | N | 32.4978215 | 5.7185860 | 12.0362736 |  |
| N | 34.7047725 | 6.5274781 | 12.0418328 | N | 34.6861369 | 6.4990031 | 12.0776534 |  |
| C | 34.1070000 | 15.1680000 | 14.2080000 | C | 34.1070000 | 15.1680000 | 14.2080000 | f |
| C | 35.4225517 | 14.6534788 | 14.7072757 | C | 35.4257514 | 14.6515714 | 14.7003220 |  |
| C | 36.5320182 | 15.4917156 | 14.8802352 | C | 36.5443777 | 15.4843193 | 14.8412570 |  |
| C | 35.5773742 | 13.2819674 | 14.9647731 | C | 35.5723732 | 13.2854881 | 14.9896144 |  |
| C | 37.7795007 | 14.9814334 | 15.2597145 | C | 37.7915016 | 14.9756678 | 15.2239917 |  |
| C | 36.8037959 | 12.7591396 | 15.3427327 | C | 36.7995857 | 12.7647696 | 15.3708748 |  |
| C | 37.9210000 | 13.6020000 | 15.4640000 | C | 37.9210000 | 13.6020000 | 15.4640000 | f |
| O | 39.0931344 | 12.9988964 | 15.7407881 | O | 39.0971178 | 12.9981998 | 15.7629405 |  |
| C | 35.6640000 | 13.4960000 | 9.4900000 | C | 35.6640000 | 13.4960000 | 9.4900000 | f |
| C | 35.6761630 | 12.2728387 | 10.3620126 | C | 35.7058838 | 12.2647512 | 10.3496097 |  |
| C | 36.5340176 | 12.2204261 | 11.4722281 | C | 36.5140177 | 12.2338265 | 11.4970541 |  |
| C | 34.8364486 | 11.1763253 | 10.1209499 | C | 34.9173807 | 11.1404250 | 10.0648297 |  |
| C | 36.5433562 | 11.1210149 | 12.3211669 | C | 36.5099301 | 11.1376039 | 12.3544220 |  |
| C | 34.8253256 | 10.0631184 | 10.9702861 | C | 34.8940603 | 10.0341629 | 10.9187827 |  |
| C | 35.6730000 | 10.0490000 | 12.0800000 | C | 35.6730000 | 10.0490000 | 12.0800000 | f |
| O | 35.6377776 | 8.9915329 | 12.9608711 | O | 35.5547255 | 9.0081257 | 12.9656998 |  |
| C | 38.0320000 | 5.5450000 | 9.8330000 | C | 38.0320000 | 5.5450000 | 9.8330000 | f |
| C | 38.0641827 | 6.5790535 | 10.9622031 | C | 37.8728019 | 6.7225753 | 10.7965046 |  |
| N | 39.6099085 | 10.2817889 | 16.6247927 | N | 39.7977485 | 9.9859348 | 16.2827504 |  |
| C | 39.6891511 | 9.7779161 | 15.2626029 | C | 39.5333960 | 9.3292185 | 14.9450477 |  |
| C | 38.4600279 | 8.9201596 | 14.8423546 | C | 38.1509458 | 8.7479291 | 14.6807510 |  |
| O | 37.3071139 | 9.4306256 | 15.0593169 | O | 37.1365756 | 9.4110956 | 15.0656425 |  |
| C | 41.1082622 | 9.2106040 | 14.9565333 | C | 40.7201803 | 8.3796260 | 15.1725011 |  |
| C | 41.8447566 | 8.4954384 | 16.1044888 | C | 41.1717981 | 9.3270438 | 16.2887744 |  |
| S | 43.5863441 | 8.1201674 | 15.5265568 | S | 44.1949001 | 8.0455578 | 15.4904320 |  |
| C | 44.5936286 | 7.9240674 | 17.0649085 | C | 45.2465952 | 7.8639325 | 16.9746520 |  |
| C | 45.9411382 | 7.2050927 | 16.9387467 | C | 46.5957982 | 7.1922633 | 16.7988231 |  |
| O | 45.7391016 | 5.8073818 | 16.6600115 | O | 46.3925598 | 5.8073062 | 16.4793377 |  |
| C | 47.0254359 | 7.7138041 | 15.9269512 | C | 47.5340881 | 7.7746900 | 15.7063425 |  |
| O | 48.1303643 | 8.2875186 | 16.5496307 | O | 48.5772784 | 8.5507523 | 16.2269507 |  |
| C | 47.3949482 | 6.4212264 | 15.1302186 | C | 47.9923499 | 6.5021476 | 14.9120724 |  |
| O | 48.7003432 | 6.3185485 | 14.6959120 | O | 49.3141185 | 6.4776121 | 14.4979849 |  |
| C | 46.9484801 | 5.3041634 | 16.0858108 | C | 47.5786876 | 5.3486075 | 15.8513558 |  |
| O | 38.6175089 | 7.8290020 | 14.2575462 | O | 38.1145198 | 7.6917856 | 14.0251561 |  |
| N | 47.9230737 | 0.2879778 | 16.3709151 | N | 47.8578744 | 0.2732173 | 16.3966281 |  |
| C | 48.1089524 | 1.2812341 | 17.2501313 | C | 48.1791580 | 1.2796787 | 17.2217954 |  |
| N | 47.7889340 | 2.5669248 | 17.1224014 | N | 48.0695056 | 2.5884205 | 17.0073616 |  |
| C | 47.1878969 | 2.8097406 | 15.9495450 | C | 47.5441440 | 2.8467881 | 15.7994007 |  |
| C | 46.9015953 | 1.8736787 | 14.9459454 | C | 47.1253697 | 1.9041045 | 14.8502486 |  |
| C | 47.3420000 | 0.5300000 | 15.1700000 | C | 47.3420000 | 0.5300000 | 15.1700000 | f |
| N | 47.2033137 | -0.4554690 | 14.2859959 | N | 47.0709591 | -0.4767508 | 14.3356555 |  |
| N | 46.2633994 | 2.4848620 | 13.8818301 | N | 46.6277985 | 2.5356483 | 13.7259361 |  |
| C | 46.1788589 | 3.7474571 | 14.2207880 | C | 46.7472492 | 3.8166347 | 13.9843340 |  |
| N | 46.7237056 | 4.0232918 | 15.4673326 | N | 47.3120998 | 4.0790521 | 15.2198055 |  |
| N | 40.5409576 | 4.7065819 | 17.8605651 | N | 40.5336035 | 4.7104115 | 17.8413724 |  |
| C | 41.8290000 | 4.3160000 | 17.2800000 | C | 41.8290000 | 4.3160000 | 17.2800000 | f |
| C | 42.9623869 | 4.6451776 | 18.2448829 | C | 42.9598804 | 4.7551044 | 18.2033972 |  |
| O | 42.8689112 | 3.8996310 | 19.4504516 | O | 42.8606320 | 4.1495268 | 19.4860011 |  |
| C | 46.5740000 | 4.4500000 | 19.6650000 | C | 46.5740000 | 4.4500000 | 19.6650000 | f |
| C | 46.7855704 | 5.7003974 | 20.5134600 | C | 46.7833200 | 5.7013405 | 20.5119790 |  |
| O | 46.0589509 | 6.6992711 | 20.4019746 | O | 46.0582166 | 6.7007018 | 20.3987069 |  |
| N | 47.7762052 | 5.6616477 | 21.4367153 | N | 47.7893747 | 5.6710830 | 21.4200890 |  |
| C | 48.1100000 | 6.7810000 | 22.3140000 | C | 48.1100000 | 6.7810000 | 22.3140000 | f |
| O | 41.9157899 | 11.5972685 | 17.0816623 | O | 41.5793638 | 11.9769146 | 17.1655442 |  |
| O | 43.3967334 | 7.5214202 | 20.1126471 | O | 43.4706431 | 7.6813822 | 19.9722787 |  |
| O | 42.9704454 | 9.9702418 | 19.0096892 | O | 43.1060965 | 10.2862559 | 18.8426262 |  |
| C | 43.4181015 | 6.4081138 | 14.9452886 | C | 43.7631717 | 6.3127941 | 15.0995800 |  |
| H | 43.9402625 | 6.3530410 | 13.9856379 | H | 44.2944933 | 5.9835388 | 14.2007572 |  |
| H | 43.8756546 | 5.7434028 | 15.6794745 | H | 44.0497901 | 5.6732873 | 15.9368157 |  |
| H | 42.3490466 | 6.2210300 | 14.8242012 | H | 42.6812551 | 6.2586389 | 14.9253973 |  |
| H | 50.0752195 | 9.3149164 | 10.7030605 | H | 50.1136192 | 9.3100155 | 10.7222069 |  |
| H | 49.6458057 | 10.6589600 | 11.7907129 | H | 49.6497565 | 10.6591156 | 11.7928523 |  |
| H | 50.3506911 | 9.1624372 | 12.4483923 | H | 50.3135575 | 9.1573841 | 12.4799953 |  |
| H | 47.2702790 | 10.8273429 | 12.3021936 | H | 47.3424125 | 10.7541861 | 12.6135456 |  |
| H | 48.9927926 | 6.9573946 | 11.5039240 | H | 48.9158980 | 7.0419554 | 11.0942826 |  |
| H | 45.0120604 | 9.7786122 | 12.5460796 | H | 45.0946148 | 9.6983279 | 12.8925223 |  |
| H | 46.7302994 | 5.9286672 | 11.6787073 | H | 46.6507051 | 6.0130403 | 11.3135399 |  |
| H | 43.7459825 | 7.6096707 | 12.0910498 | H | 43.7730479 | 7.6174523 | 12.3561910 |  |
| H | 42.6361710 | 15.3841829 | 13.9295452 | H | 42.6921552 | 15.3277328 | 13.9429793 |  |
| H | 40.8992094 | 15.0629544 | 14.1259359 | H | 40.9354639 | 15.1178319 | 14.0781784 |  |
| H | 41.5690899 | 12.6010924 | 14.7029872 | H | 41.4076127 | 12.6338881 | 14.6869339 |  |
| H | 43.1847749 | 13.0130240 | 14.1489229 | H | 43.0829582 | 12.9292616 | 14.2404472 |  |
| H | 42.0983892 | 13.7703987 | 11.9163250 | H | 42.2404601 | 13.6869682 | 11.9235391 |  |
| H | 42.0105376 | 11.4252265 | 11.0303958 | H | 41.9150308 | 11.3130576 | 11.1279865 |  |
| H | 43.4006010 | 11.6575090 | 12.1205471 | H | 43.2451652 | 11.4281907 | 12.3077030 |  |
| H | 41.9508370 | 10.7947849 | 12.6895256 | H | 41.6630207 | 10.7894798 | 12.8073962 |  |
| H | 39.8769341 | 12.7782947 | 11.3521311 | H | 39.9424933 | 12.9011726 | 11.2442597 |  |
| H | 39.7189338 | 12.1492827 | 13.0109593 | H | 39.6088614 | 12.4335507 | 12.9325582 |  |
| H | 39.7002588 | 13.8943612 | 12.7264631 | H | 39.8272885 | 14.1404911 | 12.5131960 |  |
| H | 43.7154187 | 15.8962639 | 15.9155744 | H | 43.7624631 | 15.7560055 | 15.9344497 |  |
| H | 42.1332140 | 15.6138799 | 18.4259453 | H | 42.1324708 | 15.6297849 | 18.4324202 |  |
| H | 43.2931188 | 16.9183887 | 18.0675185 | H | 43.3017518 | 16.9183386 | 18.0499583 |  |
| H | 44.2858994 | 15.5065719 | 19.6810550 | H | 44.3031106 | 15.5198844 | 19.6734473 |  |
| H | 45.6918120 | 16.3074140 | 17.6951674 | H | 45.6781250 | 16.2769946 | 17.6362871 |  |
| H | 45.6515293 | 14.6467871 | 17.0909665 | H | 45.6436981 | 14.5958296 | 17.0940332 |  |
| H | 44.5022134 | 13.0355846 | 19.5186004 | H | 44.5314553 | 13.0421134 | 19.5056159 |  |
| H | 42.8166623 | 13.5197916 | 19.3110624 | H | 42.8419436 | 13.5388403 | 19.3968979 |  |
| H | 43.7924099 | 13.0476224 | 17.8999686 | H | 43.7173362 | 13.0678024 | 17.9237342 |  |
| H | 47.7415770 | 15.0665262 | 18.4206038 | H | 47.7409898 | 15.0801509 | 18.3861733 |  |
| H | 46.7658948 | 15.5433943 | 19.8320668 | H | 46.7762302 | 15.6042580 | 19.7881123 |  |
| H | 46.7349281 | 13.8565376 | 19.2530217 | H | 46.7558133 | 13.8962320 | 19.2772688 |  |
| H | 49.1720261 | 11.0473460 | 18.9477658 | H | 49.1190657 | 11.2189324 | 19.0553569 |  |
| H | 48.2734036 | 11.2297202 | 20.4780610 | H | 48.1876684 | 11.0608800 | 20.5699812 |  |
| H | 46.5868140 | 9.5017710 | 19.6851159 | H | 46.6927117 | 9.3390994 | 19.4855430 |  |
| H | 47.5321443 | 9.2937906 | 18.1915321 | H | 47.6820224 | 9.4213353 | 18.0048689 |  |
| H | 46.6457485 | 12.7153624 | 16.3492185 | H | 46.6405935 | 12.7020099 | 16.3423137 |  |
| H | 48.0416517 | 12.7216897 | 17.4711653 | H | 48.0517725 | 12.7270561 | 17.4458403 |  |
| H | 47.7814672 | 11.3269821 | 16.3612122 | H | 47.7628212 | 11.2994124 | 16.3818332 |  |
| H | 45.9298178 | 10.9225728 | 23.7091874 | H | 45.9532101 | 10.9104645 | 23.6804899 |  |
| H | 46.1054686 | 12.0871131 | 22.3585749 | H | 46.0784991 | 12.1678169 | 22.4087730 |  |
| H | 44.7428100 | 12.2581379 | 23.4861249 | H | 44.6916104 | 12.1849654 | 23.5185466 |  |
| H | 44.2291745 | 8.7625924 | 21.3524085 | H | 44.3580748 | 8.8030389 | 21.2033366 |  |
| H | 45.5582491 | 8.9621559 | 22.4556377 | H | 45.6564039 | 8.9834165 | 22.3536332 |  |
| H | 52.0842951 | 6.0483360 | 18.8615897 | H | 52.1981802 | 5.8067792 | 19.0467806 |  |
| H | 50.7679484 | 5.5255780 | 19.9646538 | H | 50.6387968 | 5.5705637 | 19.9019303 |  |
| H | 50.3207107 | -2.0310440 | 15.1735653 | H | 50.2885570 | -1.9917372 | 15.1727035 |  |
| H | 50.3795670 | -0.6618548 | 16.3054618 | H | 50.4165182 | -0.6576295 | 16.3422208 |  |
| H | 48.2155112 | -1.4761092 | 16.9560132 | H | 48.2059054 | -1.4733730 | 16.9636288 |  |
| H | 47.9699235 | -3.3084763 | 19.1785480 | H | 47.9641222 | -3.3027209 | 19.1809217 |  |
| H | 45.5406516 | -3.3007026 | 18.5868135 | H | 45.5383173 | -3.3092499 | 18.5757026 |  |
| H | 46.1941593 | -1.6371975 | 18.4967654 | H | 46.1837480 | -1.6423381 | 18.4786619 |  |
| H | 45.9136427 | -2.5668790 | 16.9974155 | H | 45.9168882 | -2.5810645 | 16.9843202 |  |
| H | 48.0963345 | -4.0100372 | 15.6925507 | H | 48.0564061 | -4.0109547 | 15.6892834 |  |
| H | 47.0990260 | -6.6830249 | 15.6142479 | H | 47.1041474 | -6.6925385 | 15.6068533 |  |
| H | 48.6821207 | -6.8975123 | 13.7012624 | H | 48.7088043 | -6.8882795 | 13.7067846 |  |
| H | 49.4800348 | -5.3815072 | 14.1530431 | H | 49.4767373 | -5.3582748 | 14.1624957 |  |
| H | 46.4865236 | -5.4541699 | 13.4473071 | H | 46.4905300 | -5.4892219 | 13.4321834 |  |
| H | 47.7083647 | -5.3628504 | 12.1933304 | H | 47.7207719 | -5.3734573 | 12.1883303 |  |
| H | 39.0793043 | 7.4221158 | 21.1979580 | H | 39.1207415 | 7.3950191 | 21.1562493 |  |
| H | 37.7565145 | 8.3884835 | 20.5774738 | H | 37.7820883 | 8.4225514 | 20.6575009 |  |
| H | 40.6954689 | 9.1002347 | 19.9438853 | H | 40.7024336 | 9.0447589 | 19.8858650 |  |
| H | 39.9855670 | 9.5361325 | 21.4888753 | H | 40.0491342 | 9.5208149 | 21.4401089 |  |
| H | 37.2499896 | 9.8094391 | 19.2118384 | H | 37.3915450 | 9.7481135 | 18.9512770 |  |
| H | 41.0061069 | 11.6836393 | 20.2349199 | H | 40.8980258 | 11.7599647 | 20.4909907 |  |
| H | 36.4188793 | 11.9213094 | 18.3077540 | H | 36.5367424 | 11.8581926 | 18.0846863 |  |
| H | 40.1585037 | 13.8382092 | 19.2948053 | H | 40.0225647 | 13.9143798 | 19.5891413 |  |
| H | 37.8555562 | 13.9638038 | 18.3647310 | H | 37.8456963 | 13.9674979 | 18.3822853 |  |
| H | 37.8762491 | 5.7494164 | 19.9131575 | H | 38.0438995 | 5.6963372 | 19.9401476 |  |
| H | 37.5991151 | 4.3582383 | 18.1387842 | H | 37.6900230 | 4.3261416 | 18.1766373 |  |
| H | 37.7474323 | 6.9064785 | 16.4537646 | H | 37.5547576 | 6.7991364 | 16.3964988 |  |
| H | 36.3470474 | 6.4256826 | 17.4362400 | H | 36.2472761 | 6.2712030 | 17.4737057 |  |
| H | 36.3391094 | 5.1540108 | 13.7546202 | H | 36.1394773 | 4.7594984 | 13.8897131 |  |
| H | 37.3147671 | 6.5120004 | 14.3214387 | H | 36.9883041 | 6.2389857 | 14.3117184 |  |
| H | 42.3070799 | 4.1120169 | 13.3820130 | H | 42.3027079 | 4.1572541 | 13.4116055 |  |
| H | 41.7197000 | 4.1024047 | 11.7074255 | H | 41.7551217 | 4.0575373 | 11.7237805 |  |
| H | 43.2247371 | 4.9803388 | 12.0959840 | H | 43.2270604 | 5.0068271 | 12.1251012 |  |
| H | 39.7938595 | 6.6878511 | 13.7475269 | H | 39.5698362 | 6.5835528 | 13.4154480 |  |
| H | 40.1608215 | 5.0162143 | 14.0275034 | H | 39.9604910 | 4.9386378 | 13.7288876 |  |
| H | 31.3273552 | 5.4305376 | 15.7092142 | H | 31.3066229 | 5.4545865 | 15.6853400 |  |
| H | 31.0428525 | 5.5218760 | 17.4575206 | H | 31.0786159 | 5.4950745 | 17.4428710 |  |
| H | 30.1699806 | 6.6120455 | 16.3703064 | H | 30.1607924 | 6.6065159 | 16.4155243 |  |
| H | 31.1773494 | 8.4030075 | 18.0509922 | H | 31.1589163 | 8.4281852 | 18.0164896 |  |
| H | 33.5938154 | 6.0971822 | 15.3213433 | H | 33.6004177 | 6.0889919 | 15.3357668 |  |
| H | 33.0434015 | 10.0536024 | 18.3012021 | H | 33.0281466 | 10.0755089 | 18.2669872 |  |
| H | 35.4088292 | 7.7741419 | 15.5255041 | H | 35.4176798 | 7.7463882 | 15.5542794 |  |
| H | 36.0890025 | 9.6948233 | 16.4765155 | H | 36.0698054 | 9.7008803 | 16.4686771 |  |
| H | 30.4605217 | 9.8130864 | 13.9898855 | H | 30.4654554 | 9.8244521 | 14.0003695 |  |
| H | 32.0899813 | 9.6811626 | 14.7000921 | H | 32.0996549 | 9.6714218 | 14.6949270 |  |
| H | 31.8860520 | 10.3017814 | 13.0298533 | H | 31.8863890 | 10.3005213 | 13.0286559 |  |
| H | 30.9965315 | 8.0377143 | 12.3317725 | H | 30.9656933 | 8.0463843 | 12.3316039 |  |
| H | 31.2484214 | 7.4403423 | 13.9956005 | H | 31.2313353 | 7.4417643 | 13.9906579 |  |
| H | 33.7573875 | 8.4507266 | 13.2152033 | H | 33.7334164 | 8.4674992 | 13.1550754 |  |
| H | 32.8092063 | 4.9019648 | 11.5212461 | H | 32.7942262 | 4.8520288 | 11.6043478 |  |
| H | 31.5337139 | 5.8488753 | 12.2070256 | H | 31.5103329 | 5.8334366 | 12.2224822 |  |
| H | 35.0326183 | 5.7076129 | 11.5480275 | H | 35.0162270 | 5.6746357 | 11.5932749 |  |
| H | 35.3607535 | 7.2731065 | 12.3014166 | H | 35.3416483 | 7.2508328 | 12.3149236 |  |
| H | 33.2606233 | 14.7577301 | 14.7848131 | H | 33.2682756 | 14.8018365 | 14.8242128 |  |
| H | 34.0454701 | 16.2669732 | 14.2586571 | H | 34.0680478 | 16.2688826 | 14.2075105 |  |
| H | 33.9405864 | 14.8790384 | 13.1539876 | H | 33.9100941 | 14.8317957 | 13.1736626 |  |
| H | 36.4270033 | 16.5665684 | 14.7008993 | H | 36.4460447 | 16.5551075 | 14.6363623 |  |
| H | 34.7225520 | 12.6081506 | 14.8516275 | H | 34.7121037 | 12.6150279 | 14.9018288 |  |
| H | 38.6433278 | 15.6388550 | 15.3874430 | H | 38.6605942 | 15.6284255 | 15.3330893 |  |
| H | 36.9334745 | 11.6915275 | 15.5161326 | H | 36.9284311 | 11.7036755 | 15.5769922 |  |
| H | 39.7983606 | 13.6250742 | 16.0605799 | H | 39.7827075 | 13.6616612 | 16.0522573 |  |
| H | 35.0092860 | 13.3710479 | 8.6134054 | H | 35.1177601 | 13.3232468 | 8.5496489 |  |
| H | 35.3074188 | 14.3792273 | 10.0486495 | H | 35.1638826 | 14.3288712 | 10.0155281 |  |
| H | 36.6750053 | 13.7402283 | 9.1224728 | H | 36.6775234 | 13.8463533 | 9.2322894 |  |
| H | 37.1977397 | 13.0621044 | 11.6892375 | H | 37.1429288 | 13.0937429 | 11.7446878 |  |
| H | 34.1661884 | 11.1916971 | 9.2562063 | H | 34.2925514 | 11.1340200 | 9.1666480 |  |
| H | 37.1940419 | 11.0941079 | 13.1945083 | H | 37.1132060 | 11.1390336 | 13.2608798 |  |
| H | 34.1564418 | 9.2217198 | 10.7802721 | H | 34.2568328 | 9.1737588 | 10.7043932 |  |
| H | 36.2578118 | 9.1310181 | 13.7463142 | H | 36.1013108 | 9.1571930 | 13.7964960 |  |
| H | 38.7633643 | 4.7387270 | 10.0092219 | H | 38.8003195 | 4.8420849 | 10.1953675 |  |
| H | 37.8442346 | 6.1216652 | 11.9394871 | H | 37.5947047 | 6.3876072 | 11.8070339 |  |
| H | 39.0571101 | 7.0449437 | 11.0650093 | H | 38.8157980 | 7.2789384 | 10.9166744 |  |
| H | 37.3363867 | 7.3913048 | 10.7940621 | H | 37.1059383 | 7.4367639 | 10.4502165 |  |
| H | 38.8867168 | 10.9913798 | 16.7130813 | H | 39.7324277 | 10.0753263 | 14.1589130 |  |
| H | 39.5941174 | 10.6613353 | 14.6080638 | H | 41.3924848 | 8.2444253 | 14.3196521 |  |
| H | 41.7277388 | 10.0864636 | 14.7119301 | H | 40.3830586 | 7.4036266 | 15.5434292 |  |
| H | 41.0703328 | 8.5782721 | 14.0564815 | H | 41.4322673 | 8.9192219 | 17.2694180 |  |
| H | 41.3878366 | 7.5498440 | 16.4284363 | H | 41.9174660 | 10.0612930 | 15.9767845 |  |
| H | 41.9733357 | 9.1345536 | 16.9846704 | H | 44.6780642 | 7.3415166 | 17.7548002 |  |
| H | 43.9567209 | 7.3910954 | 17.7794742 | H | 45.4035429 | 8.8991057 | 17.3076000 |  |
| H | 44.7389837 | 8.9559782 | 17.4229310 | H | 47.1288092 | 7.2461722 | 17.7650916 |  |
| H | 46.3713895 | 7.2952372 | 17.9529662 | H | 46.9645563 | 8.4204738 | 15.0229419 |  |
| H | 46.6089840 | 8.4685034 | 15.2379283 | H | 49.0496947 | 8.0258574 | 16.9429627 |  |
| H | 48.5282852 | 7.6366392 | 17.2134903 | H | 47.3569724 | 6.4516394 | 14.0171712 |  |
| H | 46.7374630 | 6.3885985 | 14.2483236 | H | 49.8925782 | 6.4123989 | 15.3093472 |  |
| H | 49.3131411 | 6.2010949 | 15.4810365 | H | 48.3699322 | 5.1614487 | 16.5945673 |  |
| H | 47.7077333 | 5.1507789 | 16.8693445 | H | 48.5914416 | 0.9721730 | 18.1915233 |  |
| H | 48.5879565 | 0.9847358 | 18.1921118 | H | 46.7500676 | -0.2574608 | 13.3993023 |  |
| H | 46.7702630 | -0.2651225 | 13.3899736 | H | 47.4738330 | -1.4362594 | 14.4522489 |  |
| H | 47.6255149 | -1.4082920 | 14.3979733 | H | 46.4478505 | 4.6237387 | 13.3169178 |  |
| H | 45.7319499 | 4.5320253 | 13.6103724 | H | 41.9247529 | 4.7750123 | 16.2887818 |  |
| H | 41.9474163 | 4.8403649 | 16.3259119 | H | 41.8667293 | 3.2194951 | 17.1339853 |  |
| H | 41.8365574 | 3.2350269 | 17.0445319 | H | 42.9079526 | 5.8400697 | 18.3822522 |  |
| H | 42.9040651 | 5.7006406 | 18.5551649 | H | 43.9343208 | 4.5378577 | 17.7283429 |  |
| H | 43.9350500 | 4.4979375 | 17.7433365 | H | 43.0394055 | 3.2028495 | 19.3860734 |  |
| H | 43.0491497 | 2.9701945 | 19.2466625 | H | 47.2551241 | 3.6252117 | 19.9115810 |  |
| H | 47.3344858 | 3.6730892 | 19.8108362 | H | 45.5253469 | 4.1363353 | 19.7918546 |  |
| H | 45.5693609 | 4.0543068 | 19.8881738 | H | 46.7080889 | 4.7164625 | 18.6058079 |  |
| H | 46.5742795 | 4.7472524 | 18.6131505 | H | 48.3795811 | 4.8481928 | 21.4495639 |  |
| H | 48.4039165 | 4.8667294 | 21.4202141 | H | 48.9748135 | 6.5026565 | 22.9298014 |  |
| H | 48.9096418 | 6.4697809 | 22.9985931 | H | 48.3565951 | 7.6879218 | 21.7374169 |  |
| H | 48.4508078 | 7.6526353 | 21.7298543 | H | 47.2587349 | 7.0093716 | 22.9776278 |  |
| H | 47.2306213 | 7.0817074 | 22.9064940 | H | 41.3088144 | 12.8210215 | 17.5512874 |  |
| H | 41.6876082 | 12.5089531 | 17.3165879 | H | 44.3464298 | 7.2491190 | 19.8768643 |  |
| H | 44.3202531 | 7.2058713 | 20.0166894 | H | 43.4288199 | 8.4297339 | 19.3445803 |  |
| H | 43.2820116 | 8.3262151 | 19.5582276 | H | 43.2531062 | 10.6435604 | 19.7599657 |  |
| H | 43.1672909 | 10.4905635 | 19.8201289 | H | 43.9172509 | 10.5476784 | 18.3677053 |  |
| H | 42.6548531 | 10.6270514 | 18.3425643 | H | 51.1920969 | 4.3944172 | 18.6477177 |  |
| H | 51.4092477 | 4.4118802 | 18.6930374 | H | 48.7929918 | -6.7464692 | 16.1394224 |  |
| H | 48.7904515 | -6.7517230 | 16.1369381 | H | 51.7155851 | -1.9152280 | 16.2763358 |  |
| H | 51.7183949 | -1.8815348 | 16.3053574 | H | 49.1580417 | 9.6845042 | 19.9436563 |  |
| H | 49.0731928 | 9.6759086 | 20.0702318 | H | 38.3354979 | 5.8801382 | 8.8273370 |  |
| H | 39.4331624 | 9.5271051 | 17.2917923 | H | 37.0912369 | 4.9772837 | 9.7196349 |  |
| H | 38.2712975 | 6.0015933 | 8.8582279 | H | 40.4719281 | 4.7734996 | 18.8531039 |  |
| H | 37.0380789 | 5.0723813 | 9.7378772 | H | 41.9938548 | 11.4598555 | 17.8871322 |  |
| H | 40.4991934 | 4.8548720 | 18.8642458 | H | 39.7898457 | 11.0200614 | 16.3480821 |  |
| H | 41.0179256 | 11.1506468 | 16.9576113 | H | 39.2234941 | 9.5710375 | 17.0376042 |  |
